# Total Synthesis of Piperine and Derivatives: Antimicrobial and Cytotoxic Activities

**DOI:** 10.1021/acsomega.6c03524

**Published:** 2026-06-23

**Authors:** Jenifer Reine Ngnouzouba Kuete, Vaderament-Alexe Nchiozem-Ngnitedem, Eric Sperlich, Birthe Sandargo, Léon Azefack Tapondjou, Rémy Bertrand Teponno, Bernd Schmidt

**Affiliations:** † Department of Chemistry, Faculty of Science, 362702 University of Dschang, Dschang, P.O. Box 67 67, Cameroon; ‡ Institut Für Chemie, 26583 University of Potsdam, Potsdam-Golm D-14476, Germany; § Department Microbial Drugs, Helmholtz Centre for Infection Research (HZI), Braunschweig 38124, Germany

## Abstract

Piperine is a secondary metabolite derived from plants and fungi and exhibits a wide range of biological properties including antimicrobial, anticancer, anti-inflammatory, and antidiabetic activities. In the present study, the total synthesis of piperine (**7a**) and related analogues **7b**-**l** was achieved starting from (*E*)-3,4-(methylenedioxy)­cinnamic acid (**5**) through PyBOP-mediated coupling with various amines **6**. Seven of these analogues (compounds **7d**, **7f**–**j** and **7l**) are hereby synthesized for the first time. Piperine (**7a**) displayed antifungal activity against *Rhodotorula glutinis* DSM-10134 (MIC value of 16.6 μg/mL) while piperic acid (**5**), piperine (**7a**), and its analogue **7d** were active against the fungus *Mucor hiemalis* DSM2656 with MICs of 66.6, 33.3, and 33.3 μg/mL, respectively. Furthermore, the analogue **7d** exhibited moderate activity against some cancer cell lines with IC_50_ values ranging from 7 to 21 μg/mL.

## Graphical abstract

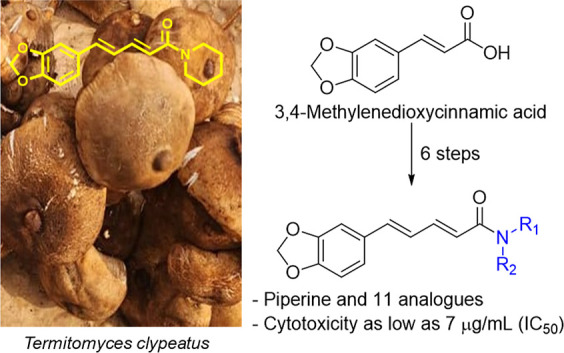

## Introduction

Piperine is an alkaloid originally isolated from black pepper, *Piper nigrum*.^1^ Later, piperine was also isolated from other plants of the piperaceae family, and several bioactivities, such as anticancer^2^ and antitubercular activity have been reported.^3^ Reports on the isolation of piperine from other natural sources, in particular nutritional plants, are scarce. Some of us have recently discovered that piperine is a phytochemical constituent of the edible mushroom *Termitomyces clypeatus*,^4^ which belongs to the Lyophyllaceae family, comprising approximately 160 species distributed across eight genera. These mushrooms are found in several parts of the world, particularly in Central Africa and Southeast Asia^5^ and are widely consumed as food and seasoning throughout equatorial and southern Africa, as well as Southeast Asia.^6^ Besides their socioeconomic impact in several African and Asian countries, some species are well-known to have medicinal importance. Notably, *T. microcarpus* is used to treat gonorrhea, while *T. clypeatus* is used for the treatment of measles and yellow fever.^7^ In addition, scientific evidence has shown that *T. clypeatus* possesses antibacterial, antioxidant, immunomodulatory, anticancer, and antitumor activities.^8,9^

The finding that piperine is a secondary metabolite present in *T. clypeatus* and the reported health benefits of this nutritional mushroom prompted us to investigate cytotoxic and antifungal activities of this compound. To expand the chemical library of piperine analogs with potentially interesting and improved pharmacological properties, a synthetic approach becomes crucial. Although both the total synthesis and semisynthesis of piperine have previously been reported^10–12^ and reviewed,^13^ pursuing a dedicated synthetic study is important to ensure reliable access to sufficient quantities of material, as well as to provide a strategic platform for structural diversification and further functional explorations to elucidate their biological potential.

## Results and Discussion

Initially, we planned to synthesize piperic acid (**5**), required as a key intermediate for the synthesis of piperine (**7a**) and its analogues, following a literature procedure^14^ through Horner–Wadsworth–Emmons olefination of piperonal with (2*E*)-triethylphosphonocrotonate. Unfortunately, this approach turned out to be unsuccessful in our hands, and we therefore, adapted a route previously reported for the synthesis of structurally related avenalumic acid derivatives.^15^ Starting from (*E*)-3,4-(methylenedioxy)­cinnamic acid (**1a**) the corresponding methyl ester **1b** was synthesized using trimethylsilyl chloride in methanol.^16^ Subsequent reduction of the ester **1b** with diisobutylaluminum hydride (DIBAL-H) in tetrahydrofuran produced the corresponding allylic alcohol **2** in high yield, which was oxidized with MnO_2_ to furnish the aldehyde **3**. Horner-Wadsworth-Emmons olefination^17^ with triethylphosphonoacetate (TEPA) in the presence of NaH as a base afforded the conjugated ester **4**, which was eventually hydrolyzed under basic conditions to yield piperic acid (**5**) in quantities of up to 4 g per batch over five steps in a total yield of 75% (Scheme 1).

**Scheme 1:**
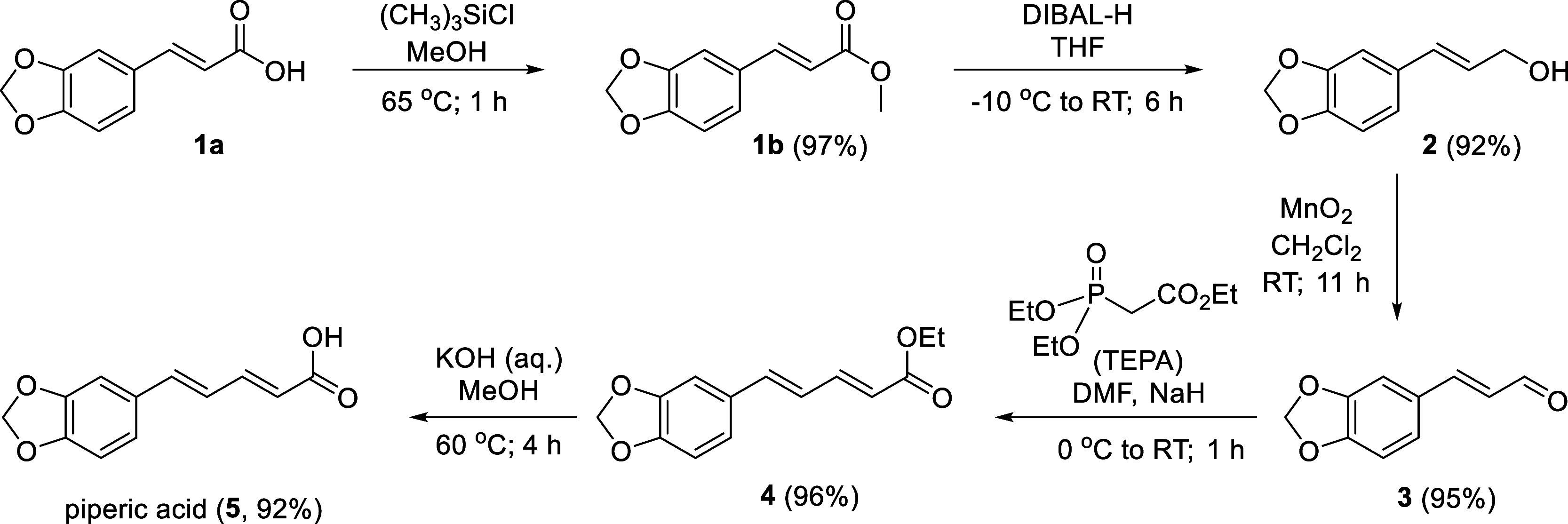
Synthesis of Piperic Acid (**5**)

Having the requisite key building block piperic acid (**5**) successfully synthesized, the final step toward piperine (**7a**), piperlonguminine (**7b**) and their non-natural derivatives **7c**-**7l** was carried out under conditions similar to those previously reported by some of us,^18^ using the amide coupling reagent PyBOP (Scheme 2).^19^ Piperine (**7a**), originally isolated from black pepper, but also present in numerous other plant species, has attracted considerable attention due to its numerous bioactivities, such as analgesic, anti-inflammatory, antioxidant or cytotoxic activities.^20^ It has earlier been synthesized through amide coupling of **5** and piperidine (**6a**) using either Steglich’s method^21,22^ or an in situ formed mixed anhydride,^23^ but not under the conditions used in this work. Piperlonguminine (**7b**), a secondary metabolite from *Piper longum* and several other *Piper* species, has also attracted considerable attention due to its numerous bioactivities^24^ and was previously synthesized from **5** and isobutylamine (**6b**) using Steglichs method^21^ or a mixed anhydride.^25^ The non-natural analogues **7c** and **7e** were earlier synthesized through in situ formation of a mixed anhydride of **5** and methanesulfonic acid, and subsequent reaction with morpholine (**6c**) and benzylamine (**6e**), respectively. Both have been tested for their effect on melanocyte proliferation and differentiation,^25^ and **7c** as a *Staphylococcus aureus* NorA efflux pump inhibitor.^26^ Compound **7k** had been synthesized from the acid chloride of **5** and primary amine **6k** for testing as a GABA_A_-receptor modulator^27^ and as an inhibitor of the *P*-glycoprotein drug transporter, that is responsible for the occurrence of drug resistance against several cytotoxic chemotherapeutic agents.^28^ Carboxamides **7d**, **7f**–**j**, and **7l** are novel compounds that have not been reported in the literature according to a SciFinder search.

**Scheme 2:**
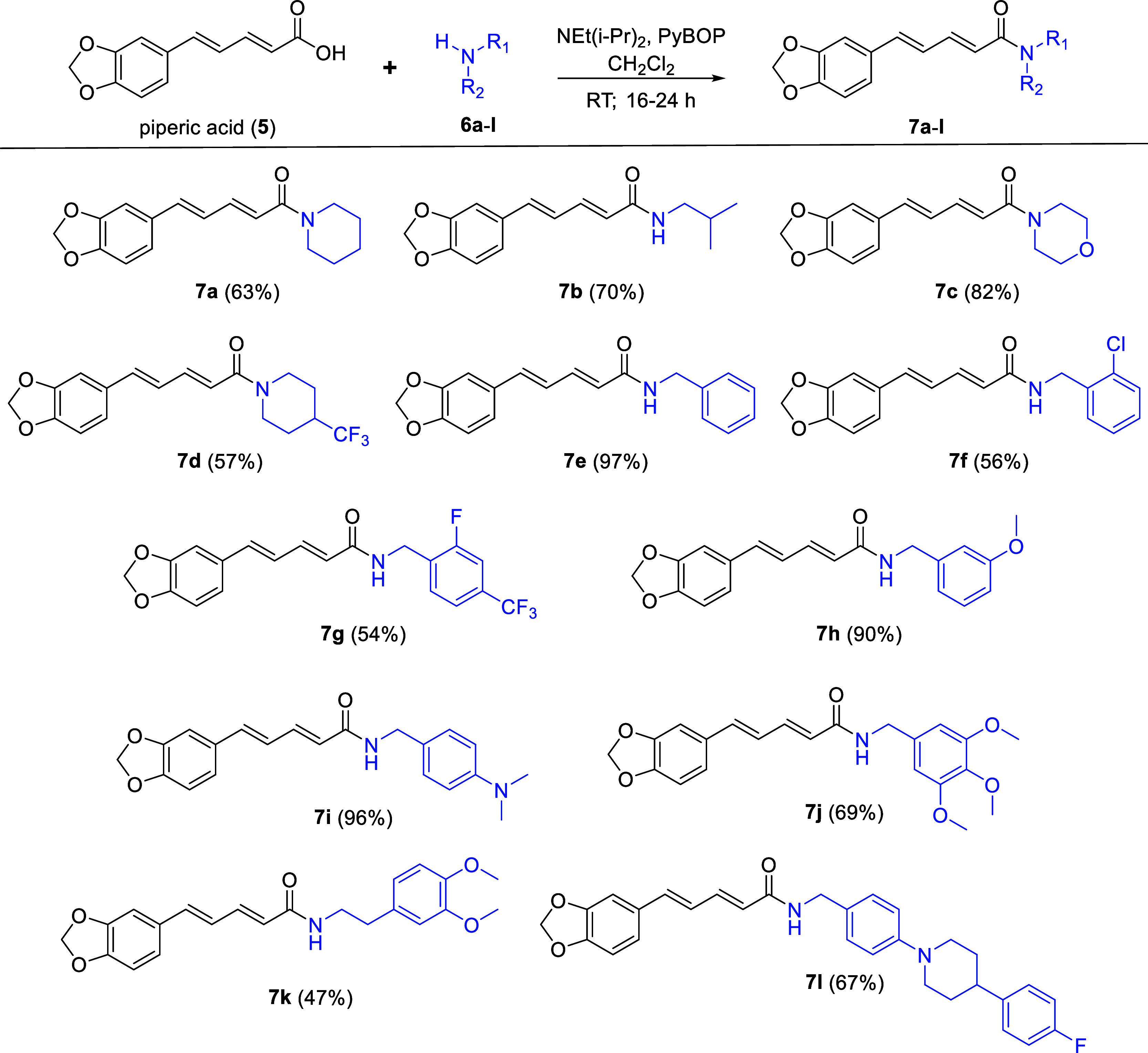
Synthesis of Piperic Acid Amides **7**

The molecular structure of synthetic piperine (**7a**) was unambiguously corroborated by single crystal X-ray structure analysis (Figure 1). The molecular structure of naturally occurring piperine isolated from black pepper had previously been determined by crystallography.^29^

**Figure 1:**
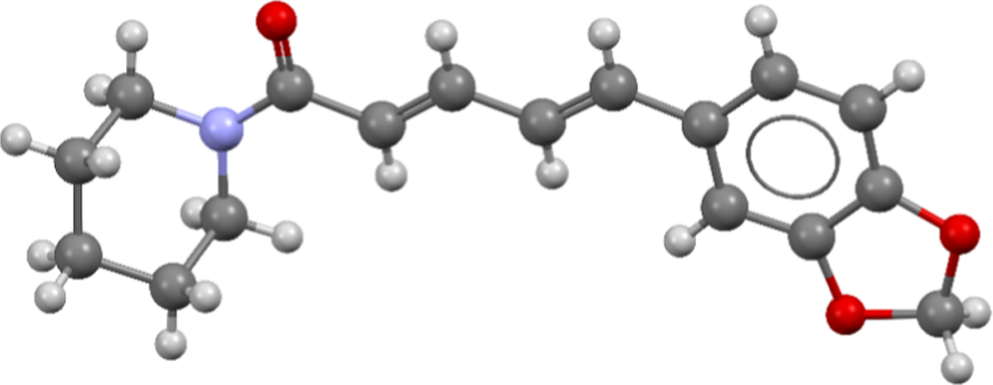
Single-crystal X-ray structure of synthetic piperine (**7a**).

Since piperine (**7a**) and its derivatives are reported to possess a huge spectrum of biological properties,^30^ we first investigated the antimicrobial activity of compounds **5** and **7a**-**7l** against a range of 12 different fungi and bacteria (see Supporting Information, Table S2, for details). Several investigations into the antimicrobial activity of piperic acid (**5**) and piperine (**7a**) have been reported.^31–33^ For example, piperine (**7a**) has previously been shown to inhibit the growth of multidrug-resistant strains of *Pseudomonas aeruginosa* and *Escherichia coli* at a concentration of 200 μg·mL^–1^ (700.9 μM). We could detect notable antimicrobial activities only in four cases: compound **7d**, a piperine analogue with a trifluoromethyl group attached to the 4-position of the piperidine ring, is active against the pathogenic fungi *Mucor hiemalis* DSM2656 and *Rhodotorula glutinis* DSM10134 at minimum inhibitory concentrations of 33.3 μg·mL^–1^ (94.2 μM), and piperine (**7a**) is active against the same fungi with MIC values of 33.3 μg·mL^–1^ (116.7 μM) against *M. hiemalis* DSM 2656 and 16.6 μg·mL^–1^ (58.4 μM) against *R. glutinis* DSM 10134. Reports on the evaluation of piperine (**7a**) and structurally related natural or synthetic carboxamides against pathogenic fungi in general are comparatively scarce. In particular, piperine (**7a**) and piperine derivatives have not been tested against *M. hiemalis* or *R. glutinis* before, but there are a few reports describing activity of piperine (**7a**) and its derivatives against *Cladosporium* sp.,^34–37^ *Candida albicans* and *Candida krusei*,^38^ and against some phytopathogenic fungi.^39^ The antimicrobial activity of piperine was attributed to its ability to disrupt and penetrate the microbe cell wall and reach the intracellular environment. This penetration is facilitated by its lipophilic nature and interaction with the lipid components of the cell membrane, which increases the fluidity and permeability of the cell membrane leading to the leakage of intracellular components such as ions, proteins, and metabolites, further compromising the normal cellular functions of microorganisms and causing cell death.^40^ Among the amides tested, only piperine (**7a**), compounds **7c**, **7d**, and **7g** showed MIC values less than or equal to 66.6 μg·mL^–1^ against one or more microbial strains. Unlike piperine, whose antimicrobial activity has already been proven and which comprises a piperidine ring, compound **7c** has a morpholine core and it was shown that molecules possessing this ring exhibited a large spectrum of biological activities, including the inhibition of microbial protein synthesis.^41^ Compounds **7d** and **7g** have a trifluoromethyl group in their structures. Although they are not more active than the natural product piperine, it has been noted that fluorine-containing compounds now constitute around a quarter of small molecule drugs in the pharmaceutical market and trifluoromethyl group is one of the most widely used fluorinated moieties since its presence increases not only the lipophilicity, but also the stability of molecules.^42,43^ However, compound **7g** has in addition to the trifluoromethyl group, an additional fluorine atom linked to the aromatic ring which would make it even more lipophilic compared to compound **7d**. This would suggest that the excess lipophilicity could also reduce the antimicrobial activity.

Piperic acid (**5**) and the carboxamides **7a**-**l** were then tested against the panel of cancer cell lines shown in Table 1. All compounds were initially screened against murine fibroblasts L929 and human cervix carcinoma KB-3-1 cells, but only **7d** and **7j** showed notable activities against both of these cell lines, with IC_50_ values below 20 μg·mL^–1^. Based on this preliminary result, the influence of the trifluoromethyl group in compound **7d** was more comprehensively evaluated against four other cell lines, i.e. breast cancer MCF-7, epidermoid carcinoma A431, human lung carcinoma A549, and human prostate cancer PC-3 cells. For **7d**, IC_50_ values are in the range of 7.0 to 21 μg·mL^–1^ (19.8–59 μM) for all six cell lines tested. Activity of carboxamide **7j** against murine fibroblasts L929 and human cervix carcinoma KB-3-1 cells is similar to that of **7d**, with IC_50_ values of 15 and 9.4 μg·mL^–1^ (37.7 and 23.7 μM), respectively. Earlier investigations into the cytotoxic activity of piperine (**7a**) and structurally related natural products have been reviewed.^33,44,45^ Recent examples are the inhibition of migration and invasion of human pancreatic PANC-1 cells by piperine (**7a**),^46^ the inhibitory effect of the carboxamide natural products retrofractamides A and B, and chabamide, a Diels–Alder-dimer of piperine, on MCF-7 breast cancer cells with IC_50_ values between 36 and 56 μM,^47^ the cytotoxic activity of kunstleramide and derivatives, inter alia against MCF-7 and A549 cells, with IC_50_ values in the range of 12 to 30 μM,^48^ and the recently reported inhibition of MCF-7 cells by a non-natural *ortho*-hydroxyphenyl amide of piperic acid in a dose-dependent manner (IC_50_: 55 μM).^49^ All in all, compound **7d** represents a promising lead for future optimization, after which detailed mechanistic investigations could be undertaken. For comparison, epothilone B exhibited sub nanomolar range across all tested cells lines.

**Table 1:** Cytotoxicity (IC_50_) of Compounds **5**, **7a**-**7l** against Various Cell Lines

	IC_50_ in μg·mL^–1^ (μM)^a^					
compounds	L929	KB3.1	MCF-7	A431	A549	PC-3
**5**	>37 (>170)	>37 (>170)	-	-	-	-
**7a**	32 (112)	23 (81)	-	-	-	-
**7b**	25 (91)	18 (66)	-	-	-	-
**7c**	>37 (>129)	>37 (>129)	-	-	-	-
**7d**	17 (48)	7.0 (19.8)	9.0 (25.5)	21 (59)	18.5 (52)	13 (36.8)
**7e**	21 (68)	>37 (>120)	-	-	-	-
**7f**	>37 (>108)	>37 (>108)	-	-	-	-
**7g**	>37 (>94)	>37 (>94)	-	-	-	-
**7h**	>37 (>110)	18 (53)	-	-	-	-
**7i**	>37 (>106)	25 (71)	-	-	-	-
**7j**	15 (37.7)	9.4 (23.7)	-	-	-	-
**7k**	>37 (>97)	>37 (>97)	-	-	-	-
**7l**	>37 (>76)	>37 (>76)	-	-	-	-
**epothilone B**	0.00098	0.000028	0.001300	0.000220	0.000110	0.000080

a “-”: not tested.

The anticancer mechanism of action of the active compounds **7d** and **7j** could, as in the case of piperine, involve the activation of cellular and molecular signaling pathways with programmed cell death, decreased migration and invasion, and reduced cell proliferation.^50,51^ Compounds **7d** and **7j** were found to be more active against the murine fibroblasts L929 and human cervix carcinoma KB-3-1 cells with IC_50_ values below 20 μg·mL^–1^. It has already been demonstrated that the presence of the trifluoromethyl group considerably increases cytotoxic activity of molecules.^52^ However, it is surprising to note that compound **7g**, which also features this group, did not exhibit activity at a concentration less than or equal to 37 μg·mL^–1^. Of the 13 compounds tested, seven (**7e**–**j**, **7l**) have in their structures the benzyl group linked to the nitrogen of the amide function. The difference in observed activity could be due to the fact that these benzylic groups do not carry the same substituents nor the same substitution patterns. In this series, compound **7j** which possesses three methoxy groups attached to the benzyl ring was the most active. This is not surprising because it was reported that methoxy-substituted compounds such as the diarylheptanoid curcumin derived from *Curcuma longa* and combretastatin A-4 derived from *Combretum caffrum* display anticancer effects.^53^

The computational tool QikProp from Schrödinger Maestro-2023-2 ^54^ showed that all compounds complied with Lipinski’s Rule of Five (Table 2). The structure–activity relationship (SAR) analysis of compounds **7a**–**7l** demonstrated that the nature of the amide substituent, lipophilicity, and electronic characteristics strongly influenced the biological activity. Compound **7a**, bearing a piperidine moiety, showed moderate activity with acceptable physicochemical properties (QPlogP = 3.25). Replacement of the piperidine ring with a morpholine moiety in compound **7c** (QPlogP = 2.14) resulted in comparatively lower activity, which may be attributed to the presence of the oxygen atom in the morpholine ring that increases polarity and reduces hydrophobic interactions with the target binding site. Introduction of the electron-withdrawing trifluoromethyl (–CF_3_) substituent in compound **7d** significantly enhanced activity relative to **7a**. Compound **7d** exhibited higher lipophilicity (QPlogP = 4.27), which may favor stronger hydrophobic and van der Waals interactions within the receptor binding pocket. Although Caco-2 permeability contributes to intestinal absorption, the superior activity of **7d** suggests that receptor binding affinity and optimal hydrophobic balance play a more dominant role than permeability alone. The electron-withdrawing–CF_3_ group may additionally influence molecular conformation and electronic distribution, thereby improving target interaction. In contrast, compound **7l**, which possessed very high lipophilicity (QPlogP = 7.24), exhibited reduced activity compared with **7d**. This observation indicates that moderate lipophilicity is optimal for biological activity, whereas excessive lipophilicity becomes unfavorable. Compounds containing substituted aromatic rings such as **7e**–**7k** in some cases demonstrated improved activity compared with simple aliphatic analogs, likely due to enhanced π–π stacking and hydrophobic interactions with the biological target. Electron-withdrawing substituents such as chloro, fluoro and trifluoromethyl groups (**7f** and **7g**) increased lipophilicity and membrane permeability; however, highly hydrophobic derivatives exhibited lower predicted solubility, which may negatively affect activity. Conversely, methoxy-substituted derivatives (**7h**, **7j**, and **7k**) may benefit from balanced electronic effects and additional hydrogen-bonding interactions, contributing to favorable receptor binding.

**Table 2:** Physicochemical Properties Analysis of Compounds (**5, 7a**–**7l**)

		drug likeliness (Lipinski’s rule of 5)				ADME properties by QikProp			
compound	M_W_ ^a^	donor HB^b^	acceptor HB^c^	QPlogP_o/w_ ^d^	violation of Lipinski’s rule	QP logBB^e^	QPP Caco^f^	QP logS^g^	% human absorption^h^
**5**	219.07	1	3.50	2.19	0	–0.69	203.83	–2.74	81.11
**7a**	285.14	0	4.50	3.25	0	–0.09	4223.47	–3.55	100.00
**7b**	273.14	1	4.00	3.47	0	–0.33	3354.84	–3.65	100.00
**7c**	288.12	0	6.20	2.14	0	–0.09	4062.84	–2.87	100.00
**7d**	353.12	0	4.50	4.27	0	–0.14	4094.69	–5.00	100.00
**7e**	307.12	1	4.00	4.12	0	–0.30	3670.75	–4.69	100.00
**7f**	342.09	1	4.00	4.54	0	–0.15	3803.90	–5.39	100.00
**7g**	394.11	1	4.00	5.30	1	–0.04	3680.60	–6.44	100.00
**7h**	338.14	1	4.75	4.22	0	–0.37	3563.33	–5.00	100.00
**7i**	351.17	1	5.00	4.55	0	–0.40	3413.45	–517	100.00
**7j**	398.16	1	6.25	4.50	0	–0.46	4030.97	–5.62	100.00
**7k**	382.16	1	5.50	4.69	0	–0.61	3046.98	–5.60	100.00
**7l**	484.22	1	5.00	7.24	1	–0.26	3689.39	–8.32	100.00

a Molecular weight (acceptable range: <500).

b Hydrogen bond donor (acceptable range: <5).

c Hydrogen bond acceptor (acceptable range: <10).

d Predicted octanol/water partition coefficient log P (acceptable range from −2.0 to 6.5).

e Predicted brain/blood partition coefficient (acceptable range from −3.0–1.2).

f Predicted Caco-2 cell permeability in nm/s (acceptable range: <25 is poor and >500 is good).

g Predicted aqueous solubility in mol/L (acceptable range: −6.5 to 0.5).

h Percentage of human oral absorption (<25% is poor, and >80% is high).

## Conclusion

Gram quantities of piperic acid (**5**) were synthesized from commercially available (*E*)-3,4-(methylenedioxy)­cinnamic acid (**1a**) in five steps and 75% overall yield. A major advantage over the established route is that piperonal, a restricted substance that is increasingly difficult to purchase in many countries, can be avoided as a starting material. Through PyBOP-facilitated amide coupling of piperic acid with various primary and secondary amines we synthesized the naturally occurring alkaloids piperine (**7a**) and piperlonguminine (**7b**), as well as ten non-natural analogues, out of which seven had not been reported previously. All compounds were comprehensively evaluated for their antimicrobial activity against pathogenic fungi and bacteria, and for their cytotoxic activity against six human cancer cell lines. While no significant antibacterial activity could be detected for any of the compounds, two compounds, piperine (**7a**) and a novel trifluoromethyl-substituted piperine derivative **7d**, were found to be moderately active against the pathogenic fungi *M. hiemalis* DSM2656 and *R. glutinis* DSM10134. Two of the novel piperic acid carboxamides, **7d** and **7j**, displayed notable cytotoxic activities against murine fibroblasts L929, human cervix carcinoma KB-3.1, breast cancer MCF-7, epidermoid carcinoma A431, human lung carcinoma A549, and human prostate cancer PC-3 cells, with IC_50_ values ranging from 7.0 to 21.0 μg·mL^–1^ (19.8 to 59 μM). Although the presence of a CF_3_-group at position 4′ of the piperidine ring is the only structural difference between the well-studied natural product piperine (**7a**) and its non-natural derivative **7d**, we observed a substantial effect of this structural modification on both antifungal and cytotoxic activity, as the parent compound piperine (**7a**) was found to be virtually inactive in all bioassays studied herein.

Given that piperic acid and it is carboxamides are now accessible in large amounts, we plan in our future investigations to conduct further biological tests with those derivatives that have shown promising activity and design further novel derivatives based on the conclusions drawn from this investigation.

## Experimental Section

### General Experimental Procedures

All reactions were carried out in dry reaction vessels. All solvents were purified by standard procedures. Melting points were measured using a SMP-10 instrument of Bibby Scientific (Stuart). IR spectra were recorded as ATR-FTIR spectra using a PerkinElmer UART TWO FT-IR-spectrometer. Wavenumbers (ν) are given in cm^–1^. The peak intensities are defined as strong (s), medium (m), or weak (w). NMR spectra were recorded in CDCl_3_, CD_3_OD, or (CD_3_)_2_SO using a Bruker NEO-400 instrument operating at 400 and 100 MHz for ^1^H and ^13^C­{^1^H} NMR, respectively. Spectra referencing was accomplished using the residual CHCl_3_ solvent peak for ^1^H and the CDCl_3_ solvent peak for ^13^C NMR spectra (δ = 7.26 and 77.1 for ^1^H and ^13^C­{^1^H} signals, respectively), CD_2_HOD solvent peak for ^1^H and the CD_3_OD solvent peak for ^13^C NMR spectra (δ = 3.51 and 40.0 for ^1^H and ^13^C­{^1^H} signals, respectively), and residual (CD_2_H)­SO­(CD_3_) solvent peak for ^1^H and the (CD_3_)_2_SO solvent peak for ^13^C NMR spectra (δ = 2.50 and 39.5 for ^1^H and ^13^C­{^1^H} signals, respectively). Multiplicities in ^1^H NMR spectra were described using the common descriptors s (singlet), d (doublet), t (triplet), q (quartet), or m (multiplet). Whenever complex fine splitting of the individual lines of a signal due to long-range coupling was observed, the descriptor “m” was added to the multiplicity descriptor. High-resolution mass spectra were obtained by EI-TOF (70 eV) or ESI-Q-TOF using Waters Micromass instruments. Amine **6l** was synthesized following a literature procedure.^55^

#### Methyl (*E*)-3-(Benzo­[*d*]­[1,3]­dioxol-5-yl)­acrylate (**1b**)

Chlorotrimethylsilane (2.20 mL, 17.3 mmol) in MeOH (45 mL) was stirred under N_2_ atmosphere at 0 °C for 20 min. Subsequently, 3,4-methylenedioxycinnamic acid (**1a**, 2.21 g, 11.5 mmol) was added, and the mixture was refluxed at 65 °C for 1h. The white precipitate was filtered, washed with H_2_O and dried under vacuum to afford **1** (2.30 g, 11.2 mmol, 97%): white powder; IR (ATR) ν̃ 2905 (w), 1701 (s), 1623 (s), 1599 (s), 1453 (s), 1252 (s), 1169 (s); ^1^H NMR (400 MHz, CDCl_3_): δ 7.59 (d, *J* = 16.0 Hz, 1H), 7.02 (d, *J* = 2.0 Hz, 1H), 6.99 (dd, *J* = 8.0, 2.0 Hz, 1H), 6.81 (d, *J* = 8.0 Hz, 1H), 6.26 (d, *J* = 16.0 Hz, 1H), 6.00 (s, 2H), 3.79 (s, 3H); ^13^C­{^1^H} NMR (100 MHz, CDCl_3_): δ 167.8, 149.8, 148.5, 144.7, 128.9, 124.6, 115.9, 108.7, 106.6, 101.7, 51.8; HREIMS *m*/*z* 206.0568 [M^+^] (calcd for C_11_H_10_O_4_, 206.0574). Used in the next step without further purification. Spectroscopic data match those reported in the literature.^56^

#### (*E*)-3-(Benzo­[*d*]­[1,3]­dioxol-5-yl)­prop-2-en-1-ol (**2**)

To a solution of **1b** (2.70 g, 13.1 mmol) in THF (55 mL) was added under Ar atmosphere DIBAL-H (1.0 M in toluene, 32.8 mL, 32.8 mmol) dropwise (over 12 min) at −10 °C. Afterward, the solution was allowed to warm to room temperature for 6 h. The mixture was acidified to pH 5 with 2 M aq. HCl, followed by addition of H_2_O (60 mL). The aqueous phase was extracted with diethyl ether (twice, 50 mL). The combined organic layers were dried using anhydrous MgSO_4_, filtered, and evaporated in vacuo. The crude product was purified by column chromatography on silica using petroleum ether/ethyl acetate (3:1 (v/v)) to give **2** (2.13 g, 12.0 mmol, 92%): yellow powder; IR (ATR) ν̃ 3357 (s), 2888 (w), 1501 (s), 1489 (s), 1443 (s), 1247 (s), 1068 (s), 967 (s); ^1^H NMR (400 MHz, CDCl_3_): δ 6.92 (d, *J* = 1.7 Hz, 1H), 6.81 (dd, *J* = 8.0, 1.7 Hz, 1H), 6.75 (d, *J* = 8.0 Hz, 1H), 6.51 (dt, *J* = 15.8, 1.6 Hz, 1H), 6.19 (dt, *J* = 15.8, 5.9 Hz, 1H), 5.95 (s, 2H), 4.28 (dd, *J* = 5.9, 1.6 Hz, 2H), 1.74 (s­(br), 1H); ^13^C­{^1^H} NMR (100 MHz, CDCl_3_): δ 148.1, 147.4, 131.2, 131.1, 126.8, 121.3, 108.4, 105.9, 101.2, 63.8; HREIMS *m*/*z* 178.0620 [M^+^] (calcd for C_10_H_10_O_3_, 178.0625). Spectroscopic data match those reported in the literature.^57^

#### (*E*)-3-(Benzo­[*d*]­[1,3]­dioxol-5-yl)­acrylaldehyde (**3**)

A mixture of **2** (3.68 g, 20.7 mmol), MnO_2_ (26.88 g, 309 mmol) in CH_2_Cl_2_ (91 mL) was stirred at room temperature for 11 h. The excess of MnO_2_ was filtered through Celite. The solvent was evaporated, and the residue was purified by column chromatography on silica using petroleum ether/ethyl acetate (5:1 (v/v)) to furnish **3** (3.45 g, 19.6 mmol, 95%): white powder; IR (ATR) ν̃ 2836 (w), 1666 (s), 1624 (s), 1598 (s), 1490 (s), 1251 (s), 925 (s); ^1^H NMR (400 MHz, CDCl_3_) δ 9.60 (d, *J* = 7.7 Hz, 1H), 7.33 (d, *J* = 15.8 Hz, 1H), 7.03 (dd, *J* = 8.6, 1.7 Hz, 1H), 7.01 (d, *J* = 1.7 Hz, 1H), 6.82 (d, *J* = 8.6 Hz, 1H), 6.51 (dd, *J* = 15.8, 7.7 Hz, 1H), 6.00 (s, 2H); ^13^C­{^1^H} NMR (100 MHz, CDCl_3_): δ 193.5, 152.6, 150.5, 148.6, 128.5, 126.9, 125.3, 108.8, 106.8, 101.9; HREIMS *m*/*z* 176.0463 [M^+^] (calcd for C_10_H_8_O_3_, 176.0468). Spectroscopic data match those reported in the literature.^58^

#### Ethyl (2*E*,4*E*)-5-(Benzo­[*d*]­[1,3]­dioxol-5-yl)­penta-2,4-dienoate (**4**)

To a solution of **3** (3.45 g, 19.6 mmol) in DMF (20 mL), triethyl phosphonoacetate (TEPA) (4.70 mL, 23.5 mmol) was added. To this mixture, NaH (60-wt% dispersion in mineral oil, 940 mg, 39.2 mmol) was added at 0 °C. The mixture was stirred at room temperature for 1 h, poured into an ice water bath (50 mL), and extracted with diethyl ether (twice, 100 mL). The combined organic layers were dried with anhydrous MgSO_4_, filtered, and evaporated in vacuo. The residue was purified by column chromatography on silica using petroleum ether/ethyl acetate (95:5 (v/v)) to furnish **4** (4.62 g, 18.8 mmol, 96%): yellow powder; IR (ATR) ν̃ 2923 (w), 1704 (s), 1620 (m), 1609 (m), 1503 (m), 1489 (s), 1445 (s), 1244 (s), 1035 (s); ^1^H NMR (400 MHz, CDCl_3_) 7.41 (dd, *J* = 15.2, 10.8 Hz, 1H), 6.99 (d, *J* = 1.7 Hz, 1H), 6.90 (dd, *J* = 8.1, 1.7 Hz, 1H), 6.78 (d, *J* = 15.5, 1H), 6.77 (d, *J* = 8.1, 1H), 6.69 (dd, *J* = 15.5, 10.8 Hz, 1H), 5.98 (s, 2H), 5.93 (d, *J* = 15.2 Hz, 1H), 4.22 (q, *J* = 7.1 Hz, 2H), 1.31 (t, *J* = 7.1 Hz, 3H); ^13^C­{^1^H} NMR (100 MHz, CDCl_3_): δ 167.3, 148.7, 148.4, 144.8, 140.2, 130.7, 124.7, 123.0, 120.6, 108.6, 106.0, 101.5, 60.4, 14.5; HREIMS *m*/*z* 246.0885 [M^+^] (calcd for C_14_H_14_O_4_, 246.0887). Spectroscopic data match those reported in the literature.^59^

#### (2*E*,4*E*)-5-(Benzo­[*d*]­[1,3]­dioxol-5-yl)­penta-2,4-dienoic Acid (**5**)

To a solution of **4** (4.62 g, 18.8 mmol) in MeOH (44 mL), was added aq. KOH (15 wt-%, 44 mL). The mixture was refluxed at 60 °C for 4 h. After cooling, MeOH was evaporated H_2_O (100 mL) was added, and the solution was acidified to pH 4 with a 2 M aq. HCl. The aqueous layer was extracted with ethyl acetate (twice, 75 mL). The combined organic layers were washed with brine, dried with anhydrous MgSO_4_, filtered, and evaporated in vacuo. Recrystallization from petroleum ether/acetone (9/1 (v/v)) afforded **5** (3.78 g, 17.3 mmol, 92%): yellow powder; IR (ATR) ν̃ 2915 (w), 1672 (m), 1253 (m), 999 (m); ^1^H NMR (400 MHz, DMSO-*d*_6_): δ 12.16 (s­(br.), 1H), 7.37–7.26 (m, 1H), 7.23 (d, *J* = 1.6 Hz, 1H), 7.00 (dd, *J* = 8.1, 1.6 Hz, 1H), 6.98–6.93 (m, 2H), 6.92 (d, *J* = 8.0 Hz, 1H), 6.05 (s, 2H), 5.92 (d, *J* = 15.2 Hz, 1H); ^13^C­{^1^H} NMR (100 MHz, DMSO-*d*_6_): δ 167.6, 148.1, 148.0, 144.6, 139.8, 130.5, 124.9, 123.1, 121.1, 108.5, 105.7, 101.4; HRESIMS *m*/*z* 219.0652 [M + H]^+^ (calcd for C_12_H_11_O_4_, 219.0657). Spectroscopic data match those reported in the literature.^60^

### General Procedure for the Synthesis of Piperine and Derivatives

Piperic acid (**5**, 218 mg, 1.00 mmol) was dissolved in CH_2_Cl_2_ (10 mL). To this solution was added NEt­(*i*-Pr)_2_ (0.43 mL, 2.55 mmol), the corresponding amine **6** (1.70 mmol), and PyBOP (531 mg, 1.02 mmol). The mixture was stirred under an atmosphere of dry nitrogen for 15–24 h. The progress of the reaction was monitored by TLC. The mixture was filtered and evaporated in vacuo, and the residue was purified by column chromatography on silica using petroleum ether/ethyl acetate mixtures as eluents, as specified for the individual compounds, to yield the target molecules.

#### (2*E*,4*E*)-5-(Benzo­[*d*]­[1,3]­dioxol-5-yl)-1-(piperidin-1-yl)­penta-2,4-dien-1-one (Piperine, **7a**)

Following the general procedure, piperic acid **5** (218 mg, 1.00 mmol) and piperidine (**6a**, 168 μL, 1.70 mmol) were converted to **7a** (181 mg, 0.63 mmol, 63%): colorless crystals, mp 130–131 °C (reported in the literature: mp 132–133 °C^23^; IR (ATR) ν̃ 2939 (w), 2853 (w), 1632 (m), 1612 (m), 1584 (s), 1489 (s), 1440 (s), 1249 (s), 997 (s); ^1^H NMR (400 MHz, CD_3_OD): δ 7.32 (dd, *J* = 14.6, 10.1 Hz, 1H), 7.09 (d, *J* = 1.7 Hz, 1H), 6.96 (dd, *J* = 8.1, 1.7 Hz, 1H), 6.90 (dd, *J* = 15.5, 10.1 Hz, 1H), 6.82 (d, *J* = 15.5 Hz, 1H), 6.79 (d, *J* = 8.1 Hz, 1H), 6.62 (d, *J* = 14.6 Hz, 1H), 5.96 (s, 2H), 3.65–3.59 (m, 4H), 1.75–1.65 (m,, 2H), 1.65–1.53 (m, 4H); ^13^C­{^1^H} NMR (100 MHz, CD_3_OD): δ 167.7, 149.8, 149.8, 144.6, 140.1, 132.4, 126.4, 123.9, 120.7, 109.4, 106.7, 102.7, 48.1, 44.5, 27.9, 26.9, 25.6; HREIMS *m*/*z* 285.1357 [M^+^] (calcd for C_17_H_19_NO_3_, 285.1359). Spectroscopic data match those reported in the literature.^60^

### X-ray Crystallographic Data of Piperine (**7a**)

Piperine (**7a**) crystallized from a CHCl_3_ solution as colorless blocks. Crystallographic data were collected at 210 K on a Stoe Imaging Plate Diffraction System Stadivari, using Mo-*K*α radiation (λ = 0.71073 Å). Afterward, a spherical absorption correction (STOE LANA)^61^ and an extinction correction were performed. The structures were solved with SHELXT-2018/2 ^62^ using direct methods and refined against *F* ^2^ by full-matrix least-squares procedures with SHELXL-2018/3.^63^ The non-hydrogen atoms were refined anisotropically. For the visualization of the structure, the graphic program CSD Mercury was used.^64^

The crystallographic data can be obtained free of charge via https://www.ccdc.cam.ac.uk/structures/ or from The Cambridge Crystallographic Data Centre, 12 Union Road, Cambridge CB2 1EZ, UK; fax (+44) 1223-336-033; or via e-mail: deposit@ccdc.cam.ac; deposit number: CCDC 2501365.

Data for compound **7a**: C_17_H_19_NO_3_, M = 285.33 g/mol, 0.120 × 0.340 × 0.470 mm^3^, monoclinic, space group *P*2_1_/*n* (14), *a* = 8.7033(17) Å, *b* = 13.543(3) Å, *c* = 13.053(3) Å, α = 90°, β = 108.24(3)°, γ = 90°, *V* = 1461.3(6) Å^3^, *Z* = 4, *D*_c_ = 1.297 g/cm^3^, 27842 reflections measured (6.02° ≤ 2θ ≤ 55.00°), 3350 unique (*R*_int_ = 0.0770, *R*_sigma_ = 0.0295), which were used in all calculations. The final *R*_1_ was 0.0495 (*I* > 2σ (*I*)) and w*R*_2_ was 0.1296 (all data).

#### (2*E*,4*E*)-5-(Benzo­[*d*]­[1,3]­dioxol-5-yl)-*N*-isobutylpenta-2,4-dienamide (Piperlonguminine, **7b**)

Following the general procedure, piperic acid (**5**, 218 mg, 1.00 mmol) and 2-methylpropan-1-amine (**6b**, 169 μL, 1.70 mmol) were converted to **7b** (192 mg, 0.70 mmol, 70%): white powder; IR (ATR) ν̃ 3282 (m), 2960 (w), 1642 (s), 1606 (s), 1548 (s), 1503 (s), 1487 (s), 1444 (s), 1252 (s), 988 (s); ^1^H NMR (400 MHz, CDCl_3_): δ 7.35 (dd, *J* = 14.8, 10.6 Hz, 1H), 6.96–6.94 (m, 1H), 6.86 (dm, *J* = 8.0 Hz, 1H), 6.75 (d, *J* = 8.0 Hz, 1H), 6.75 (d, *J* = 15.6 Hz, 1H), 6.66 (dd, *J* = 15.5, 10.6 Hz, 1H), 5.96 (s, 2H), 5.95 (d, *J* = 14.8 Hz, 1H), 3.18 (t, *J* = 6.5 Hz, 2H), 1.82 (nonet, *J* = 6.5 Hz, 1H), 0.93 (d, *J* = 6.7 Hz, 6H); ^13^C­{^1^H} NMR (100 MHz, CDCl_3_): δ 166.3, 148.3, 148.3, 141.0, 138.9, 131.0, 124.8, 123.4, 122.7, 108.6, 105.8, 101.4, 47.1, 28.8, 20.3; HREIMS *m*/*z* 273.1355 [M^+^] (calcd for C_16_H_19_NO_3_, 273.1359). Spectroscopic data match those reported in the literature.^14^

#### (2*E*,4*E*)-5-(Benzo­[*d*]­[1,3]­dioxol-5-yl)-1-morpholinopenta-2,4-dien-1-one (**7c**)

Following the general procedure, piperic acid (**5**, 218 mg, 1.00 mmol) and morpholine (**6c**, 147 μL, 1.70 mmol) were converted to **7c** (237 mg, 0.82 mmol, 82%): white powder; IR (ATR) ν̃ 2985 (w), 2906 (w), 2871 (w), 1639 (s), 1591 (s), 1430 (s), 1259 (s), 1237 (s), 1115 (s), 992 (s); ^1^H NMR (400 MHz, CDCl_3_): δ 7.44 (dd, *J* = 14.6, 10.2 Hz, 1H), 6.97 (d, *J* = 1.7 Hz, 1H), 6.88 (dd, *J* = 8.1, 1.7 Hz, 1H), 6.77 (d, *J* = 15.5 Hz, 1H), 6.77 (d, *J* = 8.0 Hz, 1H), 6.71 (dd, *J* = 15.5, 10.2 Hz, 1H), 6.35 (d, *J* = 14.6 Hz, 1H), 5.96 (s, 2H), 3.71–3.56 (m, 8H); ^13^C {^1^H} NMR (100 MHz, CDCl_3_): δ 165.8, 148.4, 148.3, 143.5, 139.2, 130.9, 125.1, 122.8, 118.8, 108.6, 105.8, 101.4, 66.9, 46.2, 42.5; HRESIMS *m*/*z* 288.1240 [M + H]^+^ (calcd for C_16_H_18_NO_4_, 288.1230). Spectroscopic data match those reported in the literature.^65^

#### (2*E*,4*E*)-5-(Benzo­[*d*]­[1,3]­dioxol-5-yl)-1-(4-(trifluoromethyl)­piperidin-1-yl)­penta-2,4-dien-1-one (**7d**)

Following the general procedure, piperic acid (**5**, 1.00 mmol, 218 mg) and 4-(trifluoromethyl)­piperidine (**6d**, 322 mg, 1.70 mmol) were converted to **7d** (202 mg, 0.57 mmol, 57%): yellow oil; IR (ATR) ν̃ 2959 (w), 2902 (w), 2869 (w), 1637 (s), 1590 (s), 1489 (m), 1441 (s), 1333 (s), 1249 (s), 1127 (s), 1081 (s), 993 (s); ^1^H NMR (400 MHz, CDCl_3_): δ 7.40 (dd, *J* = 14.5, 9.5 Hz, 1H), 6.95 (d, *J* = 1.6 Hz, 1H), 6.86 (dd, *J* = 8.1, 1.6 Hz, 1H), 6.79–6.68 (m, 3H), 6.38 (d, *J* = 14.6 Hz, 1H), 5.94 (s, 2H), 4.77 (s­(br.), 1H), 4.12 (s­(br.), 1H), 3.03 (s­(br.), 1H), 2.60 (s­(br.), 1H), 2.34–2.18 (m, 1H), 1.92 (dm, *J* = 12.7 Hz, 2H), 1.51 (qd, *J* = 12.6, 4.5 Hz, 2H); ^13^C {^1^H} NMR (100 MHz, CDCl_3_): δ 165.6, 148.3, 148.3, 143.5, 139.0, 130.9, 127.0 (q, ^1^ *J*(^13^C–^19^F) = 278.1 Hz), 125.1, 122.7, 119.2, 108.5, 105.7, 101.4, 44.6 (br.), 41.0 (br.), 40.5 (q, ^3^ *J*(^13^C–^19^F) = 27.6 Hz), 25.3, 24.3; HREIMS *m*/*z* 353.1235 [M^+^] (calcd for C_18_H_18_F_3_NO_3_, 353.1233).

#### (2*E*,4*E*)-5-(Benzo­[*d*]­[1,3]­dioxol-5-yl)-*N*-benzylpenta-2,4-dienamide (**7e**)

Following the general procedure, piperic acid (**5**, 218 mg, 1.00 mmol) and benzylamine (**6e**, 185 μL, 1.70 mmol) were converted to **7e** (300 mg, 0.97 mmol, 97%): white powder; IR (ATR) ν̃ 3675 (m), 3281 (m), 2971 (s), 2916 (s), 1642 (m), 1607 (m), 1394 (m), 1252 (m), 1066 (s); ^1^H NMR (400 MHz, CDCl_3_): δ 7.41 (dd, *J* = 14.9, 10.8 Hz, 1H), 7.37–7.28 (m, 5H), 6.98 (d, *J* = 1.7 Hz, 1H), 6.90 (dd, *J* = 8.0, 1.7 Hz, 1H), 6.79 (d, *J* = 15.4 Hz, 1H), 6.78 (d, *J* = 8.0 Hz, 1H), 6.67 (dd, *J* = 15.4, 10.8 Hz, 1H), 5.98 (s, 2H), 5.92 (d, *J* = 14.9 Hz, 1H), 6.75 (tm­(br.), *J* = 5.5 Hz, 1H), 4.55 (d, *J* = 5.8 Hz, 2H); ^13^C {^1^H} NMR (100 MHz, CDCl_3_): δ 166.1, 148.4, 148.4, 141.7, 139.3, 138.4, 130.9, 128.9, 128.0, 127.7, 124.7, 122.9, 122.8, 108.6, 105.9, 101.5, 43.9; HREIMS *m*/*z* 307.1199 [M^+^] (calcd for C_19_H_17_NO_3_, 307.1203). Spectroscopic data match those reported in the literature.^66^

#### (2*E*,4*E*)-5-(Benzo­[*d*]­[1,3]­dioxol-5-yl)-*N*-(2-chlorobenzyl)­penta-2,4-dienamide (**7f**)

Following the general procedure, piperic acid (**5**, 218 mg, 1.00 mmol) and 2-chlorobenzylamine (**6f**, 205 μL, 1.70 mmol) were converted to **7f** (193 mg, 0.56 mmol, 56%): white powder; IR (ATR) ν̃ 3675 (w), 3268 (s), 3072 (w), 2988 (m), 2901 (m), 1646 (m), 1605 (s), 1548 (m), 1489 (m), 1357 (m), 1252 (s), 1039 (s), 993 (m); ^1^H NMR (400 MHz, DMSO-*d*_6_): δ 8.57 (t, *J* = 5.7 Hz, 1H), 7.44 (dm, *J* = 7.5 Hz, 1H), 7.36–7.25 (m, 4H), 7.21 (dd, *J* = 15.0, 10.2 Hz, 1H), 7.00 (dd, *J* = 8.0, 1.7 Hz, 1H), 6.96–6.83 (m, 3H), 6.20 (d, *J* = 15.0 Hz, 1H), 6.04 (s, 2H), 4.43 (d, *J* = 5.7 Hz, 2H); ^13^C­{^1^H} NMR (100 MHz, DMSO-*d*_6_): δ 165.4, 147.9, 147.8, 140.0, 138.3, 136.4, 132.1, 130.8, 129.1, 129.0, 128.7, 127.2, 125.2, 123.9, 122.7, 108.4, 105.7, 101.3, 40.2; HRESIMS *m*/*z* 342.0906 [M + H]^+^ (calcd for C_19_H_17_^35^ClNO_3_, 342.0891).

#### (2*E*,4*E*)-5-(Benzo­[*d*]­[1,3]­dioxol-5-yl)-*N*-(2-fluoro-4-(trifluoromethyl)­benzyl)­penta-2,4-dienamide (**7g**)

Following the general procedure, piperic acid (**5**, 218 mg, 1.00 mmol) and 2-fluoro-(4-trifluoromethyl)­benzylamine (**6g**, 328 mg, 1.70 mmol) were converted to **7g** (212 mg, 0.54 mmol, 54%): white powder; IR (ATR) ν̃ 3281 (w), 2901 (w), 1606 (m), 1333 (m), 1112 (s), 989 (s); ^1^H NMR (400 MHz, DMSO-*d*_6_): δ 8.66 (t, *J* = 5.9 Hz, 1H), 7.65 (d, *J* = 10.1 Hz, 1H), 7.61–7.50 (m, 2H), 7.26 (s, 1H), 7.21 (dd, *J* = 15.0, 10.1 Hz, 1H), 6.99 (d, *J* = 8.3 Hz, 1H), 6.96–6.86 (m, 3H), 6.16 (d, *J* = 15.0 Hz, 1H), 6.04 (s, 2H), 4.46 (d, *J* = 5.8 Hz, 2H); ^13^C­{^1^H} NMR (100 MHz, DMSO-*d*_6_): δ 165.5, 159.7 (d, ^1^ *J* = 247.6 Hz), 147.9, 147.8, 140.2, 138.4, 131.2 (d, *J* = 14.7 Hz), 130.7 (d, *J* = 16.5 Hz), 130.7, 125.1, 123.7, 122.8, 121.3 (m), 112.4 (dq, *J* = 25.1, 3.2 Hz), 108.4, 105.7, 101.3, 36.0 (m), two signals were not observed due to low intensity caused by ^13^C–^19^F-coupling; HRESIMS *m*/*z* 394.1050 [M + H]^+^ (calcd for C_20_H_16_F_4_NO_3_, 394.1061).

#### (2*E*,4*E*)-5-(Benzo­[*d*]­[1,3]­dioxol-5-yl)-*N*-(3-methoxybenzyl)­penta-2,4-dienamide (**7h**)

Following the general procedure, piperic acid (**5**, 218 mg, 1.00 mmol) and 3-methoxyphenylmethanamine (**6h**, 233 mg, 1.70 mmol) were converted to **7h** (303 mg, 0.90 mmol, 90%): white powder; IR (ATR) ν̃ 3268 (w), 2901 (w), 1638 (s), 1608 (s), 1586 (s), 1542 (s), 1486 (s), 1438 (s), 1253 (s), 985 (w); ^1^H NMR (400 MHz, CDCl_3_): δ 7.38 (dd, *J* = 14.9, 10.7 Hz, 1H), 7.25 (t, *J* = 7.9 Hz, 1H), 6.96 (d, *J* = 1.7 Hz, 1H), 6.89–6.74 (m, 6H), 6.66 (dd, *J* = 15.5, 10.7 Hz, 1H), 5.97 (s, 2H), 5.93 (d, *J* = 14.9 Hz, 1H), 5.93 (s­(br., 1H), 4.50 (d, *J* = 5.7 Hz, 2H), 3.79 (s, 3H); ^13^C­{^1^H} NMR (100 MHz, CDCl_3_): δ 166.2, 160.0, 148.4, 148.3, 141.7, 140.0, 139.3, 130.9, 129.9, 124.7, 122.9, 122.8, 120.2, 113.5, 113.1, 108.6, 105.9, 101.4, 55.4, 43.9; HRESIMS *m*/*z* 338.1386 [M + H]^+^ (calcd for C_20_H_20_NO_4_, 338.1387).

#### (2*E*,4*E*)-5-(Benzo­[*d*]­[1,3]­dioxol-5-yl)-*N*-(4-(dimethylamino)­benzyl)­penta-2,4-dienamide (**7i**)

Following the general procedure, piperic acid (**5**, 218 mg, 1.00 mmol) and 4-(dimethylamino)­benzylamine (**6i**, 379 mg, 1.70 mmol) were converted to **7i** (336 mg, 0.96 mmol, 96%): white powder; IR (ATR) ν̃ 327 (m), 2902 (w), 1642 (m), 1604 (s), 1523 (s), 1487 (s), 1438 (m), 1333 (m), 1243 (s), 19984 (s); ^1^H NMR (400 MHz, CDCl_3_): δ 7.47–7.35 (m, 2H), 7.25 (d, *J* = 8.7 Hz, 2H), 7.02 (d, *J* = 1.7 Hz, 1H), 6.94 (dd, *J* = 7.8, 1.7 Hz, 1H), 6.85–6.77 (m, 4H), 6.70 (dd, *J* = 15.4, 10.5 Hz, 1H), 6.25 (br t, *J* = 6.3 Hz, 1H), 6.04 (s, 2H), 6.01 (d, *J* = 15.4, 1H), 4.49 (d, *J* = 5.5 Hz, 2H), 3.01 (s, 6H); ^13^C­{^1^H} NMR (100 MHz, CDCl_3_): δ 166.5, 149.9, 148.4, 148.3, 141.5, 139.2, 130.9, 129.2, 126.5, 124.7, 122.9, 122.8, 113.2, 108.6, 105.9, 101.4, 43.6, 41.0; HRESIMS *m*/*z* 351.1702 [M + H]^+^ (calcd for C_21_H_23_N_2_O_3_, 351.1703).

#### (2*E*,4*E*)-5-(benzo­[*d*]­[1,3]­dioxol-5-yl)-*N*-(3,4,5-trimethoxybenzyl)­penta-2,4-dienamide (**7j**)

Following the general procedure, piperic acid (**5**, 218 mg, 1.00 mmol) and 3,4,5-trimethoxybenzylamine (**6j**, 290 μL, 1.70 mmol) were converted to **7j** (275 mg, 0.69 mmol, 69%): white powder; IR (ATR) ν̃ 3277 (w), 2937 (w), 1646 (m), 1591 (m), 1502 (s), 1488 (s), 1330 (m), 1238 (s), 1122 (s), 1035 (s), 995 (s); ^1^H NMR (400 MHz, DMSO-*d*_6_): δ 8.49 (t, *J* = 5.9 Hz, 1H), 7.26 (d, *J* = 1.7 Hz, 1H), 7.20 (dd, *J* = 15.0, 10.1 Hz, 1H), 6.99 (dd, *J* = 8.1, 1.7 Hz, 1H), 6.97–6.89 (m, 2H), 6.87 (d, *J* = 15.5 Hz, 1H), 6.60 (s, 2H), 6.16 (d, *J* = 15.0 Hz, 1H), 6.04 (s, 2H), 4.30 (d, *J* = 5.8 Hz, 2H), 3.75 (s, 6H), 3.63 (s, 3H); ^13^C­{^1^H} NMR (100 MHz, DMSO-*d*_6_): δ 165.2, 152.8, 147.9, 147.7, 139.7, 138.1, 136.4, 135.1, 130.9, 125.2, 124.3, 122.7, 108.4, 105.6, 104.8, 101.3, 60.0, 55.8, 42.5; HRESIMS *m*/*z* 398.1619 [M + H]^+^ (calcd for C_22_H_24_NO_6_, 398.1604).

#### (2*E*,4*E*)-5-(Benzo­[*d*]­[1,3]­dioxol-5-yl)-*N*-(3,4-dimethoxyphenethyl)­penta-2,4-dienamide (**7k**)

Following the general procedure, piperic acid **5** (218 mg, 1.00 mmol) and 3,4-dimethoxyphenylethylamine (**6k**, 287 μL, 1.70 mmol) were converted to **7k** (178 mg, 0.47 mmol, 47%): white powder; IR (ATR) ν̃ 3675 (w), 3285 (w), 2961 (m), 2918 (m), 1647 (w), 1604 (m), 1512 (m), 1252 (s), 1037 (s), 996 (m); ^1^H NMR (400 MHz, DMSO-*d*_6_): δ 8.09 (t, *J* = 5.6 Hz, 1H), 7.25 (d, *J* = 1.7 Hz, 1H), 7.15 (dd, *J* = 15.0, 10.1 Hz, 1H), 6.98 (dd, *J* = 8.1, 1.7 Hz, 1H), 6.94–6.79 (m, 5H), 6.71 (dd, *J* = 8.1, 2.0 Hz, 1H), 6.08 (d, *J* = 15.0 Hz, 1H), 6.04 (s, 2H), 3.73 (s, 3H), 3.71 (s, 3H), 3.35 (dt, *J* = 7.4, 5.6 Hz, 2H), 2.68 (t, *J* = 7.4 Hz, 2H); ^13^C {^1^H} NMR (100 MHz, DMSO-*d*_6_): δ 165.1, 148.6, 147.9, 147.7, 147.2, 139.2, 137.8, 132.0, 130.9, 125.3, 124.6, 122.6, 120.4, 112.5, 111.9, 108.4, 105.6, 101.3, 55.5, 55.4, 40.5, 34.7; HRESIMS *m*/*z* 382.1666 [M + H]^+^ (calcd for C_22_H_24_NO_5_, 382.1649). The title compound has previously been reported in the literature,^27,67^ but apart from ^1^H NMR-data in CDCl_3_^68^ no other spectroscopical data have been reported.

#### (2*E*,4*E*)-5-(Benzo­[*d*]­[1,3]­dioxol-5-yl)-*N*-(4-(4-(4-fluorophenyl)­piperidin-1-yl)­benzyl)­penta-2,4-dienamide (**7l**)

Following the general procedure, piperic acid **5** (218 mg, 1.00 mmol) and (4-(4-(4-fluorophenyl)­piperidin-1-yl)­phenyl)­methanamine^55^ (**6l**, 483 mg, 1.70 mmol) were converted to **7l** (327 mg, 0.67 mmol, 67%): beige powder; IR (ATR) ν̃ 3281 (s), 2918 (m), 1645 (s), 1614 (m), 1604 (m), 1547 (s), 1510 (s), 1490 (s), 1448 (s), 1254 (s), 1039 (s), 987 (m); ^1^H NMR (400 MHz, CDCl_3_): δ 7.39 (dd, *J* = 14.9, 10.7 Hz, 1H), 7.23–7.17 (m, 4H), 7.02–6.92 (m, 5H), 6.89 (dd, *J* = 8.1, 1.7 Hz, 1H), 6.77 (d, *J* = 15.4 Hz, 1H), 6.77 (d, *J* = 8.1 Hz, 1H), 6.67 (dd, *J* = 15.5, 10.7 Hz, 1H), 5.97 (s, 2H), 5.91 (d, *J* = 14.9 Hz, 1H), 5.74 (t­(br), *J* = 5.6 Hz, 1H), 4.46 (d, *J* = 5.6 Hz, 2H), 3.78 (dm, *J* = 12.5 Hz, 2H), 2.80 (td, *J* = 12.5, 2.6 Hz, 2H), 2.63 (tt, *J* = 12.0, 3.8 Hz, 1H), 1.93 (dm, *J* = 12.5 Hz, 2H), 1.84 (qd, *J* = 12.5, 3.8 Hz, 2H); ^13^C­{^1^H} NMR (100 MHz, CDCl_3_): δ 166.0, 161.5 (d, ^1^ *J*(^13^C–^19^F) = 244.3 Hz), 151.4, 148.4, 148.4, 141.8 (d, *J* = 3.1 Hz), 141.4, 139.1, 131.0, 129.2, 129.1, 128.3 (d, *J* = 7.8 Hz), 124.8, 123.1, 122.8, 116.9, 115.4 (d, *J* = 21.1 Hz), 108.6, 105.9, 101.4, 50.6, 43.5, 41.9, 33.5; HREIMS *m*/*z* 484.2151 [M^+^] (calcd for C_30_H_29_FN_2_O_3_, 484.2157).

### Biological Assays

#### Antimicrobial Assay

The antimicrobial activity was determined according to our previously reported procedures.^69–71^ Briefly, the Minimum Inhibitory Concentrations (MICs) in μg/mL of compounds **5**, **7a**–**7l** were determined by serial dilution assay against 12 strains including five fungal species (*Schizosaccharomyces pombe* DSM-70572, *Wickerhamomyces anomalus* DSM-6766, *M. hiemalis* DSM-2656, *C. albicans* DSM-1665, and *R. glutinis* DSM-10134), three Gram-positive bacteria (*Bacillus subtilis* DSM-10, *Mycolicibacterium smegmatis* ATCC 700084, and *S. aureus* DSM-346), and four Gram-negative bacteria (*Acinetobacter baumannii* DSM30008, *Echerichia coli* DSM1116, *P. aeruginosa* DSM19882, and *Chromobacterium violaceum* DSM30191). The assays were carried out in 96-well microtiter plates in YM6.3 media (10 g/L malt extract, 4 g/L glucose, 4 g/L yeast extract, pH 6.3) for filamentous fungi and yeast and Müller-Hinton broth for bacteria. Nystatin, ciprofloxacin, gentamycin, oxytetracycline, and kanamycin were used as references.

#### Cytotoxicity Assay

The cytotoxicity of compounds **5**, **7a**–**7l** against cell lines (murine fibroblasts L929, human cervix carcinoma KB-3-1, breast cancer MCF-7, epidermoid carcinoma A431, human lung carcinoma A549, and human prostate cancer PC-3) was evaluated using the MTT (2-(4,5-dimethylthiazol-2-yl)-2,5-diphenyltetrazolium bromide) method in 96-well microplates.^32,68,70^ The cell lines were cultured in DMEM (Gibco). Briefly, 60 μL aliquots of serial dilutions from an initial stock of 1 mg/mL in MeOH of the test compounds were added to 120 μL aliquots of a cell suspension (5 × 104 cells/mL) in 96-well microplates. After 5 days incubation, an MTT assay was performed, and the absorbance measured at 590 nm using a multimode plate reader (Infinite M200 Pro, Tecan). The concentration at which the growth of cells was inhibited to 50% of the control (IC_50_) was obtained from the dose–response curves. Epothilone B was used as the positive control.

#### ADME, Drug-Likeness

The ADME (Absorption, Distribution, Metabolism, and Excretion) properties and drug-likeness of compounds **5**, **7a**–**7l** were evaluated using the computational tool QikProp from Schrödinger Maestro-2023-2 as described previously.^54^

## Supplementary Material



## Data Availability

Primary HRMS, IR and NMR-FID files for compounds **1b**, **2**, **3**, **4**, **5**, **7a**, **7b**, **7c**, **7d**, **7e**, **7f**, **7g**, **7h**, **7i**, **7j**, **7k**, **7l** (ZIP) are available via the Zenodo-data repository at 10.5281/zenodo.18630878.

## Notes

Copies of ^1^H, ^13^C­{^1^H}, 2D-NMR spectra of all compounds; crystallographic details and refinement data for **7a**, tabular summary of antimicrobial assays (PDF). X-ray crystallographic data (CIF) for **7a** (PDF)
