# Supplementary material for: Total Synthesis of Piperine and Derivatives: Antimicrobial and Cytotoxic Activities
Source: ACS Omega. 2026 Jun 23;11(26):39218–27. doi: 10.1021/acsomega.6c03524 (PMC13347353; doi:10.1021/acsomega.6c03524)
Supplement: Supplementary file 1 [file ao6c03524_si_001.pdf]

Supporting Information

Total Synthesis of Piperine and Derivatives: Antimicrobial and Cytotoxic Activities

Jenifer Reine Ngnouzouba Kuete,[a,b] Vaderament-Alexe Nchiozem-Ngnitedem,[b]* Eric Sperlich,[b] Birthe Sandargo,[c] Léon Azefack Tapondjou,[a] Rémy Bertrand Teponno,[a]* and Bernd Schmidt.[b]

[a]Department of Chemistry, Faculty of Science, University of Dschang, P.O. Box 67, Dschang, Cameroon

[b]Institut für Chemie, University of Potsdam, D-14476 Potsdam-Golm, Germany

[c]Department Microbial Drugs, Helmholtz Centre for Infection Research (*HZI*), 38124 Braunschweig, Germany

*e-mail: nchiozem-ngnitedem@uni-potsdam.de (Vaderament-Alexe Nchiozem-Ngnitedem); remyteponno@gmail.com (Rémy Bertrand Teponno)

A Single crystal X-ray structure analyses of piperine (7a)

1 General details of X-ray structure analysis

The crystal structure was determined by single crystal structure analysis. Suitable single crystals were selected using a Leica M205C light microscope and separated with oil. X-ray crystal structure analysis was performed on a Stadivari diffractometer (Stoe) with monochromated Mo-$K\alpha$ radiation (λ = 0.71073 Å). The data correction was performed using the program X-Area.[1] The structure was solved by direct methods and refined against F^2 on all data by full-matrix least-squares using the SHELX suite of programs.[2,3] All non-hydrogen atoms were refined anisotropically; the hydrogen atoms were placed on calculated positions. **Table S1** was created using FinalCif.[4] The crystal structure was visualized with Mercury.[5] The data (**7a**: CCDC 2501365) can be obtained free of charge from the Cambridge Crystallographic Data Centre, http://www.ccdc.cam.ac.uk. A single crystal structure analysis of piperine isolated from black pepper had previously been published.[6]

References

(1) STOE & Cie GmbH (2018) X-Area. software package for collecting single-crystal data on STOE area-detector diffractometers, for image processing, for the correction and scaling of reflection intensities and for outlier rejection. STOE & Cie GmbH, Darmstadt.

(2) Sheldrick, G. Crystal structure refinement with SHELXL. *Acta Cryst. C* **2015**, *C71*, 3-8.

(3) Sheldrick, G. A short history of SHELX. *Acta Cryst. A* **2008**, *A64*, 112-122.

(4) Kratzert, D. FinalCif, V123.

(5) Macrae, C. F.; Sovago, I.; Cottrell, S. J.; Galek, P. T. A.; McCabe, P.; Pidcock, E.; Platings, M.; Shields, G. P.; Stevens, J. S.; Towler, M.; Wood, P. A. Mercury 4.0: from visualization to analysis, design and prediction. *J. Appl. Cryst.*, **2020**, *53*, 226-235.

(6) Grynpas, M.; Lindley, P. F. The crystal and molecular structure of 1-piperoylpiperidine. *Acta Cryst. B* **1975**, *31*, 2663-2667.

2 Crystallographic Data:

Table S1. Crystal data and details of structure refinement for piperine (**7a**).

Compound	Piperine (**7a**)
CCDC number	2501365
Empirical formula	$C_{17}H_{19}NO_3$
Formula weight	285.33
Temperature [K]	210
Crystal system	monoclinic
Space group (number)	$P2_1/n$ (14)
a [Å]	8.7033(17)
b [Å]	13.543(3)
c [Å]	13.053(3)
α [°]	90
β [°]	108.24(3)
γ [°]	90
Volume [Å^3]	1461.3(6)
Z	4
ρ_{calc} [gcm^{-3}]	1.297
μ [mm^{-1}]	0.089
F(000)	608
Crystal size [mm^3]	0.120×0.340×0.470
Crystal color	colorless
Crystal shape	block
Radiation	Mo K_α (λ=0.71073 Å)
2θ range [°]	6.02 to 55.00 (0.77 Å)
Index ranges	$-11 \leq h \leq 11$<br>$-17 \leq k \leq 17$<br>$-16 \leq l \leq 16$
Reflections collected	27842
Independent reflections	3350<br>R_{int} = 0.0770<br>R_{sigma} = 0.0295
Completeness to θ = 25.242°	99.5 %
Data / Restraints / Parameters	3350 / 0 / 191
Goodness-of-fit on F^2	1.049
Final R indexes [$I \geq 2\sigma(I)$]	R_1 = 0.0495<br>wR_2 = 0.1296
Final R indexes [all data]	R_1 = 0.0627<br>wR_2 = 0.1413
Largest peak/hole [eÅ^{-3}]	0.31/−0.27

3 Visualization of the crystal structure and molecular structure for compound 7a

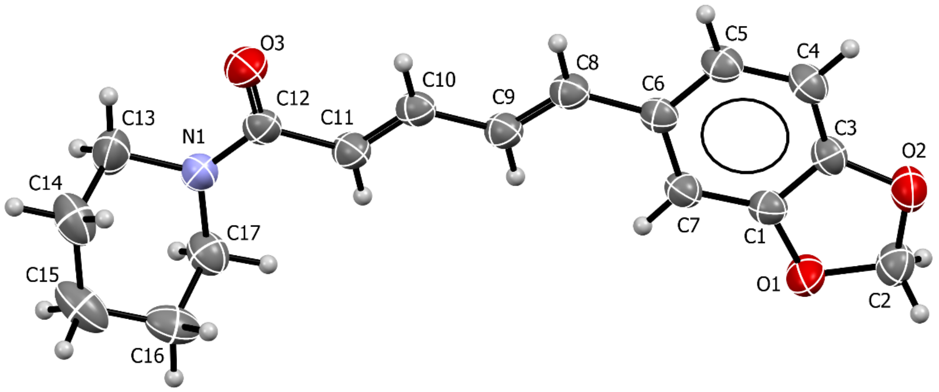

Figure S1: Molecular structure with atom labeling of compound **7a**. Displacement ellipsoids are shown at the 50% probability level.

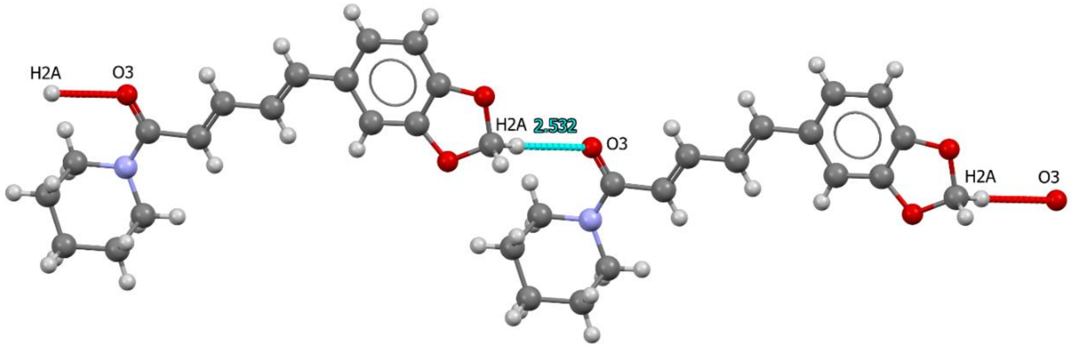

Figure S2: C-H···O hydrogen bonds in **7a** (blue dashed lines).

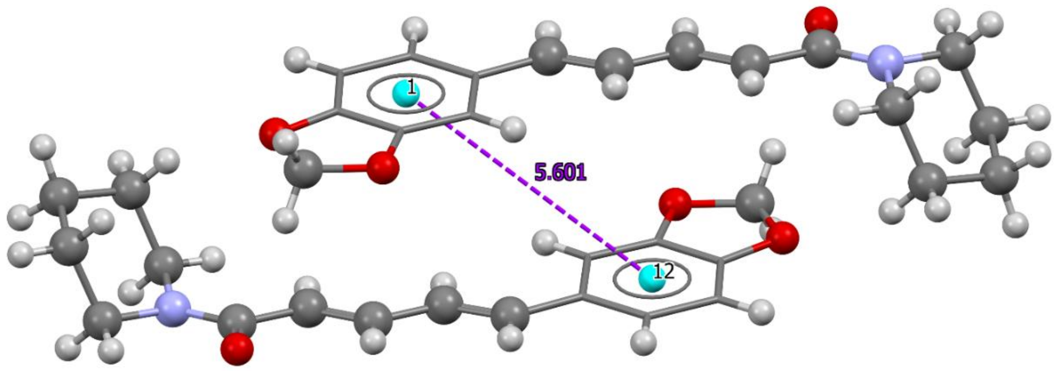

Figure S3: Strong parallel-displaced stacking interactions (purple dotted lines) between the molecules in **7a**.

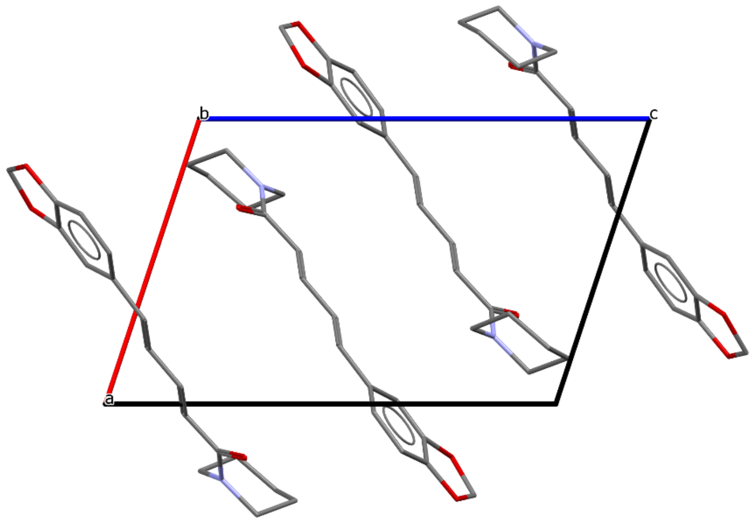

Figure S4: Cell view of compound **7a** looking along the crystallographic b axis.

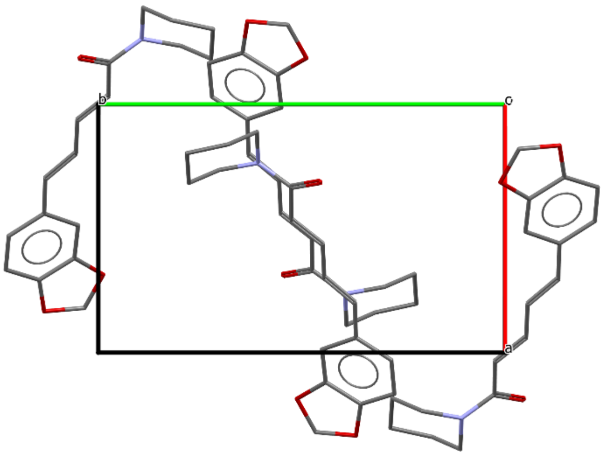

Figure S5: Cell view of compound **7a** looking along the crystallographic c axis.

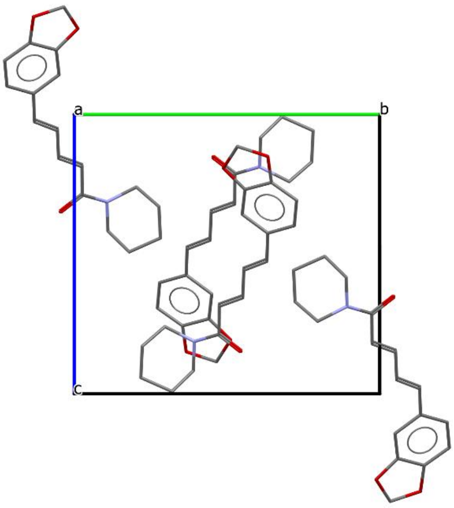

Figure S6: Cell view of compound **7a** looking along the crystallographic a axis.

Figure S7: ^{1}H NMR (400 MHz, $CDCl_3$) of **1b**.

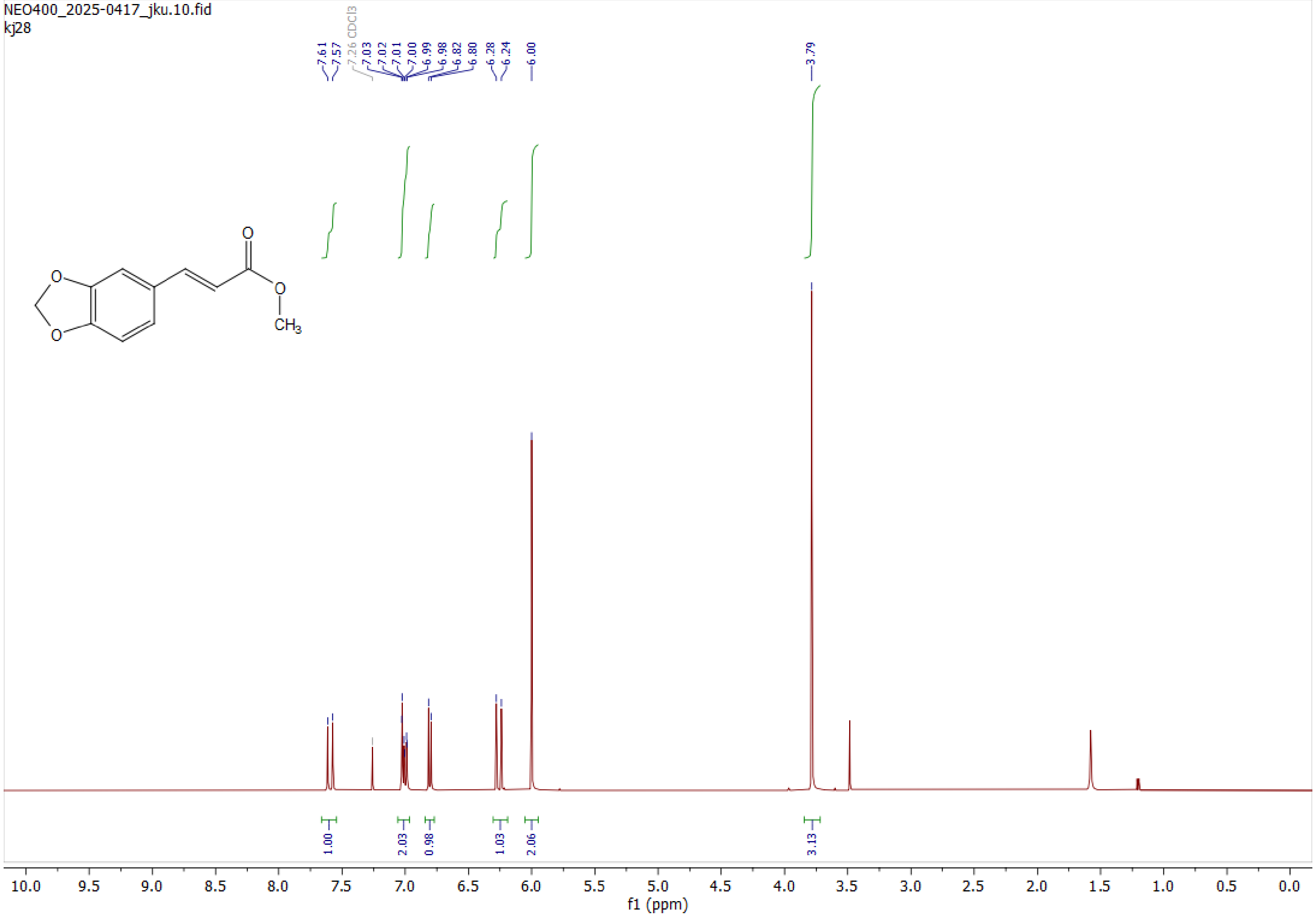

Figure S8: $^{13}C\{^{1}H\}$ NMR (100 MHz, $CDCl_3$) of **1b**.

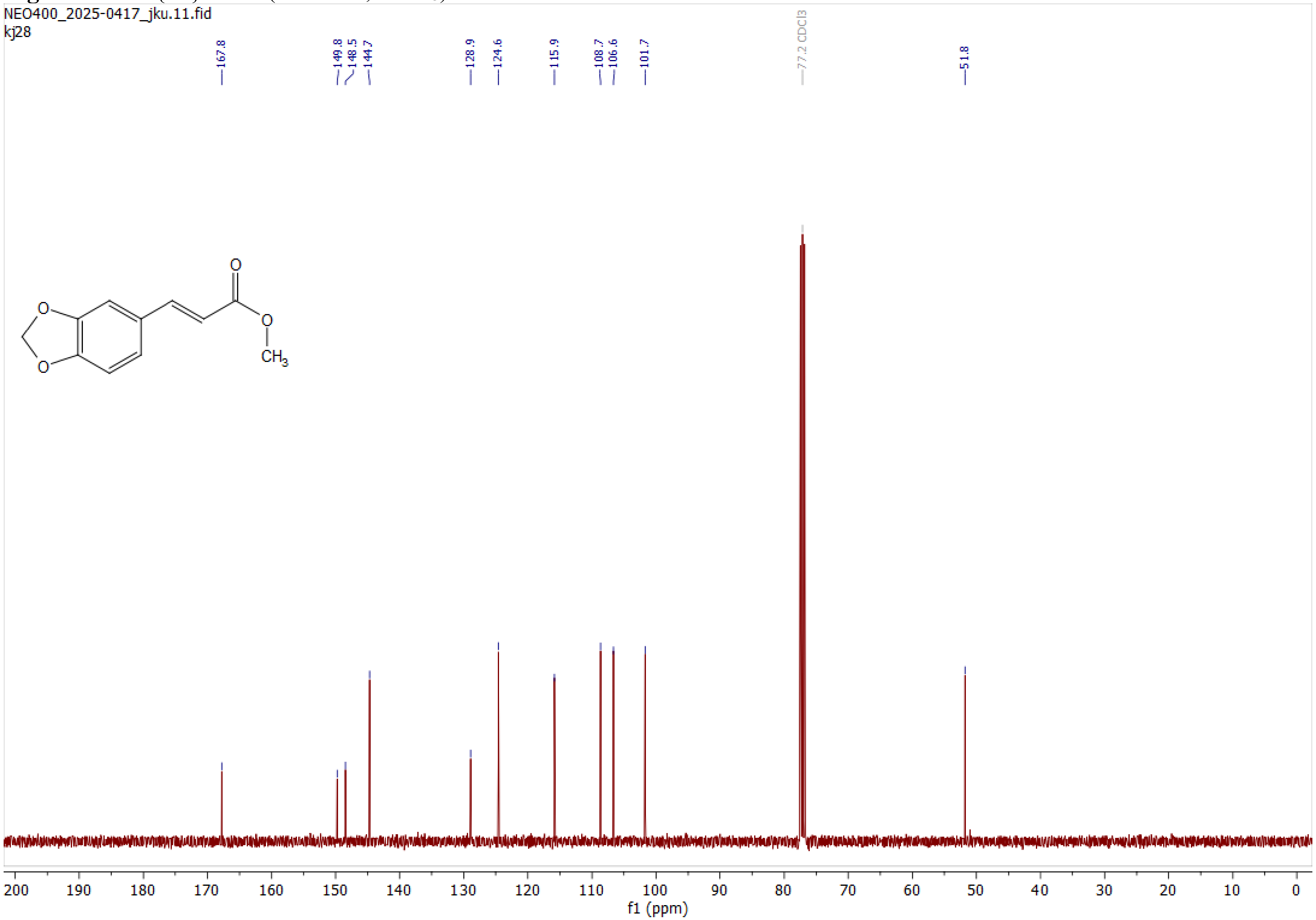

Figure S9: ^{1}H NMR (400 MHz, $CDCl_3$) of **2**.

NEO400_2025-0407_jku.60.fid
kj29

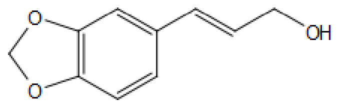

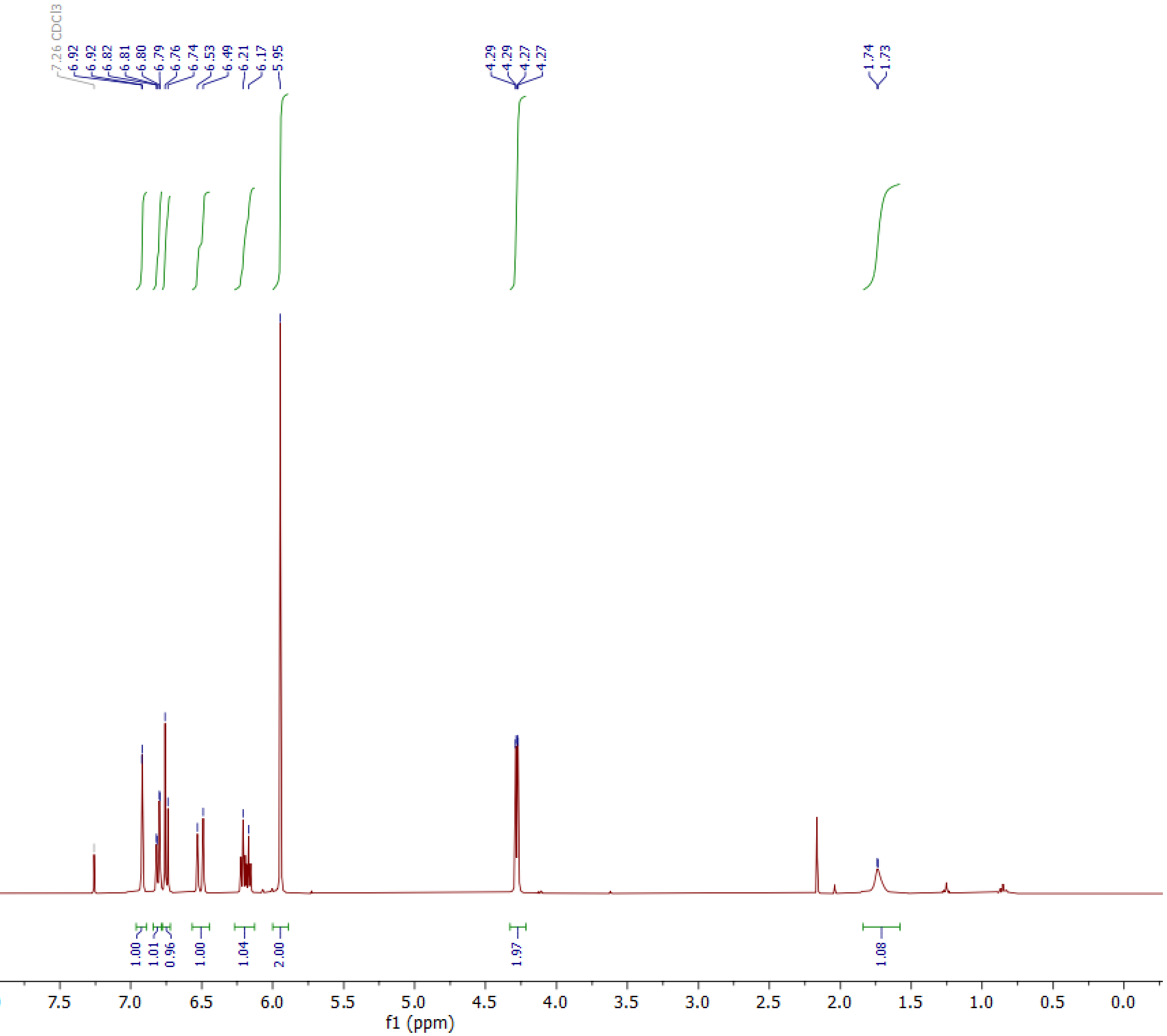

Figure S10: $^{13}C\{^1H\}$ NMR (100 MHz, $CDCl_3$) of **2**.

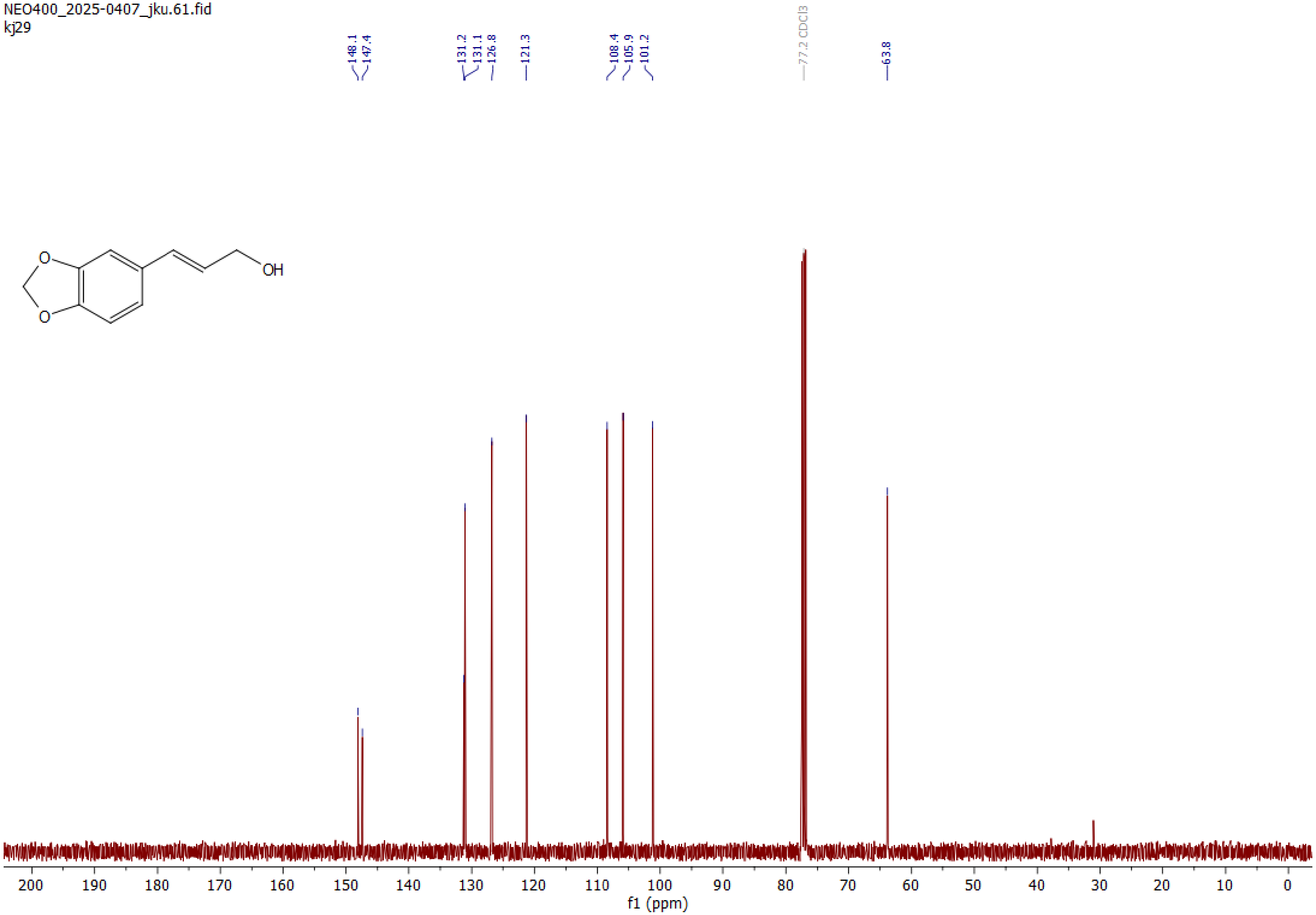

Figure S11: ^{1}H NMR (400 MHz, $CDCl_3$) of **3**.

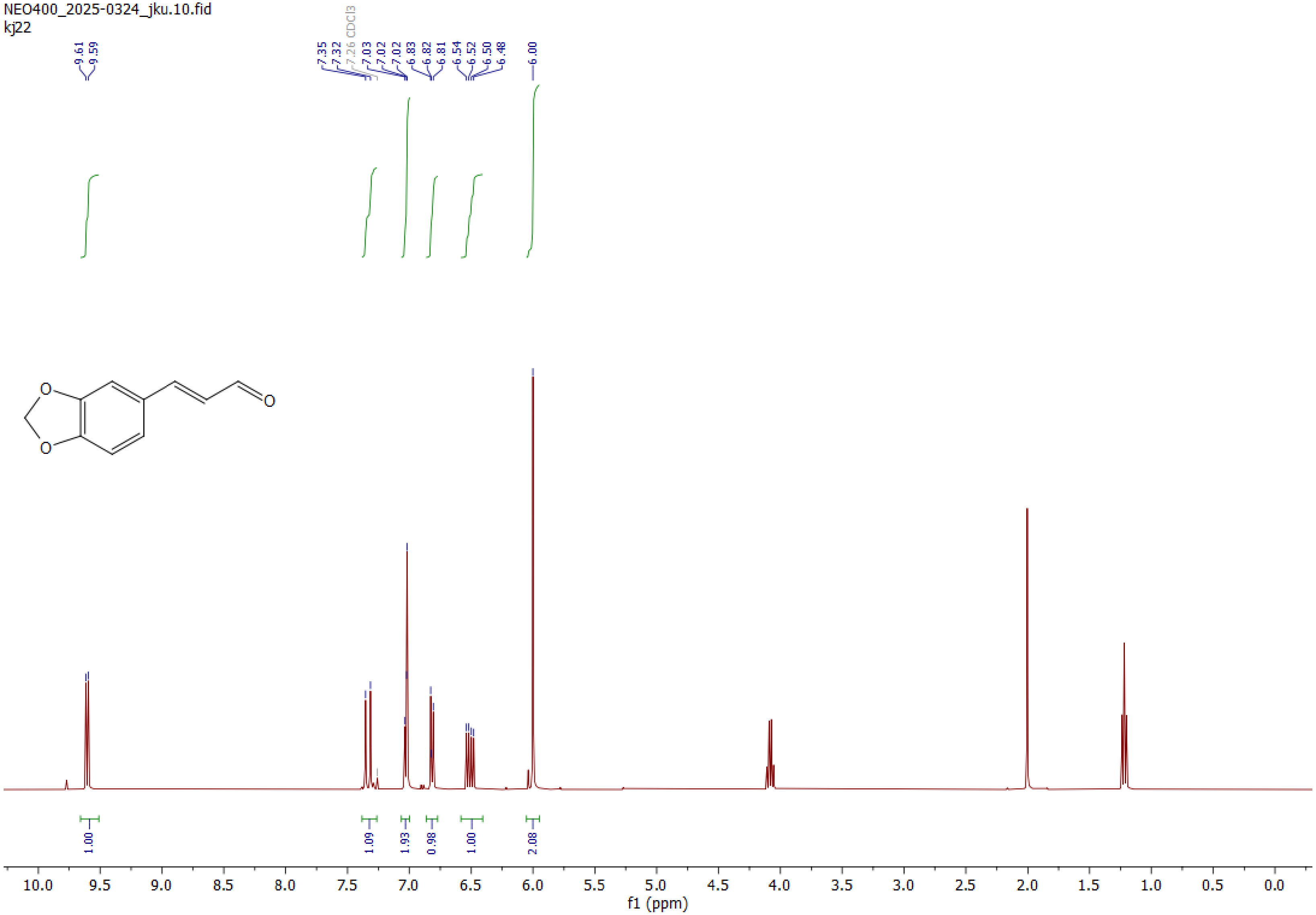

Figure S12: $^{13}C\{^{1}H\}$ NMR (100 MHz, $CDCl_3$) of **3**.

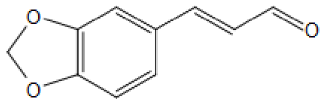

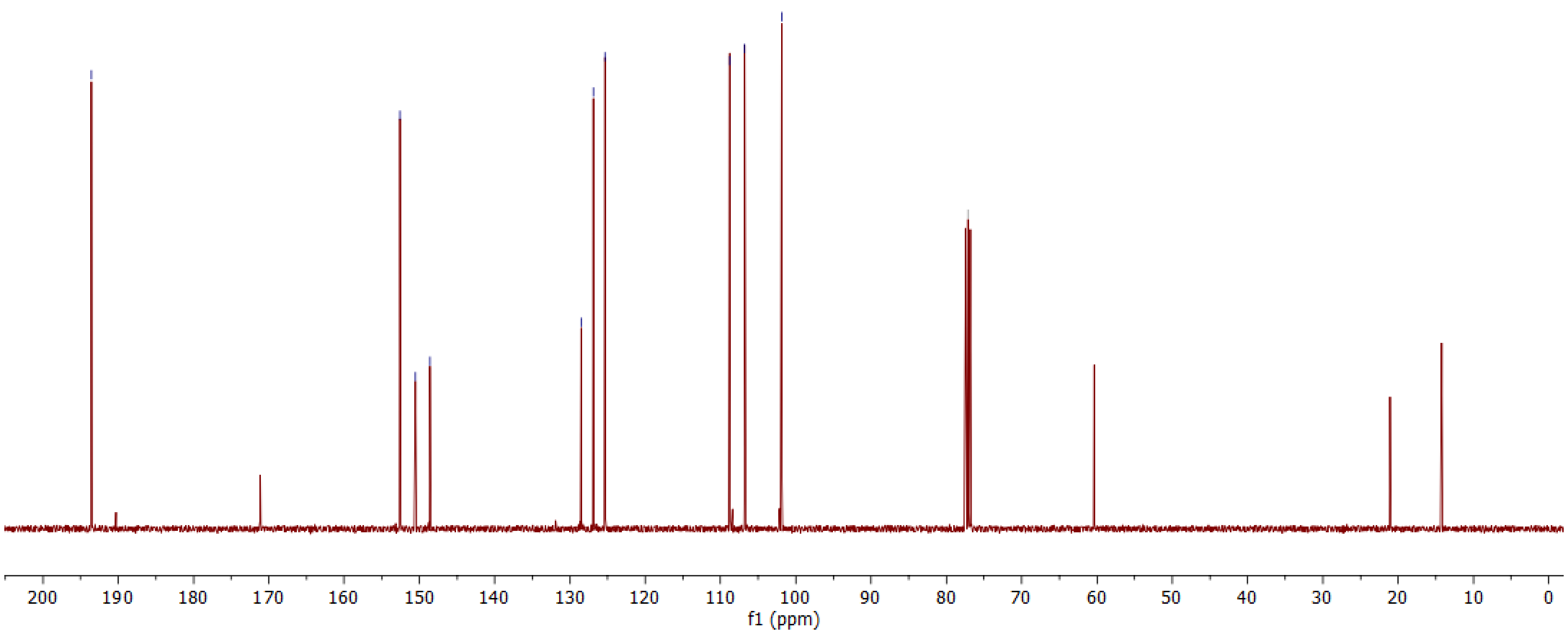

Figure S13: ^{1}H NMR (400 MHz, $CDCl_3$) of **4**.

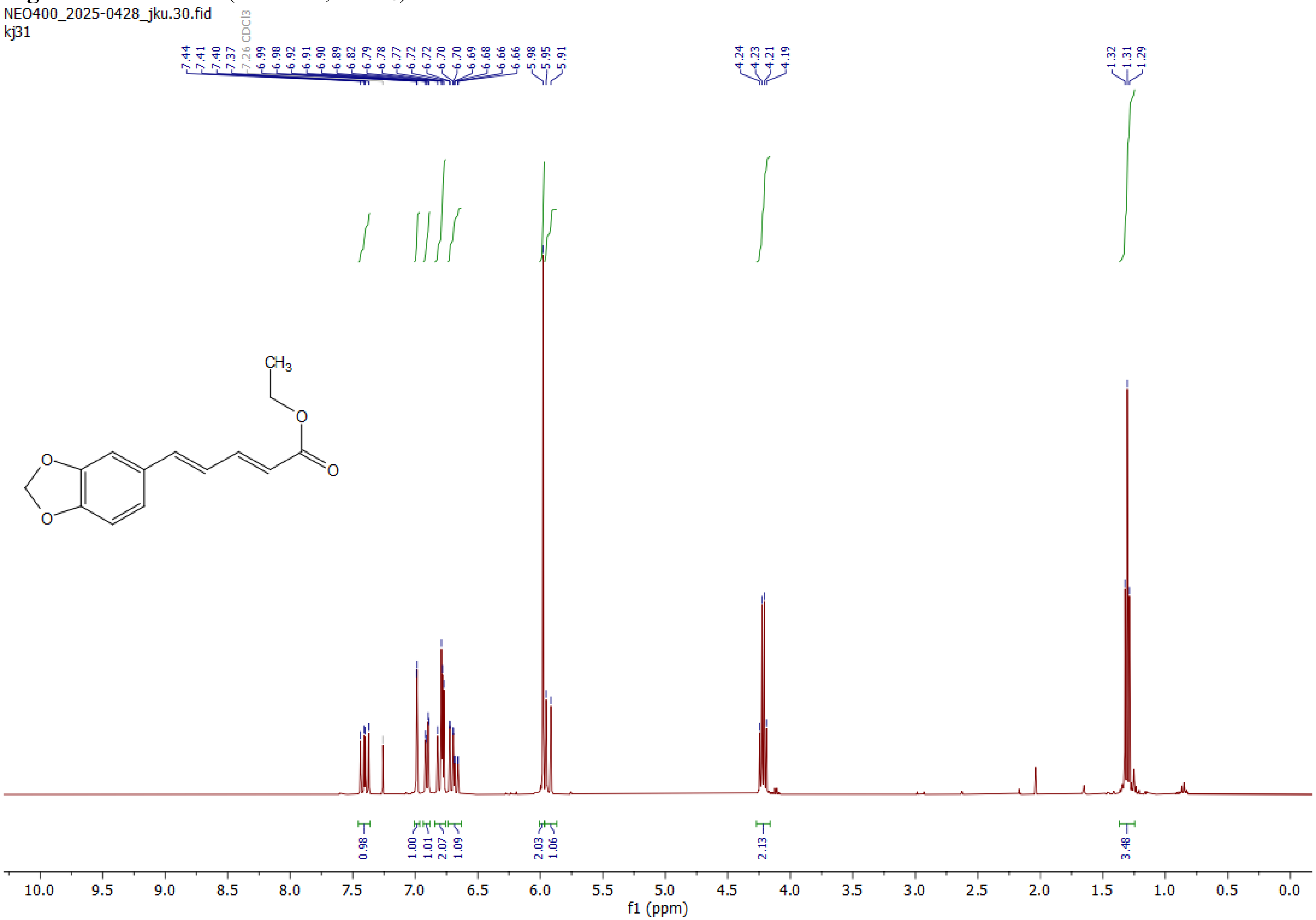

Figure S14: $^{13}C\{^{1}H\}$ NMR (100 MHz, $CDCl_3$) of **4**.

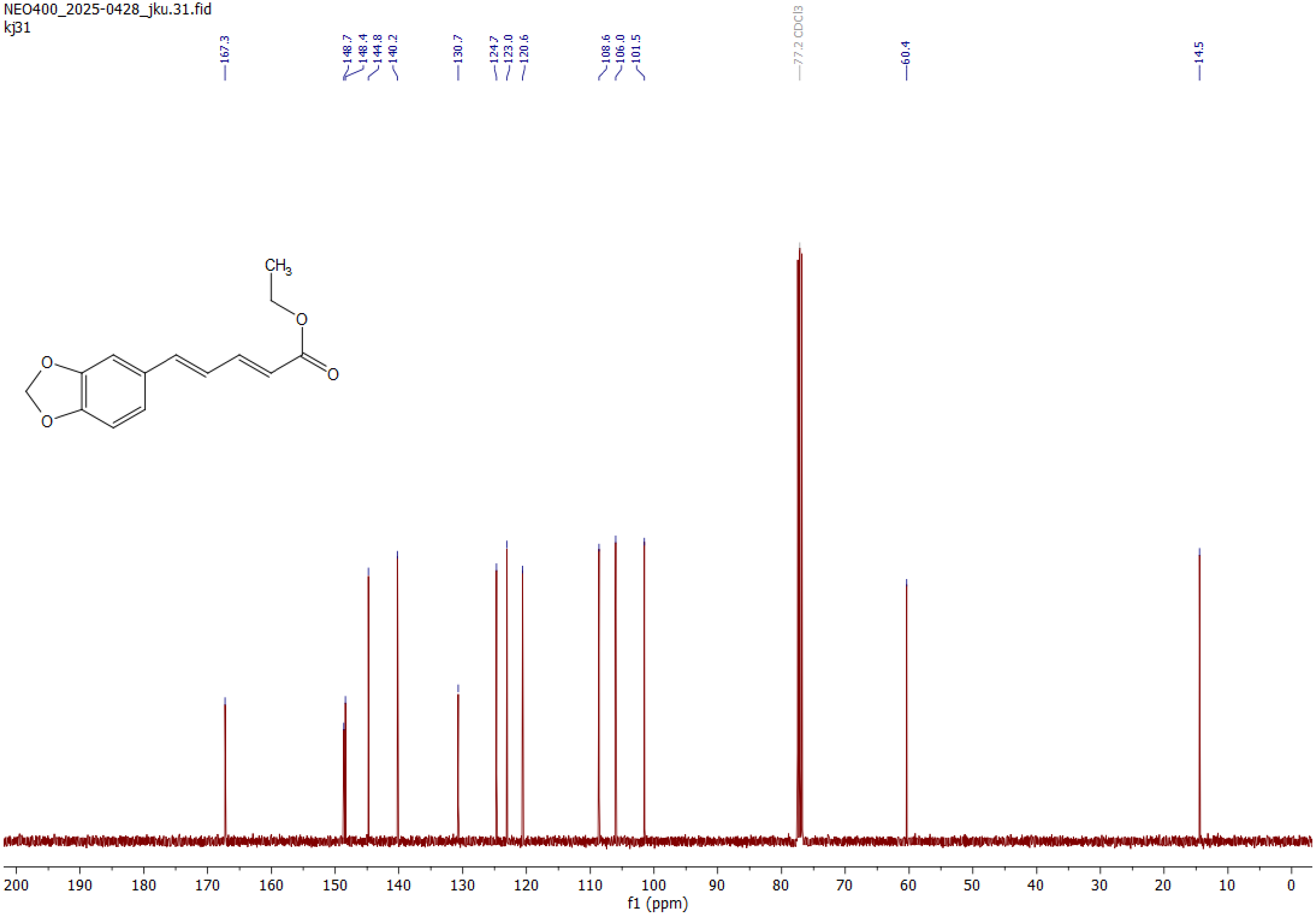

Figure S15: H,H-COSY (400 MHz, $CDCl_3$) of **4**.

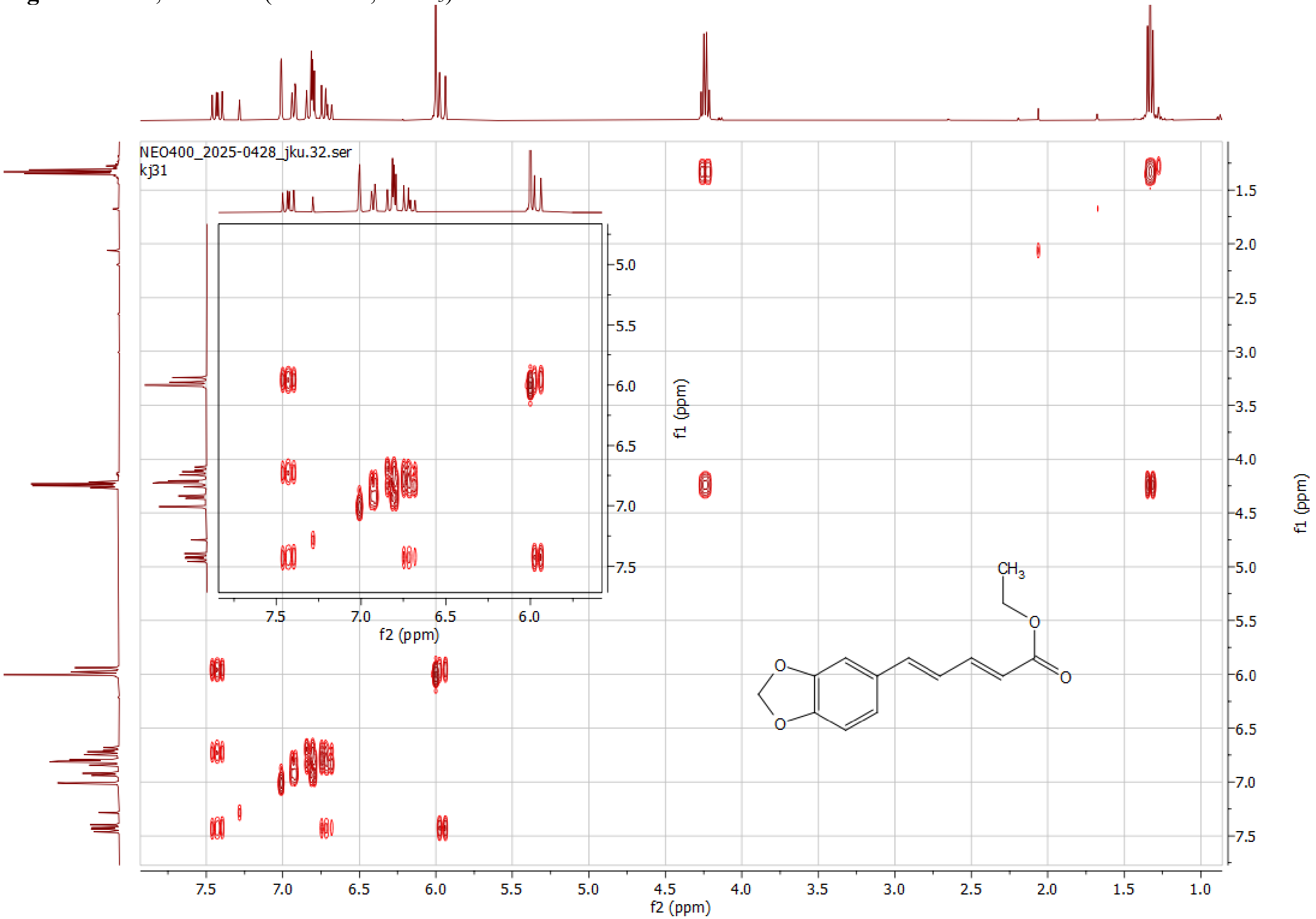

Figure S16: HSQC (400/100 MHz, $CDCl_3$) of **4**.

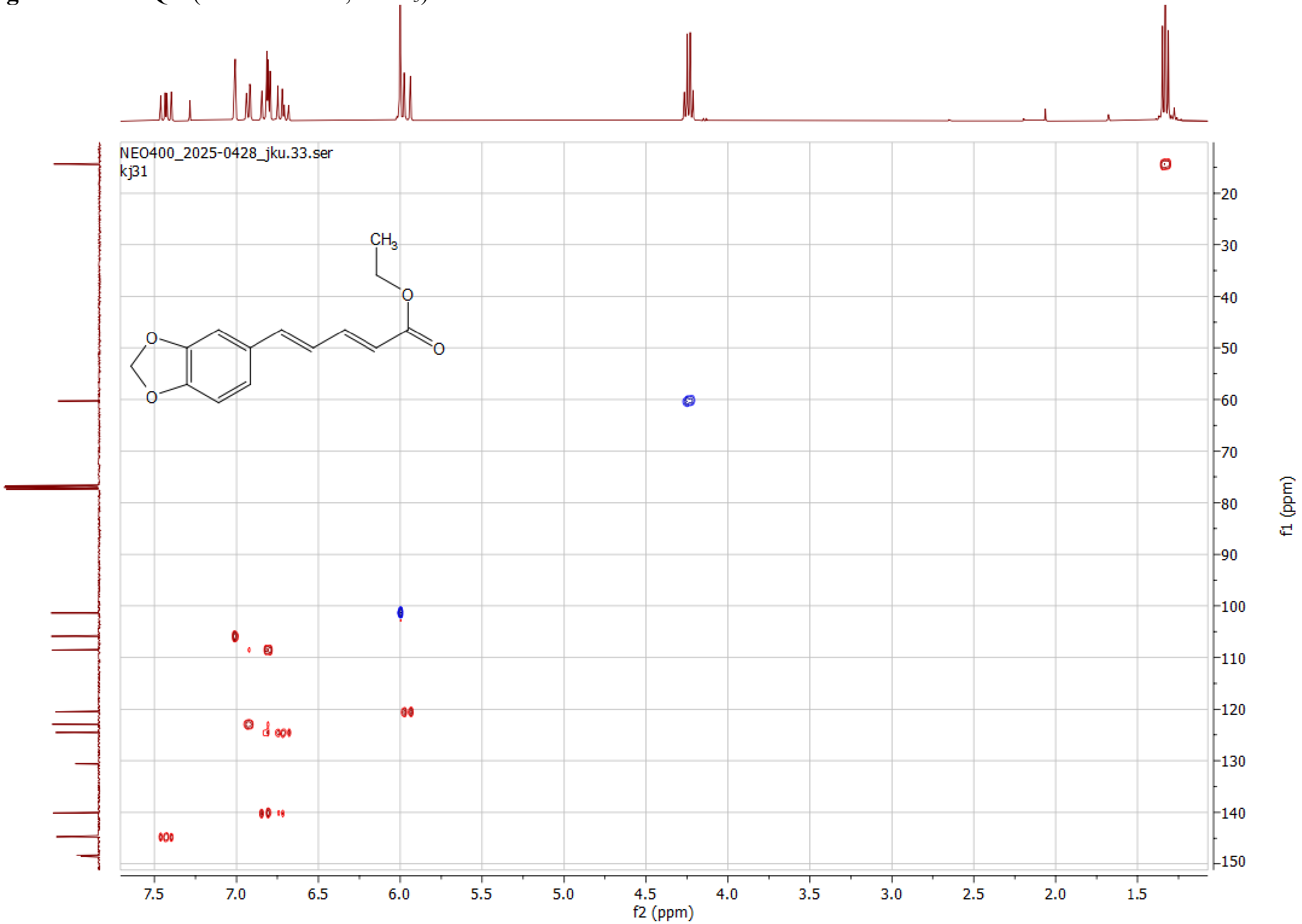

Figure S17: HMBC (400/100 MHz, $CDCl_3$) of **4**.

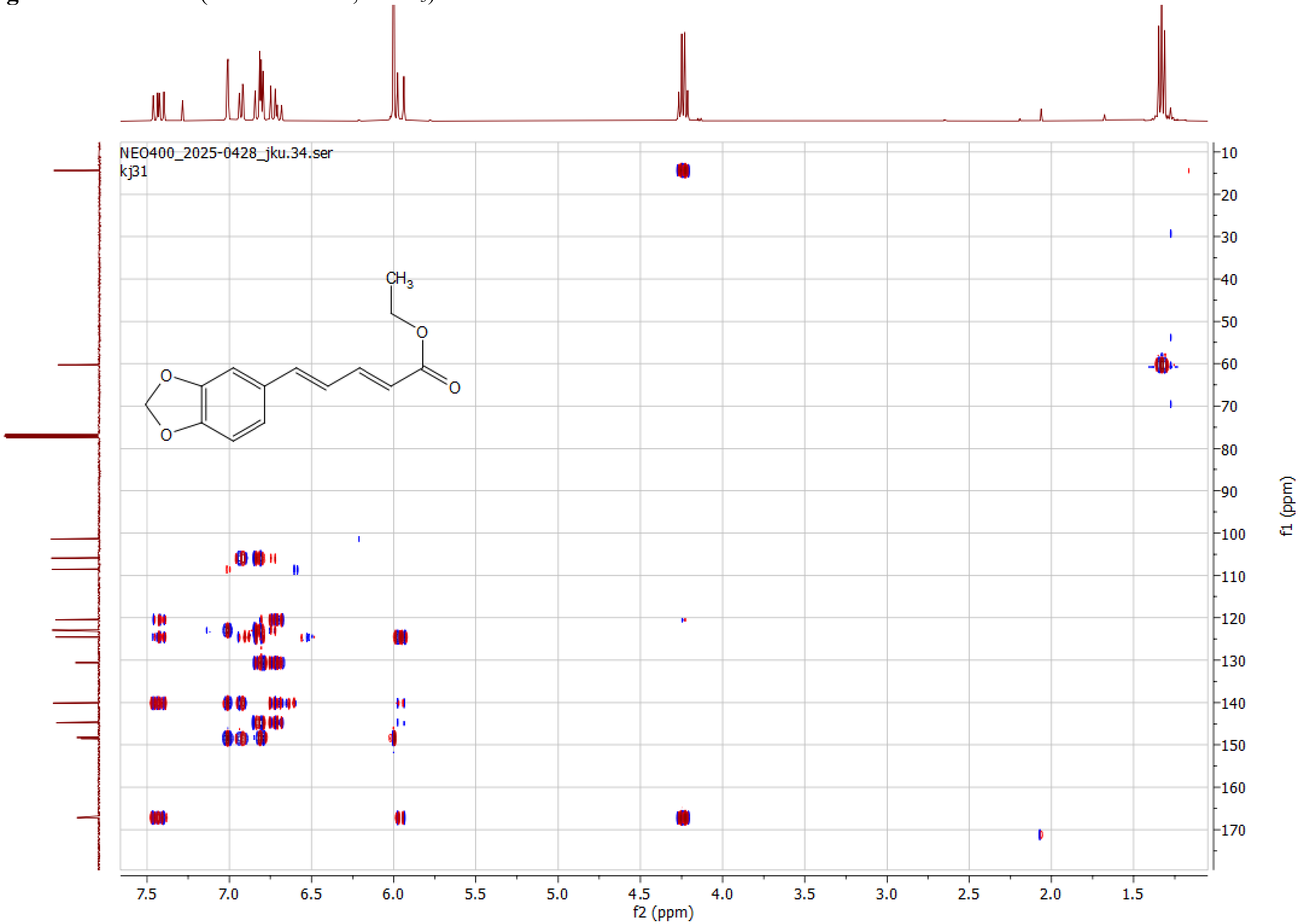

Figure S18: ^{1}H NMR (400 MHz, DMSO-d_6) of **5**.

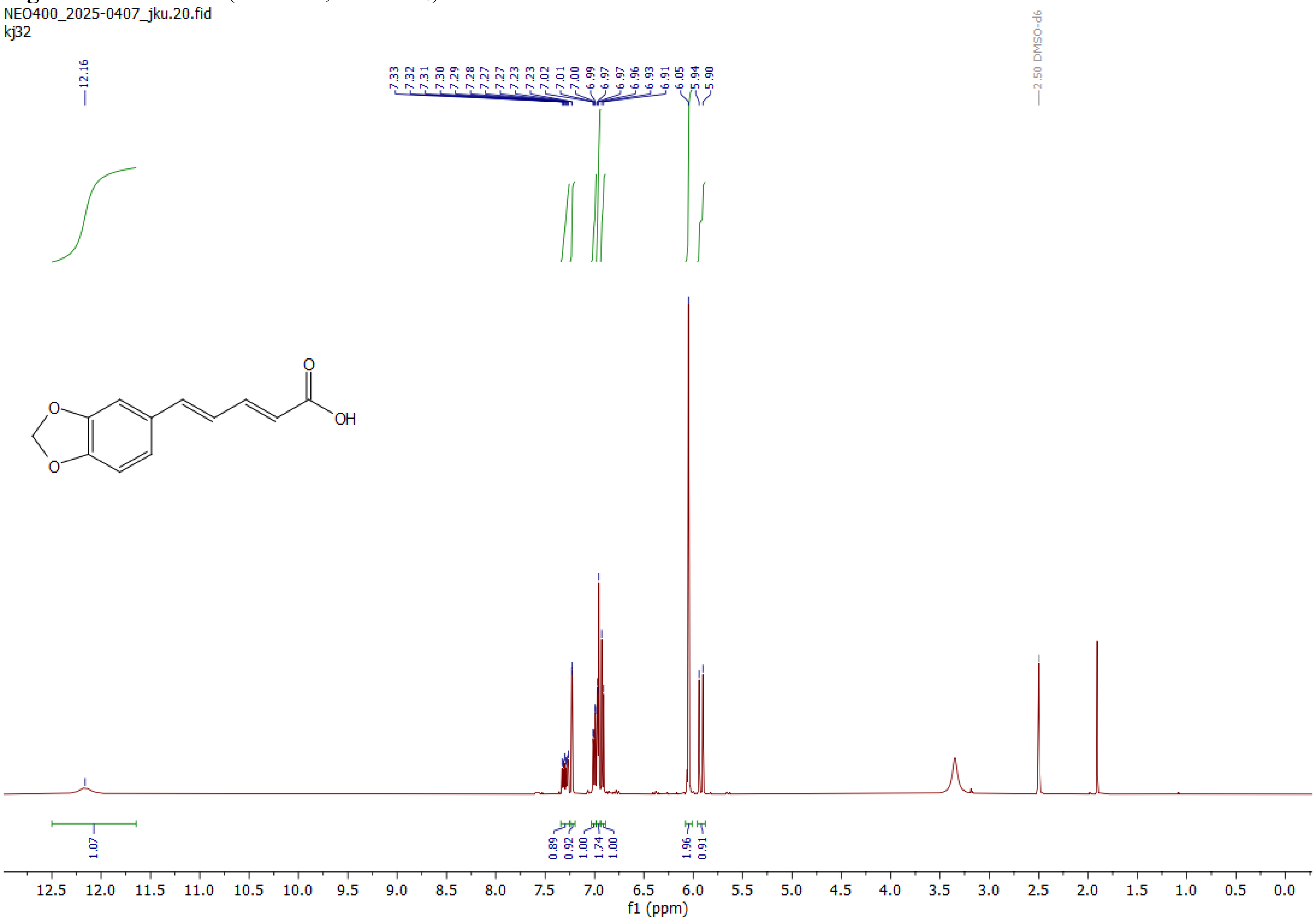

Figure S19: $^{13}C\{^{1}H\}$ NMR (100 MHz, DMSO-d_6) of **5**.

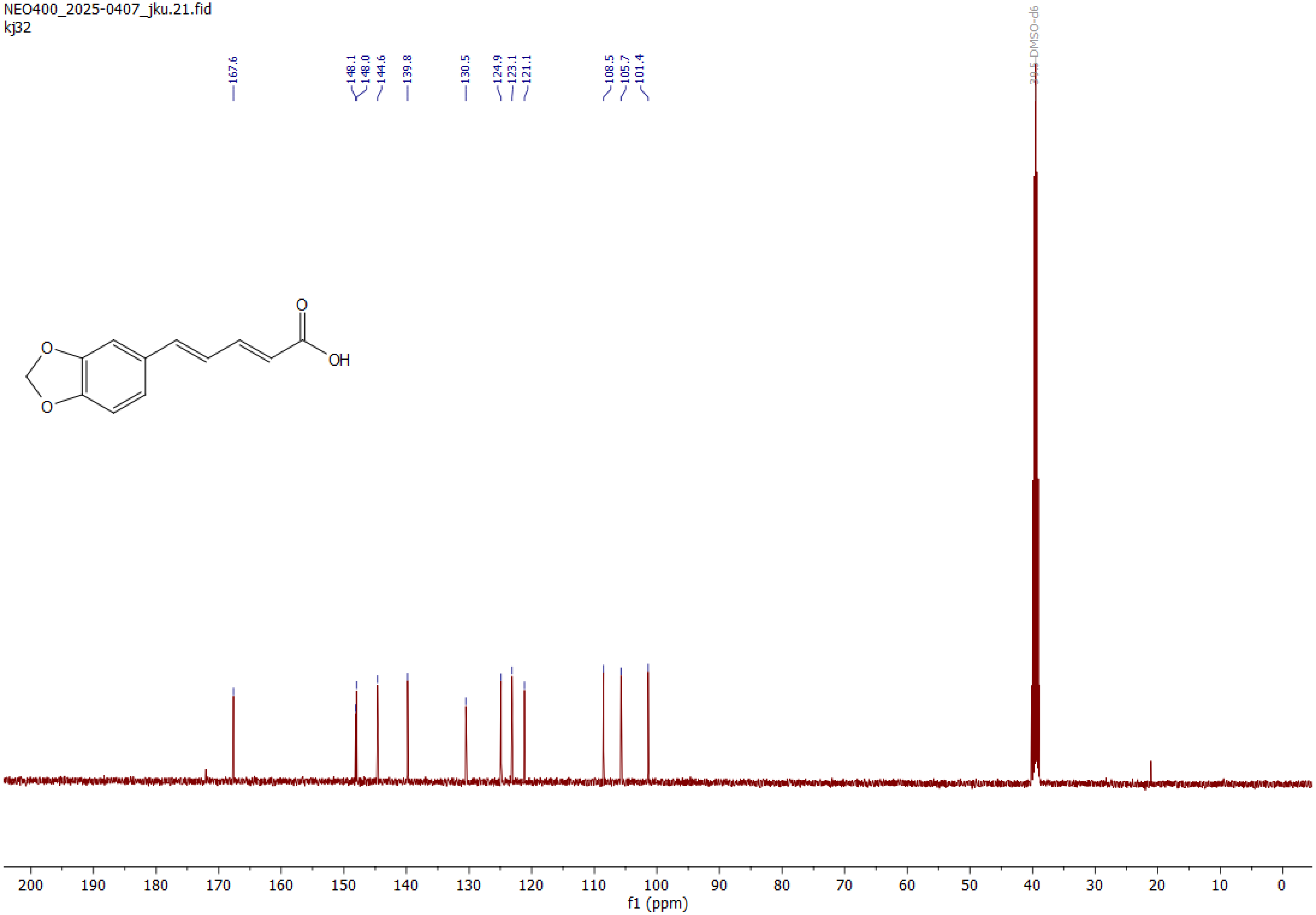

Figure S20: ^{1}H NMR (400 MHz, methanol-d_4) of **7a**.

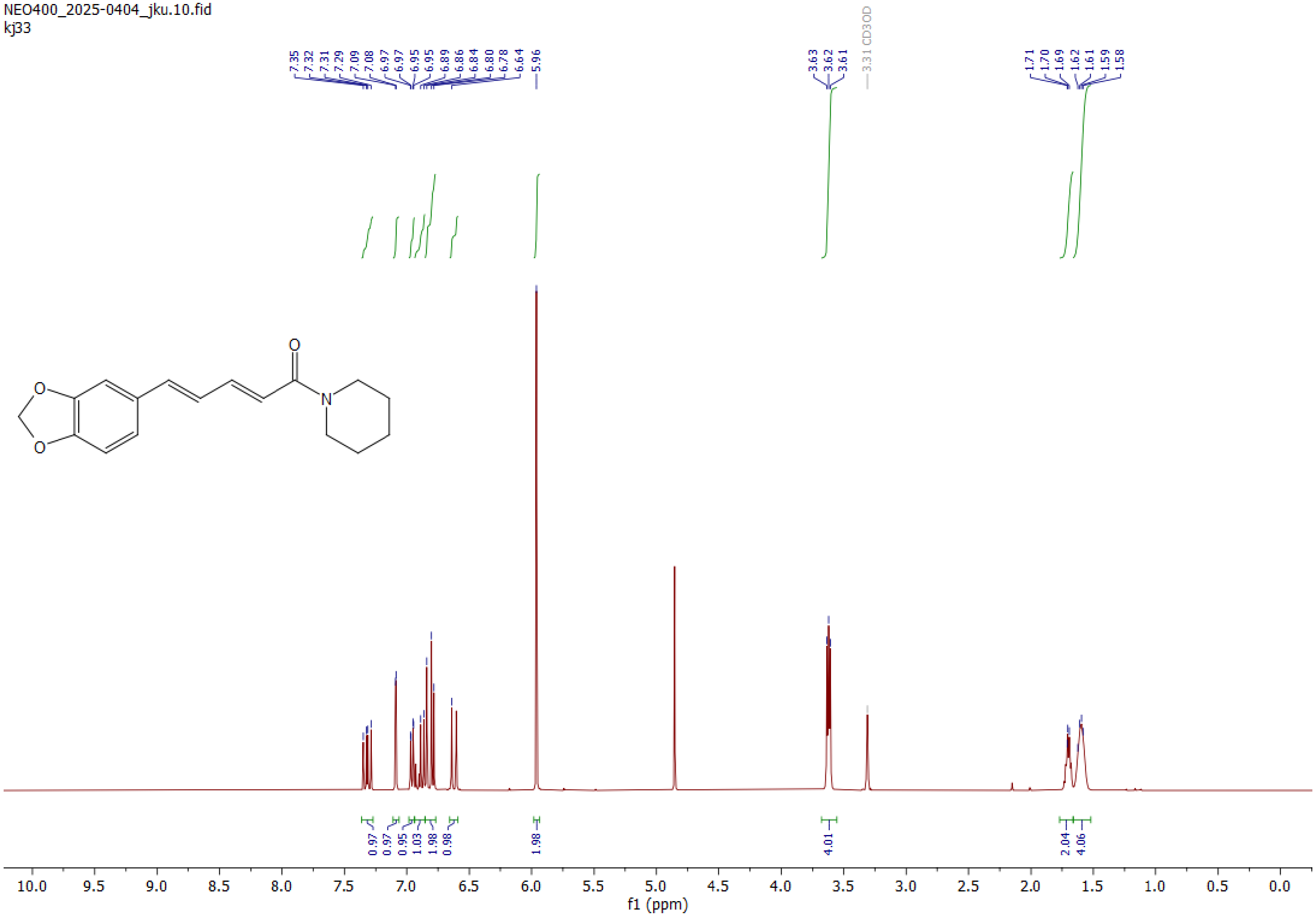

Figure S21: $^{13}C\{^{1}H\}$ NMR (100 MHz, methanol-d_4) of **7a**.

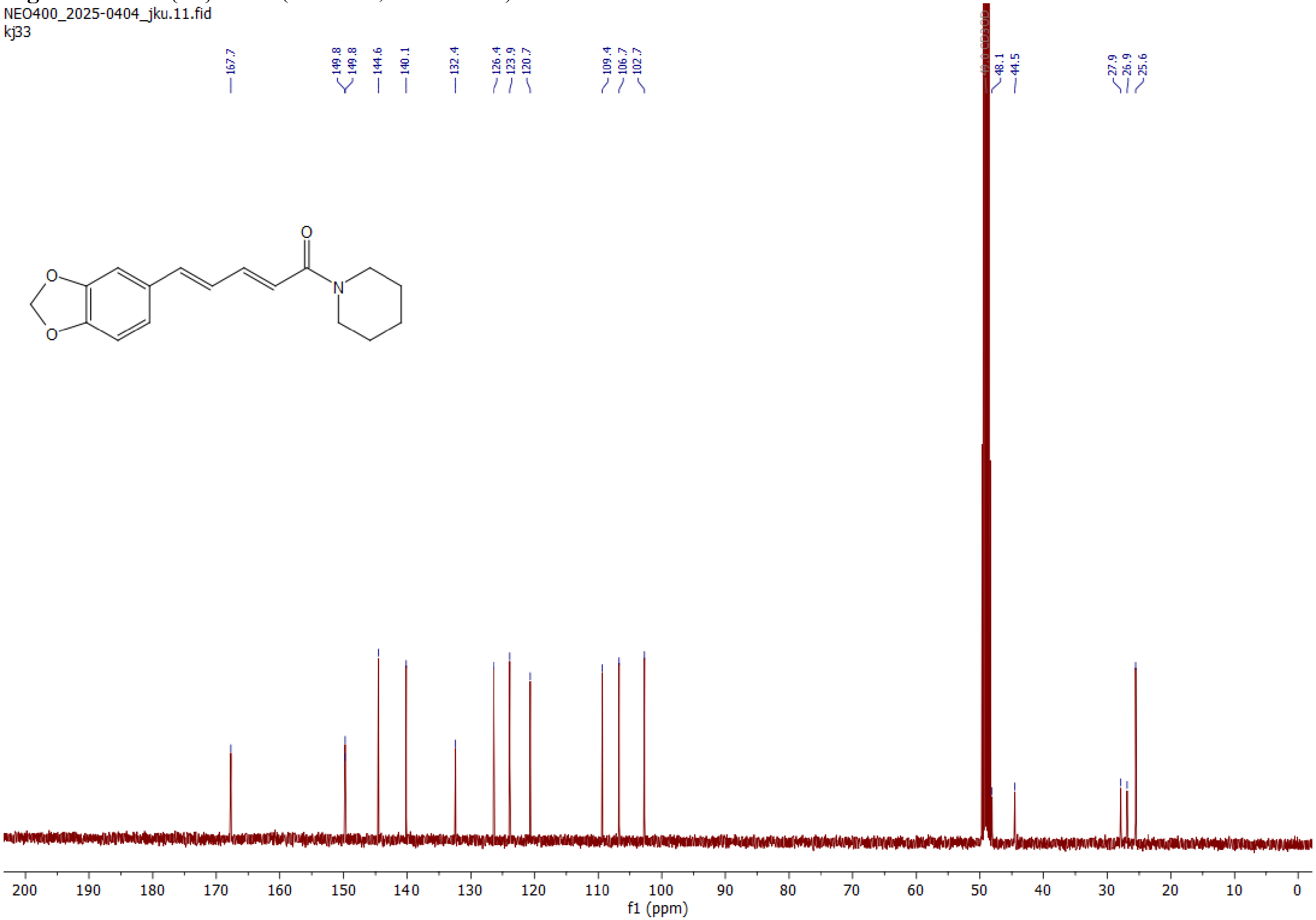

Figure S22: ^{1}H NMR (400 MHz, $CDCl_3$) of **7b**.

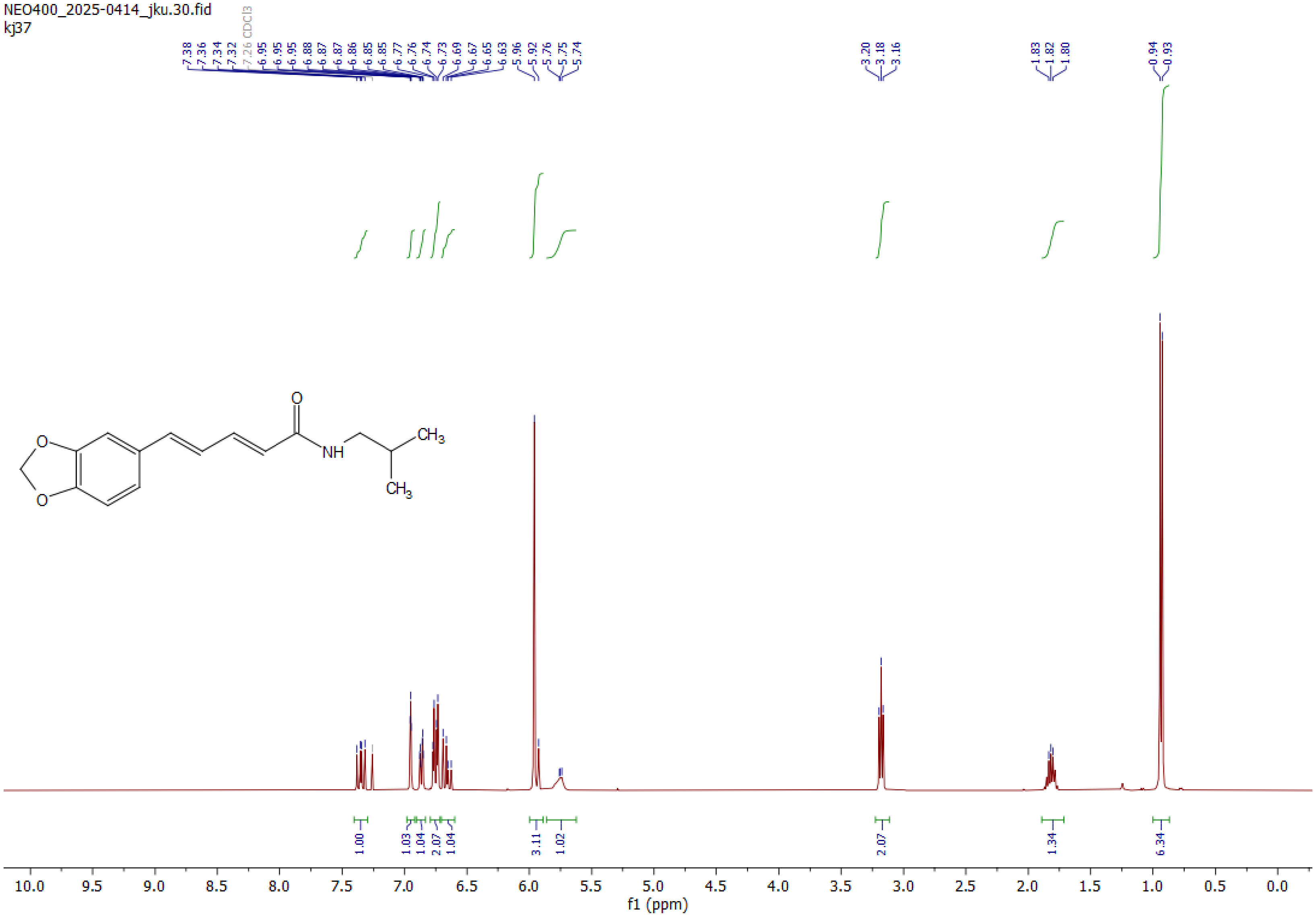

Figure S23: $^{13}C\{^1H\}$ NMR (100 MHz, $CDCl_3$) of **7b**.

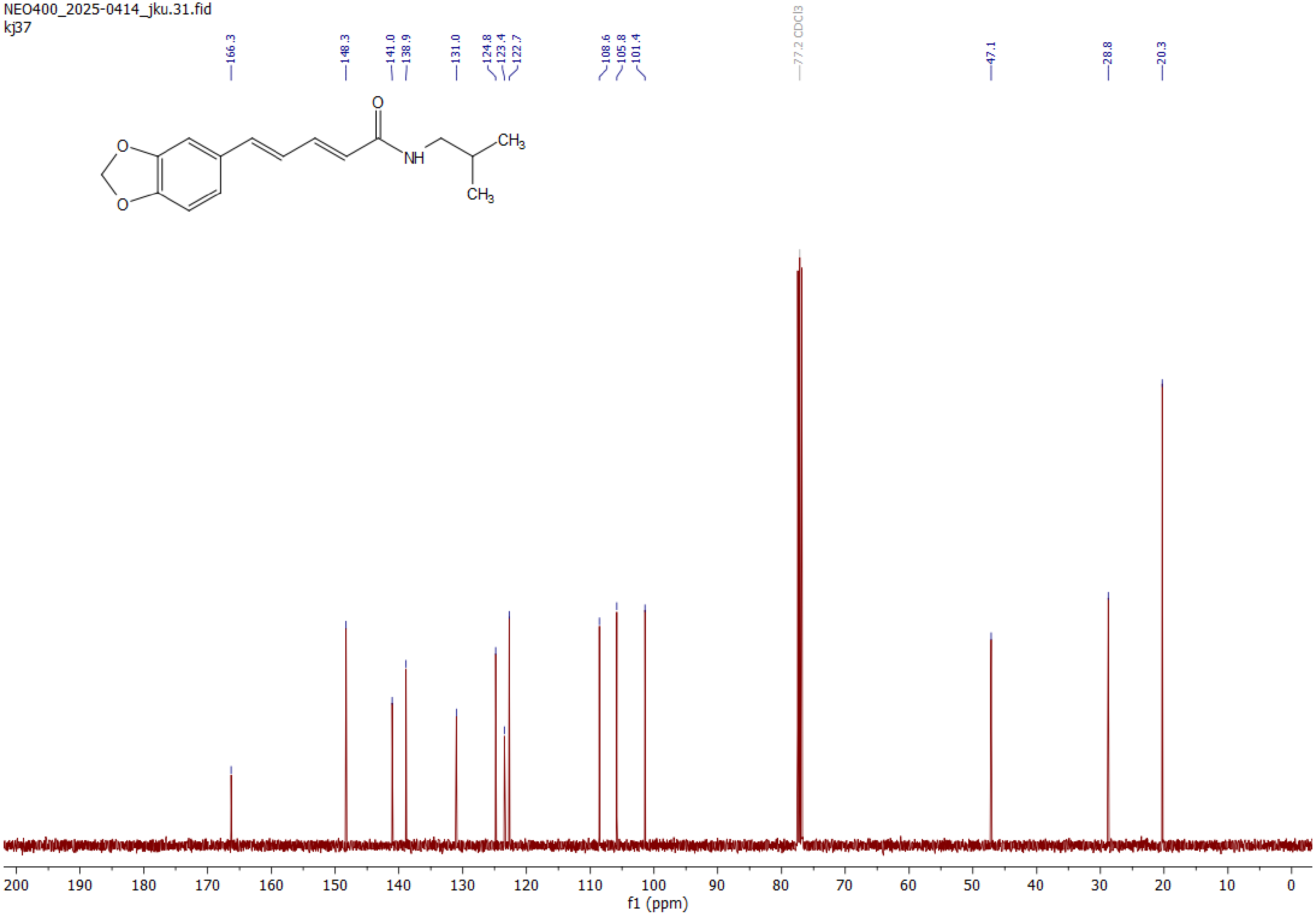

Figure S24: ^{1}H NMR (400 MHz, $CDCl_3$) of **7c**.

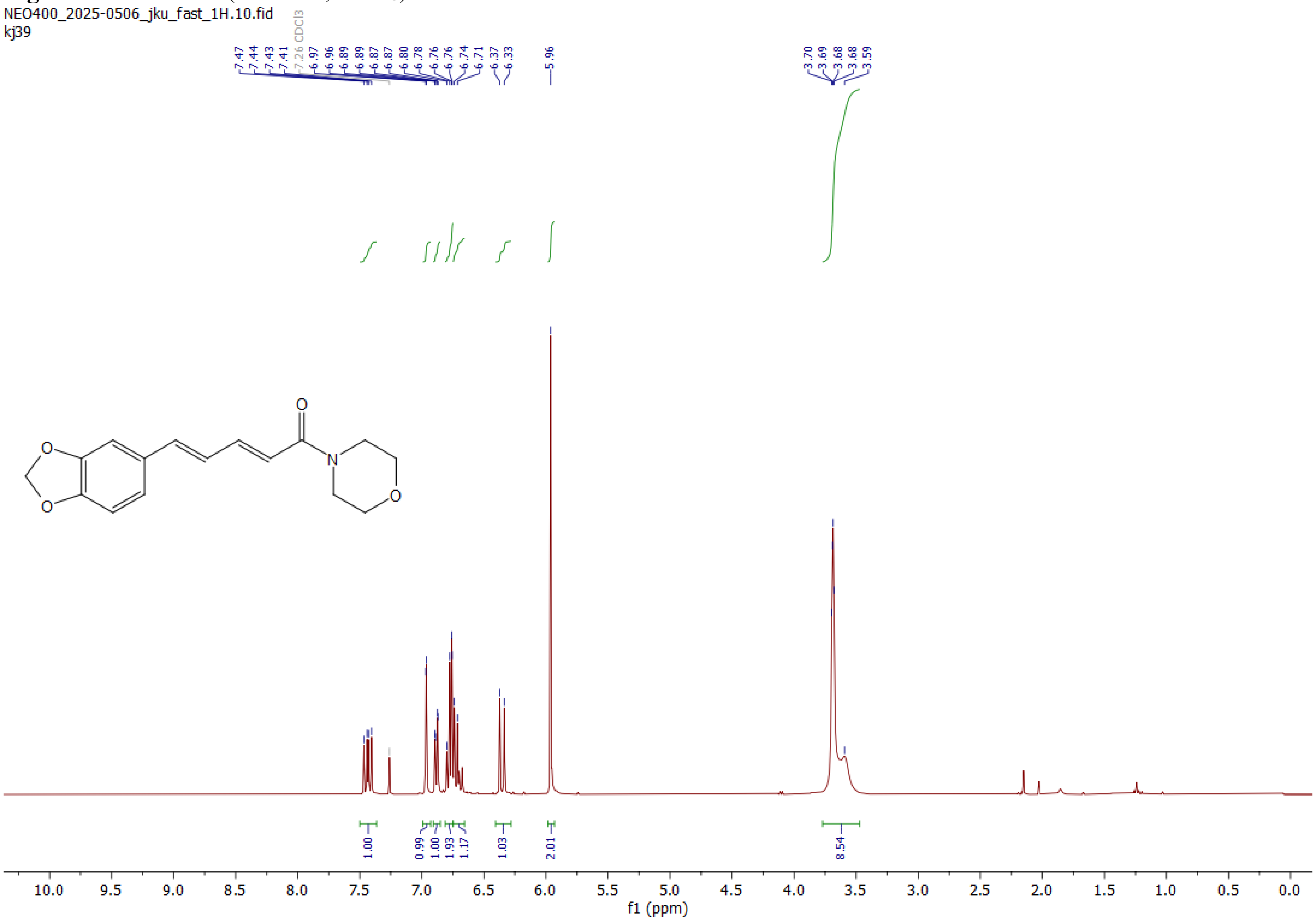

Figure S25: $^{13}C\{^1H\}$ NMR (100 MHz, $CDCl_3$) of **7c**.

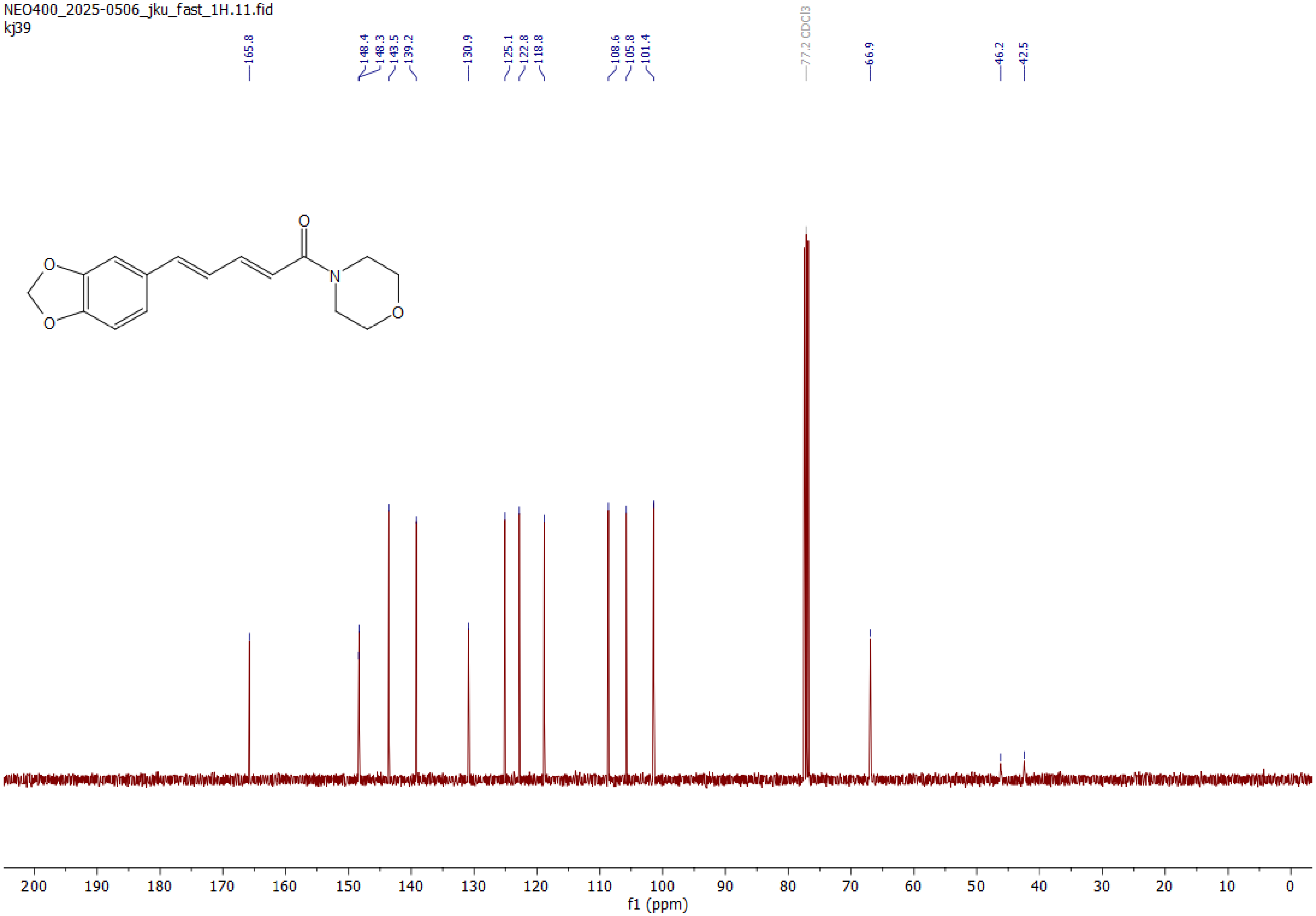

Figure S26: H,H-COSY (400 MHz, $CDCl_3$) of **7c**.

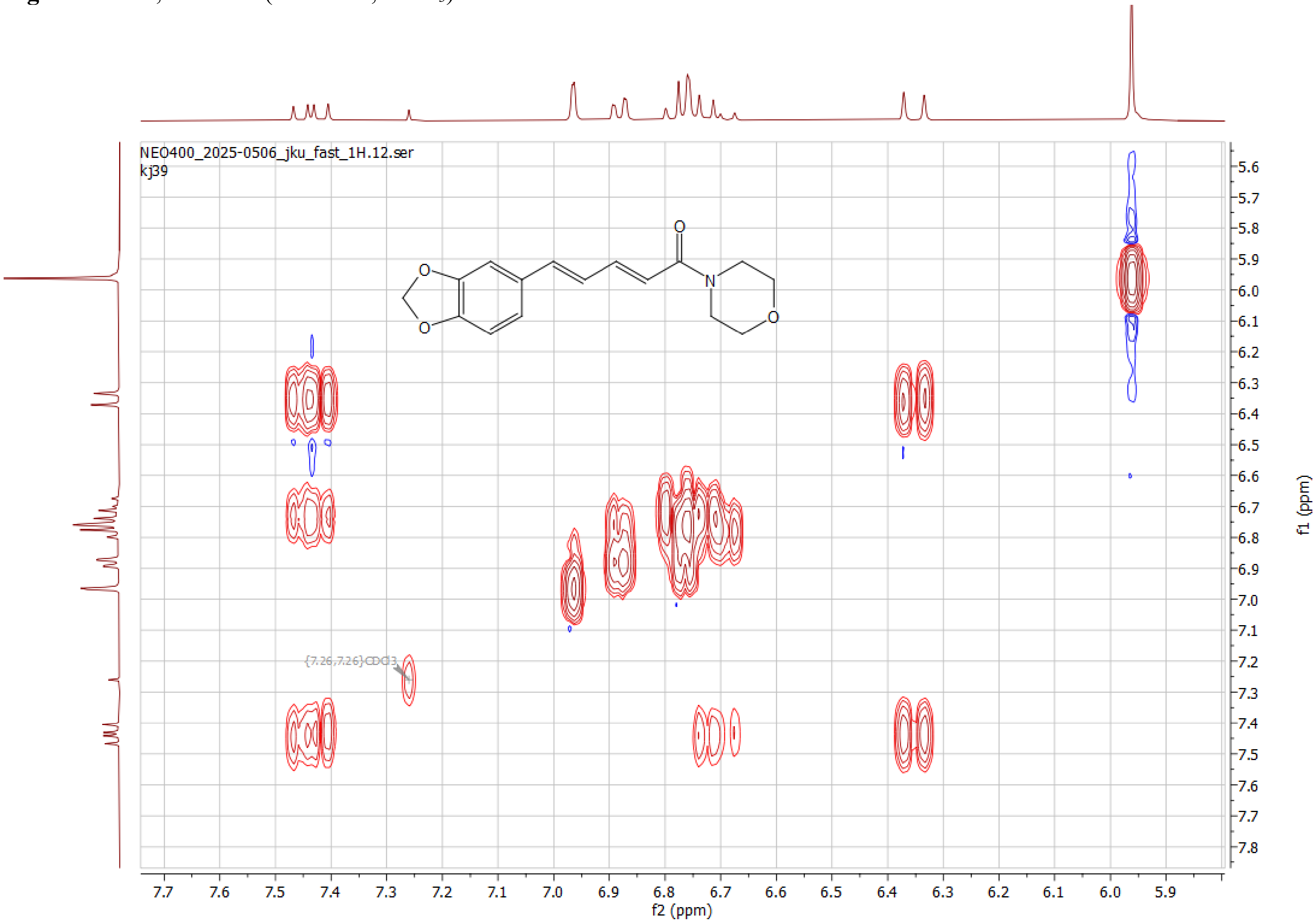

Figure S27: HSQC (400/100 MHz, $CDCl_3$) of **7c**.

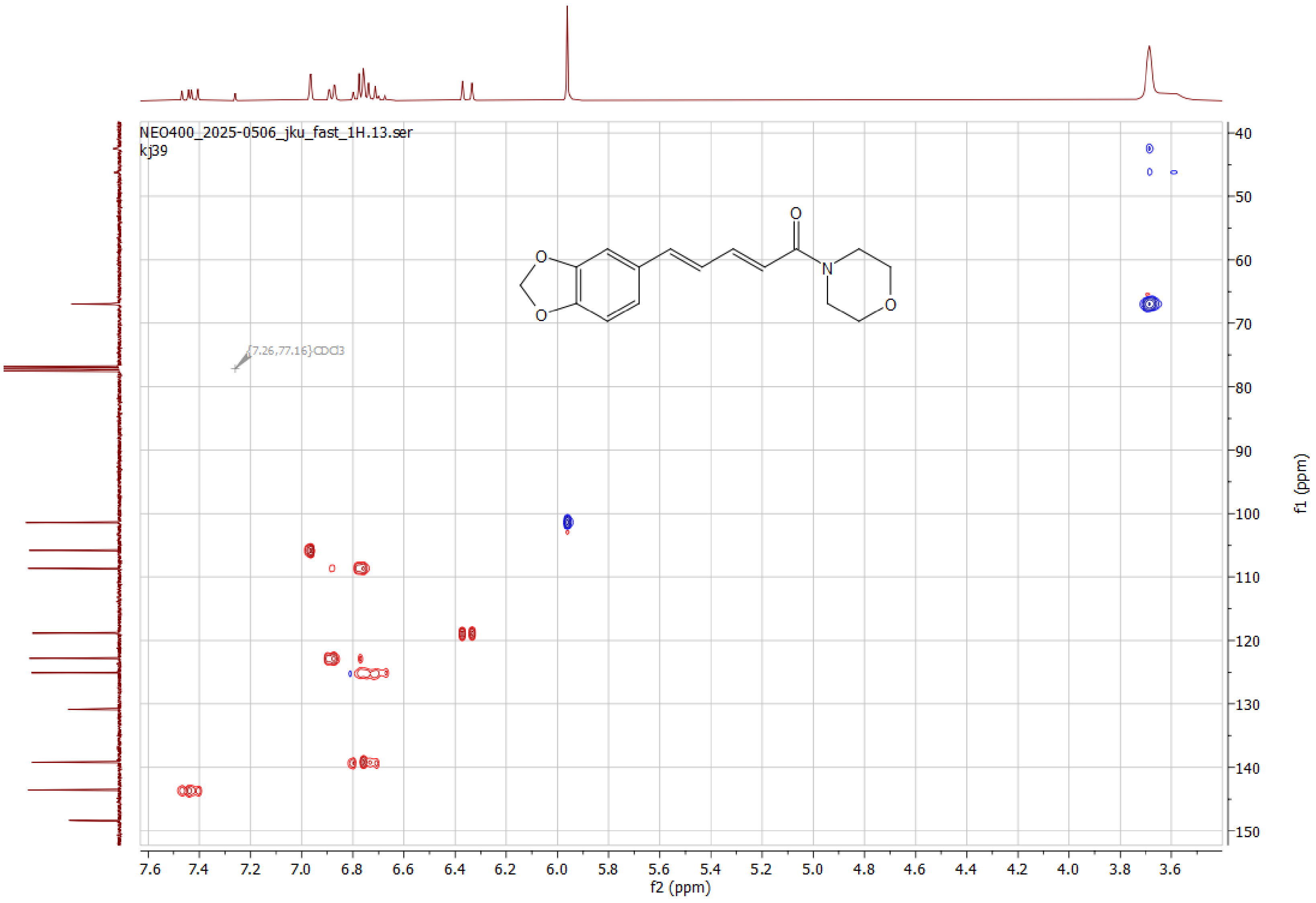

Figure S28: HMBC (400/100 MHz, $CDCl_3$) of **7c**.

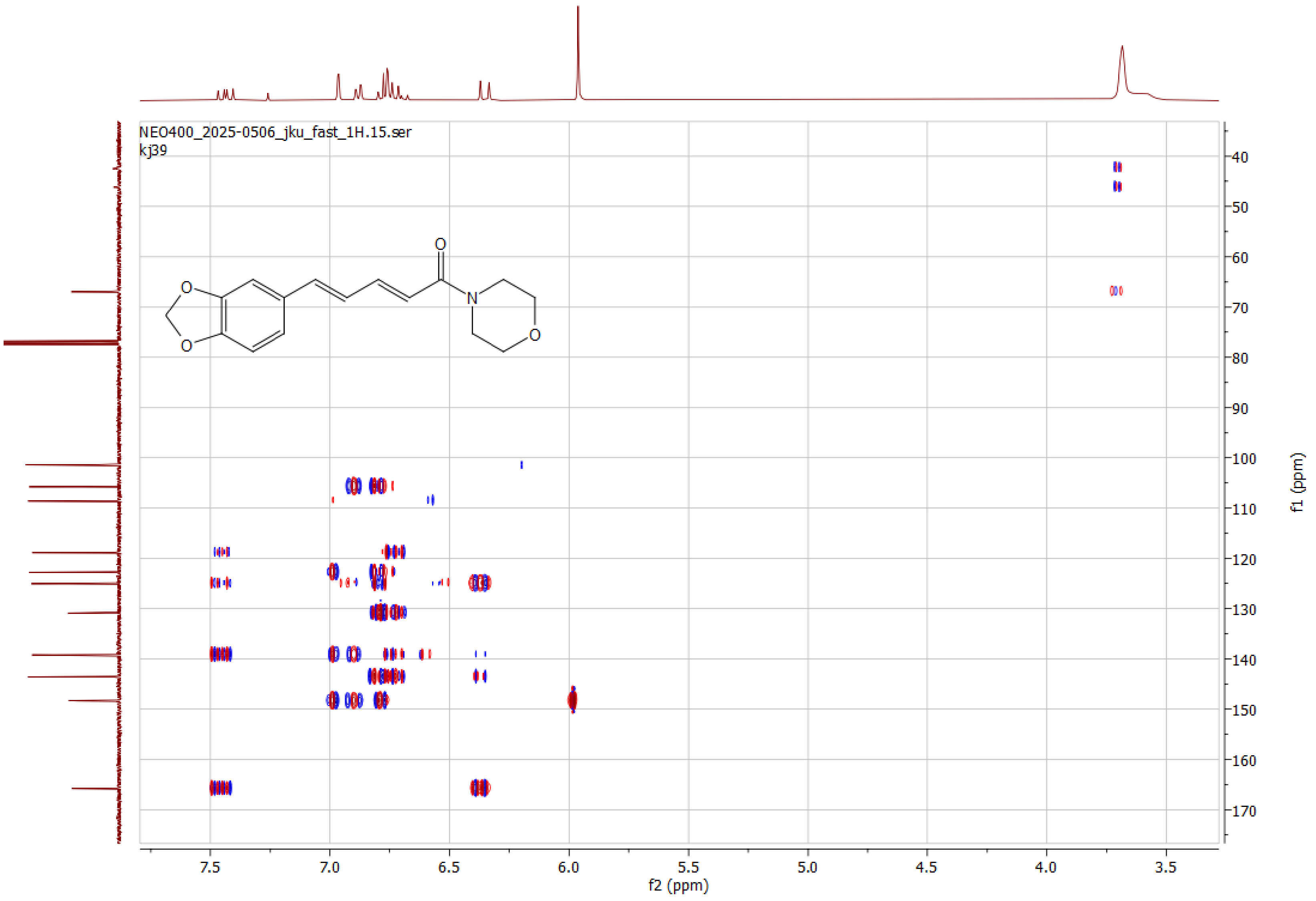

Figure S29: ^{1}H NMR (400 MHz, $CDCl_3$) of **7d**.

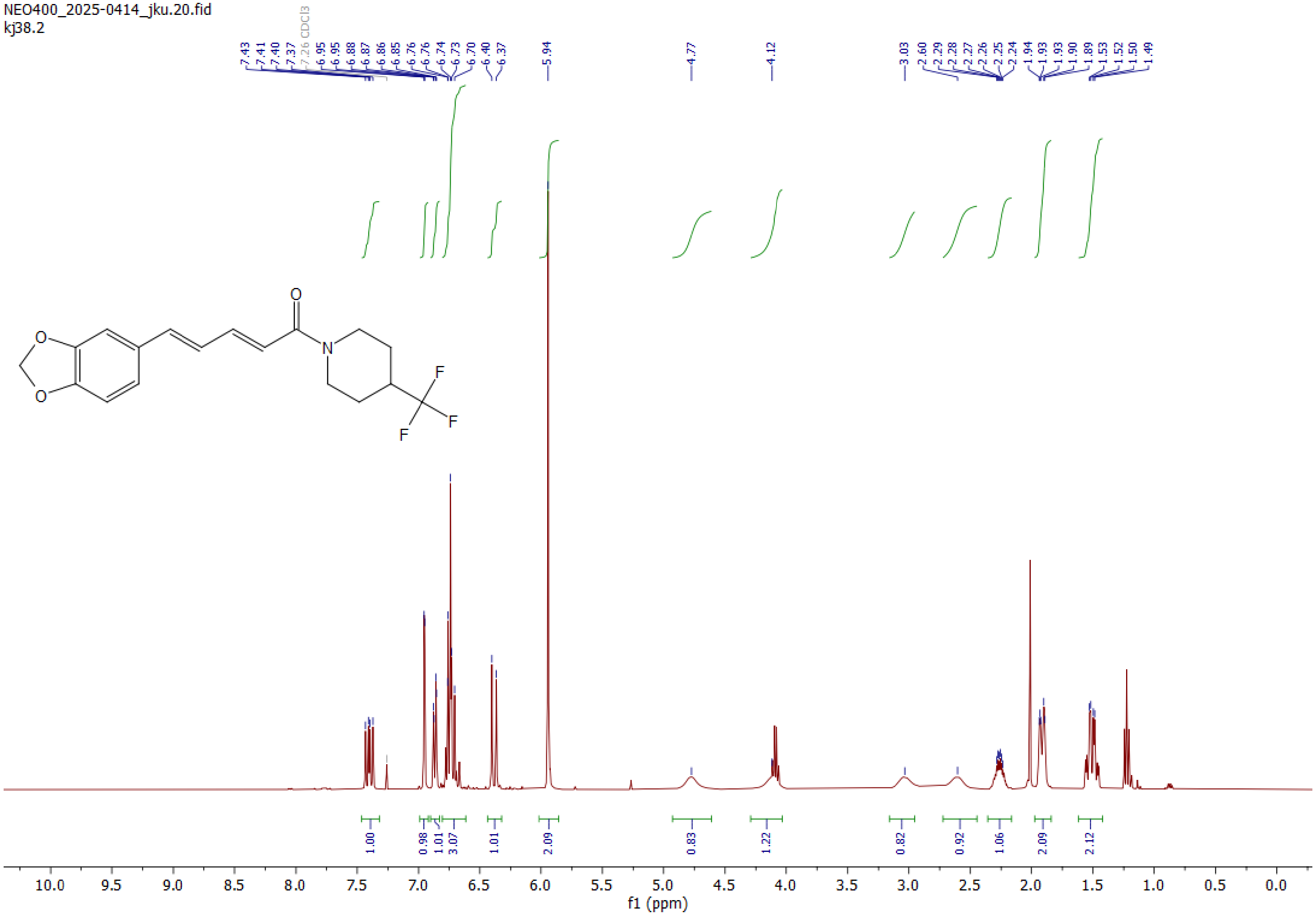

Figure S30: $^{13}C\{^{1}H\}$ NMR (100 MHz, $CDCl_3$) of **7d**.

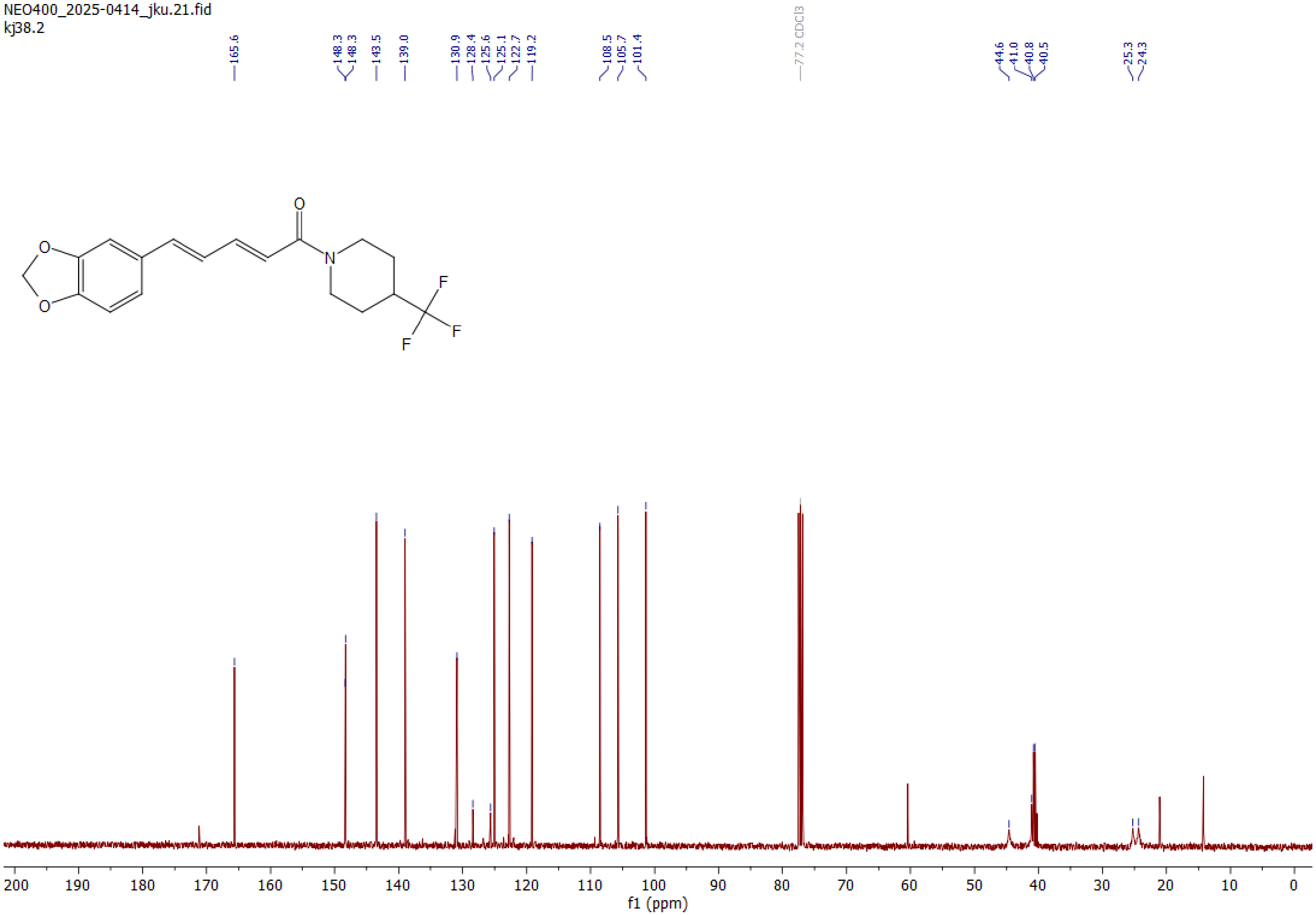

Figure S31: ^{1}H NMR (400 MHz, $CDCl_3$) of **7e**.

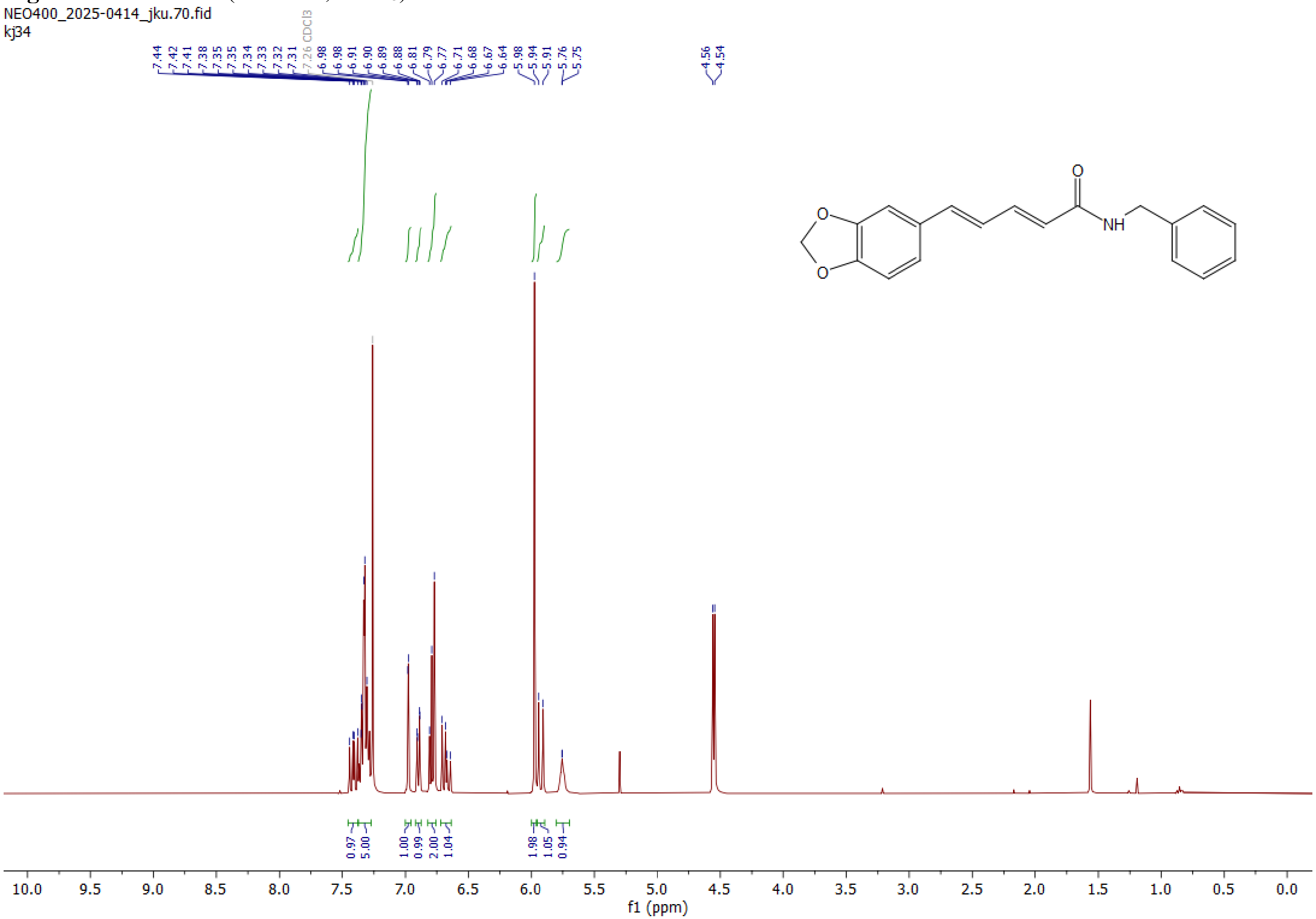

Figure S32: $^{13}C\{^1H\}$ NMR (100 MHz, $CDCl_3$) of **7e**.

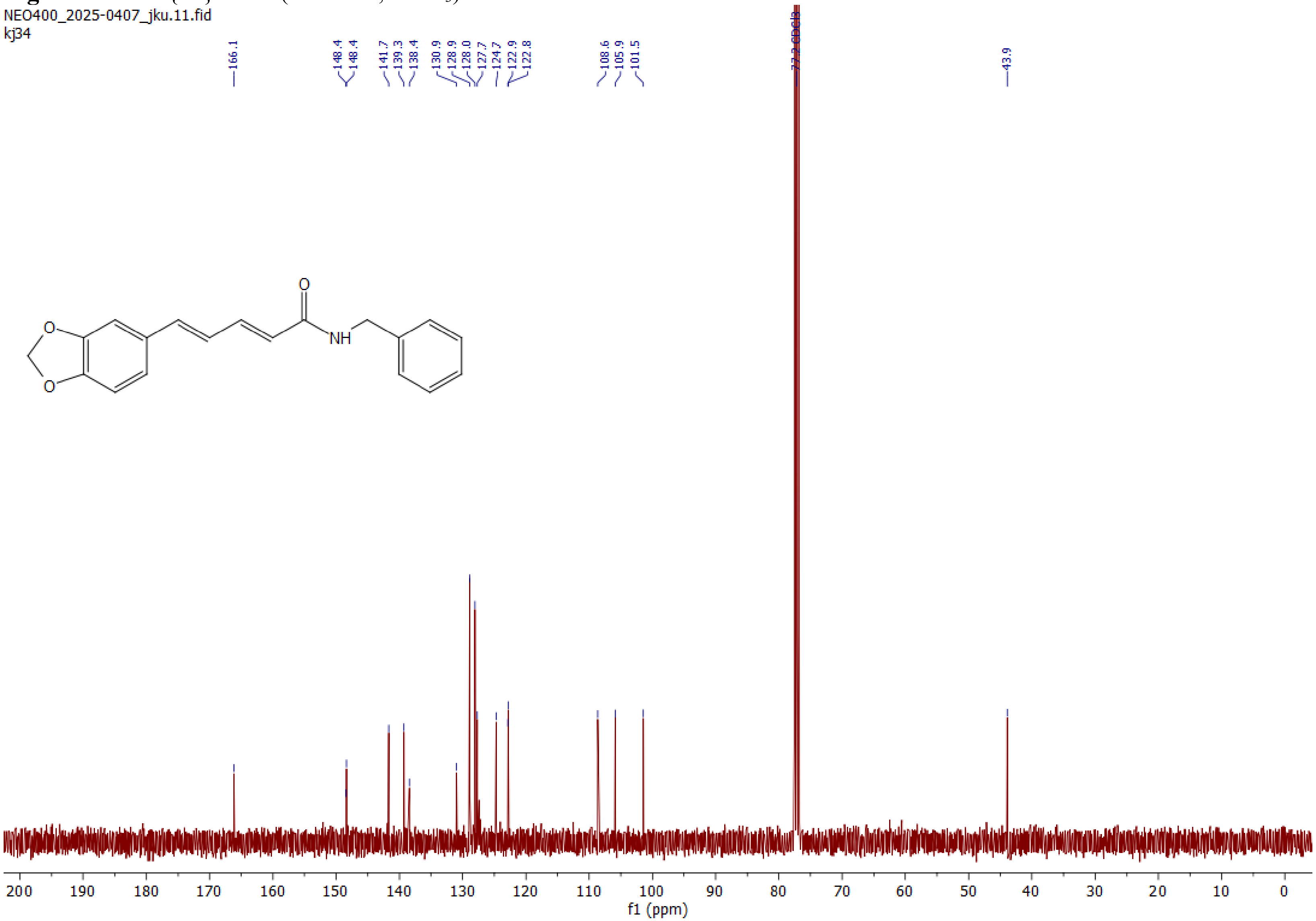

Figure S33: ^{1}H NMR (400 MHz, DMSO-d_6) of **7f**.

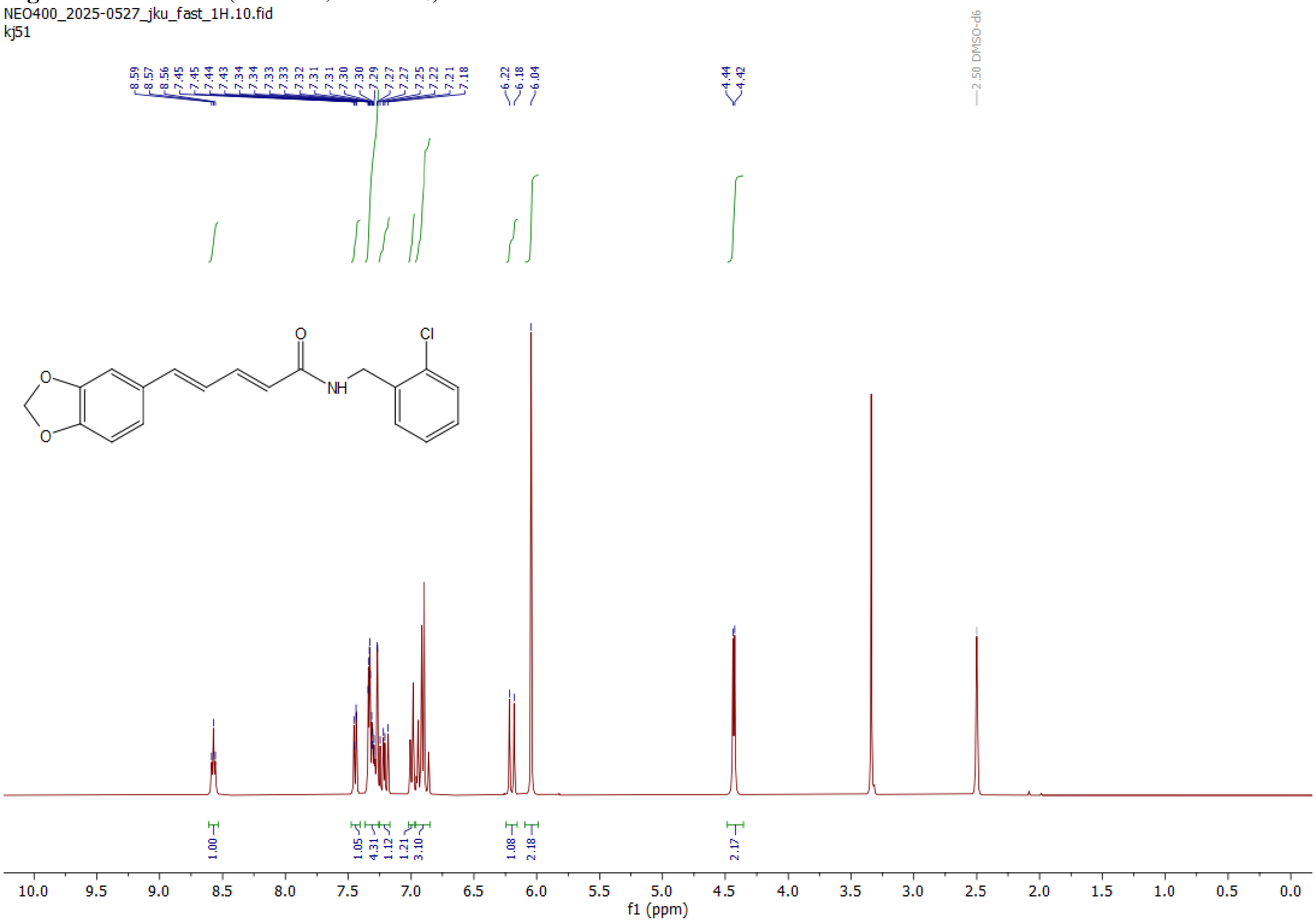

Figure S34: $^{13}C\{^{1}H\}$ NMR (100 MHz, DMSO-d_6) of **7f**.

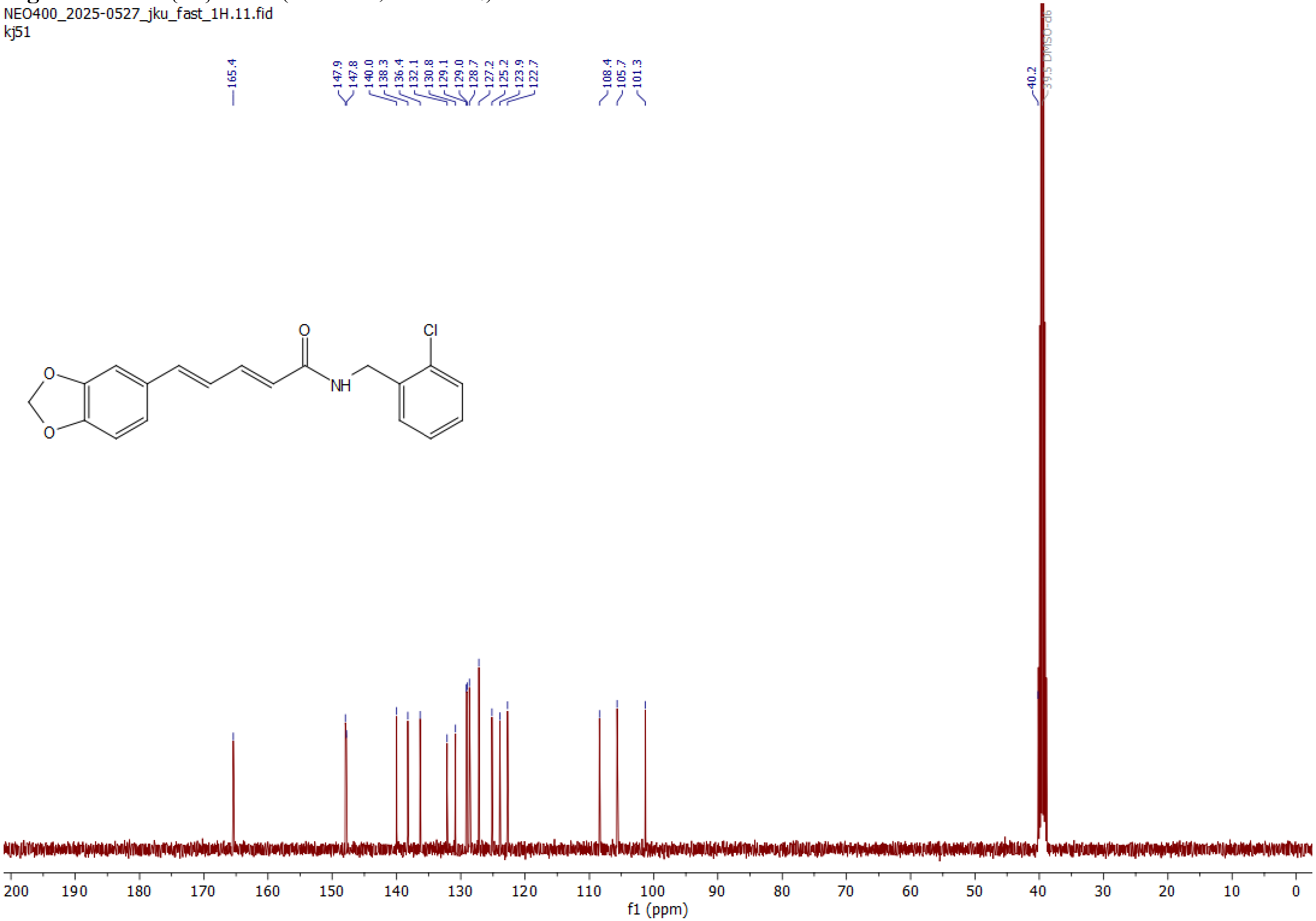

Figure S35: H,H-COSY (400 MHz, DMSO-d_6) of **7f**.

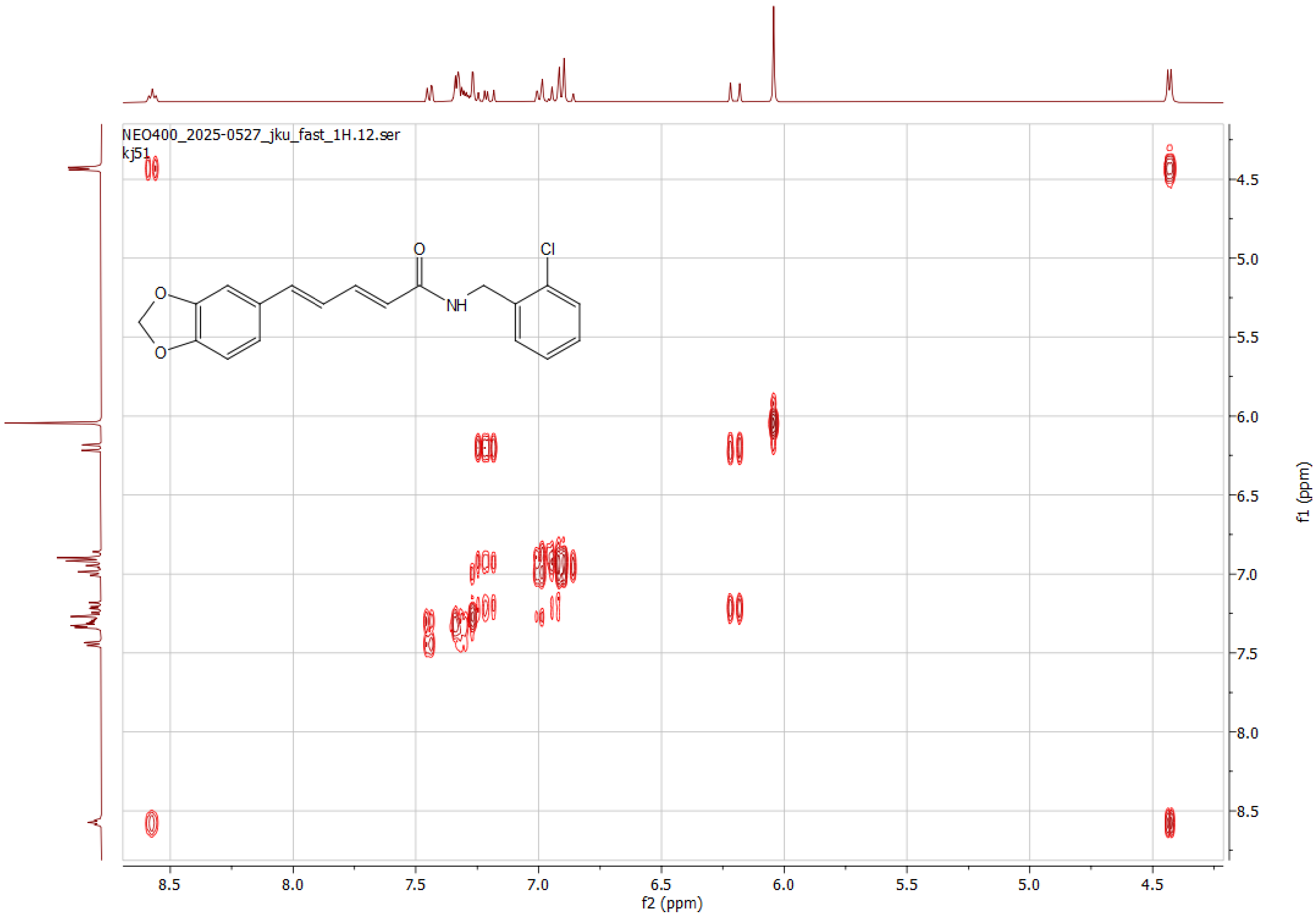

Figure S36: HSQC (400/100 MHz, DMSO-d_6) of **7f**.

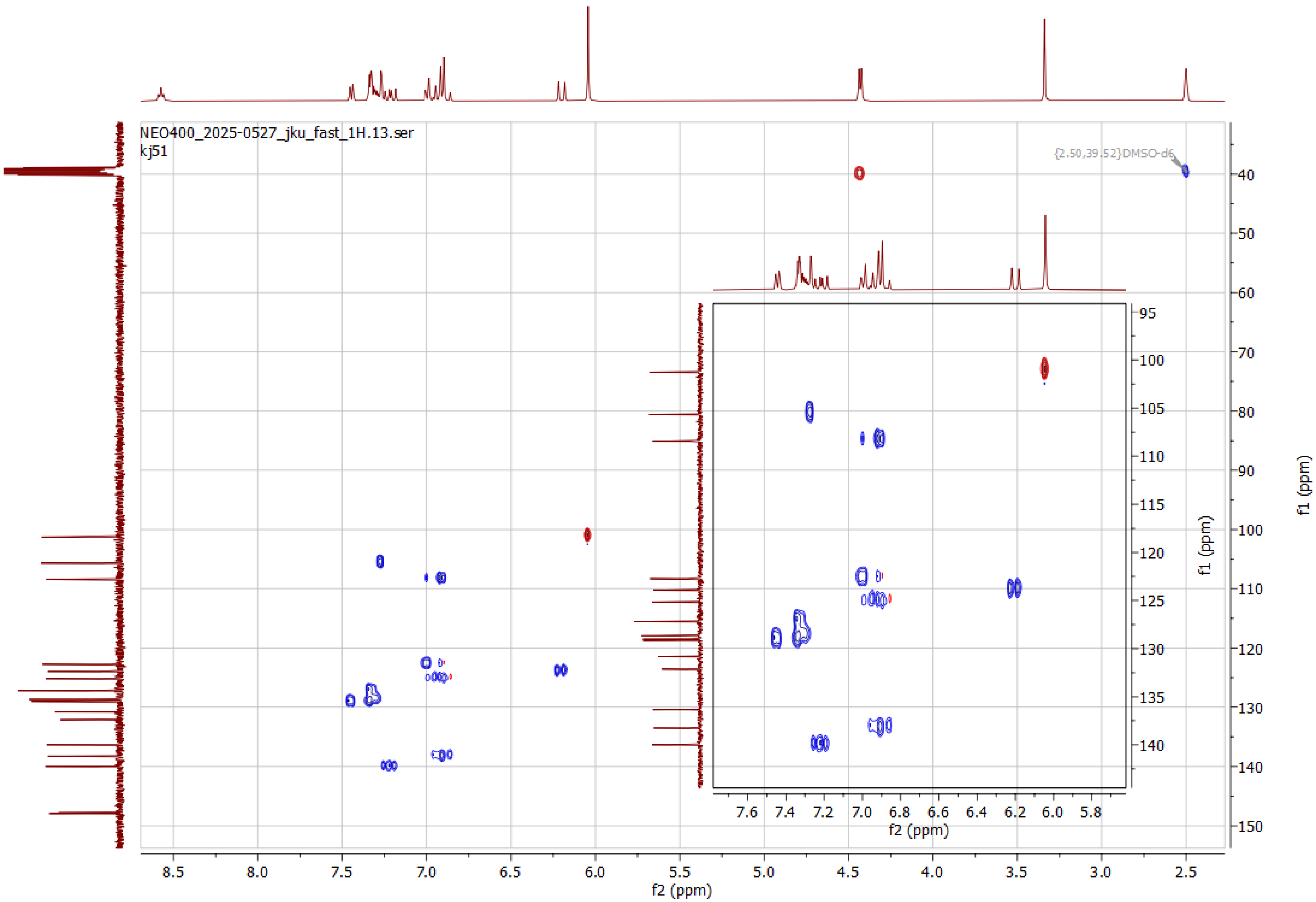

Figure S37: HMBC (400/100 MHz, DMSO-d_6) of **7f**.

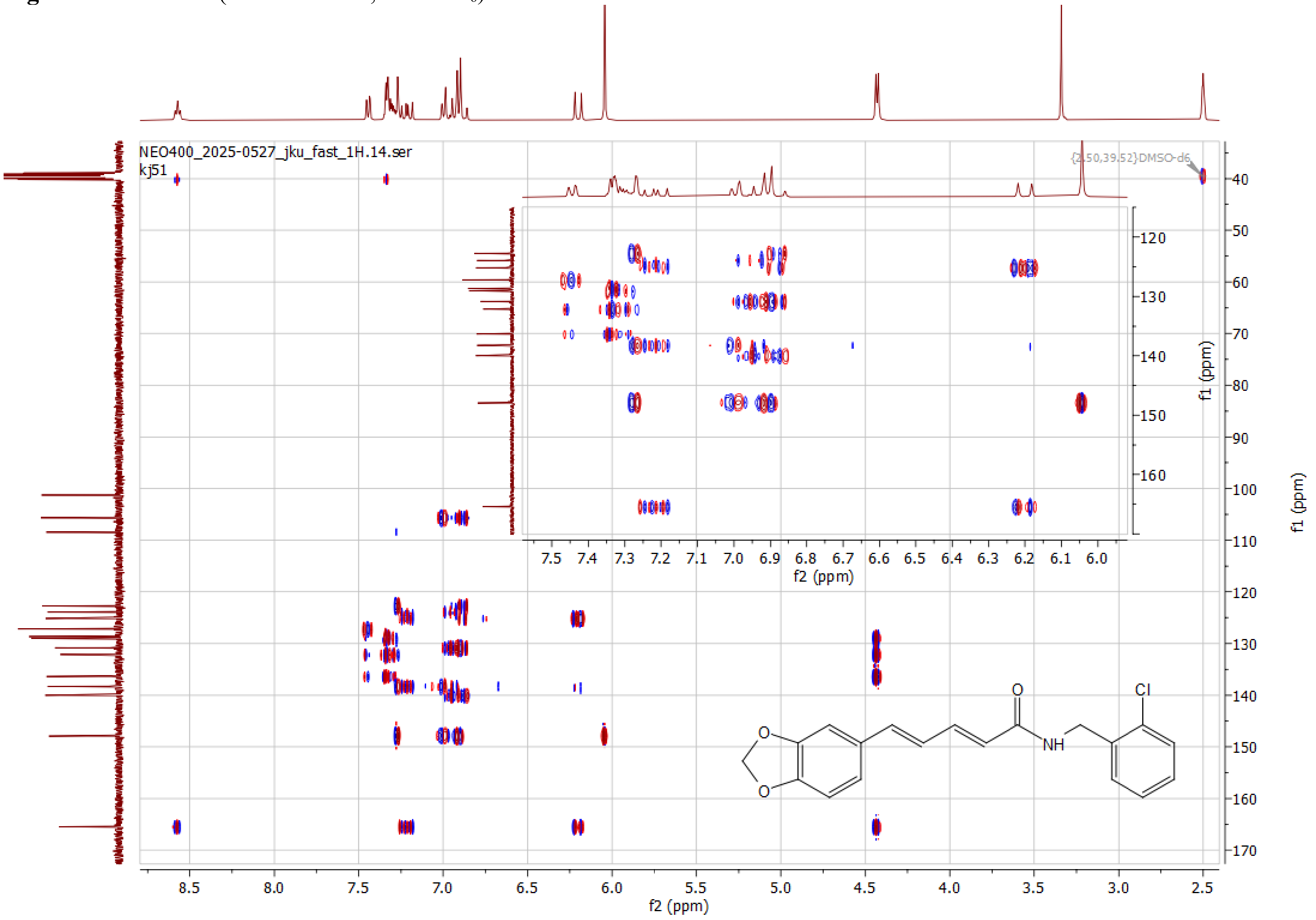

Figure S38: ^{1}H NMR (400 MHz, DMSO-d_6) of **7g**.

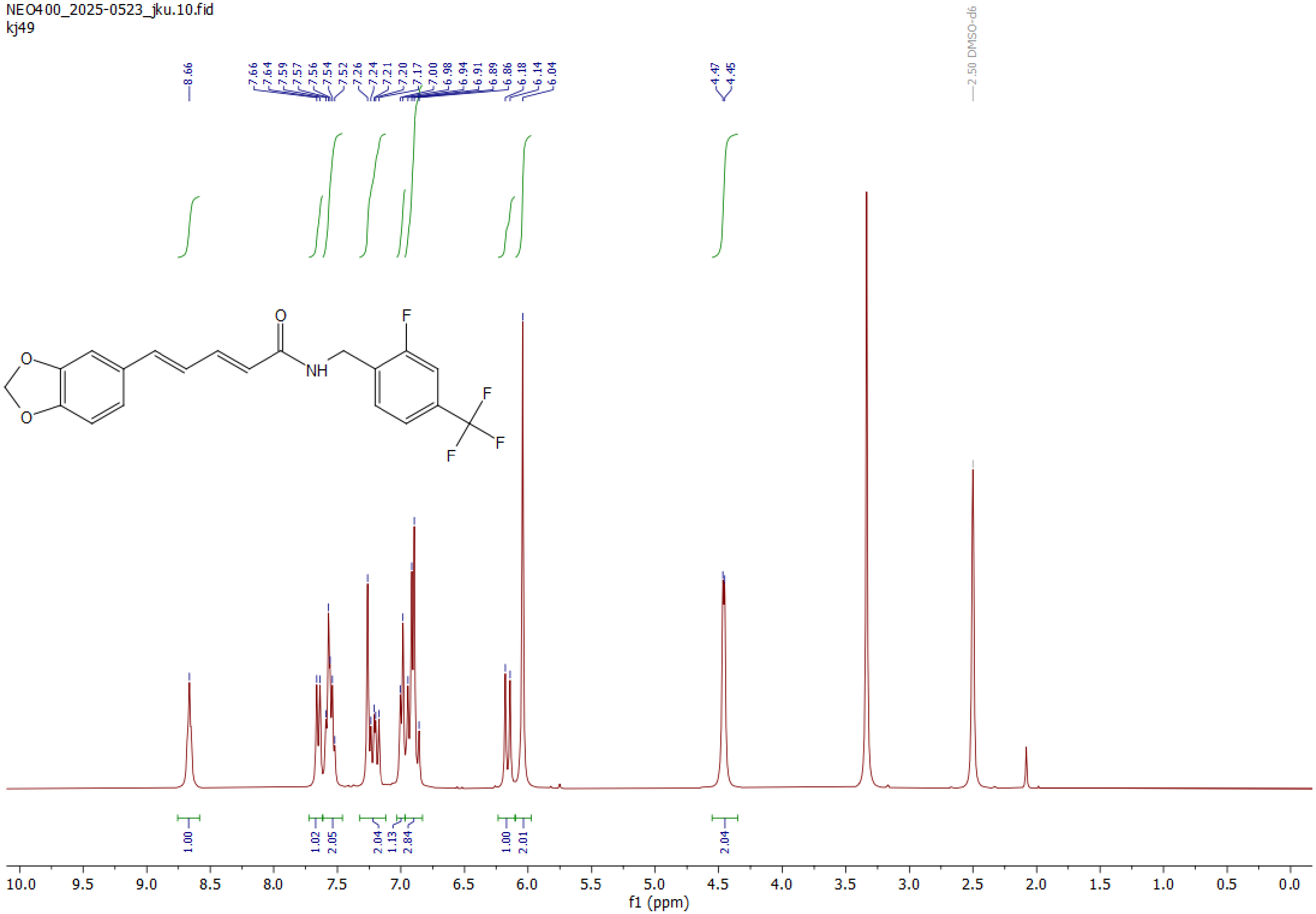

Figure S39: $^{13}C\{^1H\}$ NMR (100 MHz, DMSO-d_6) of **7g**.

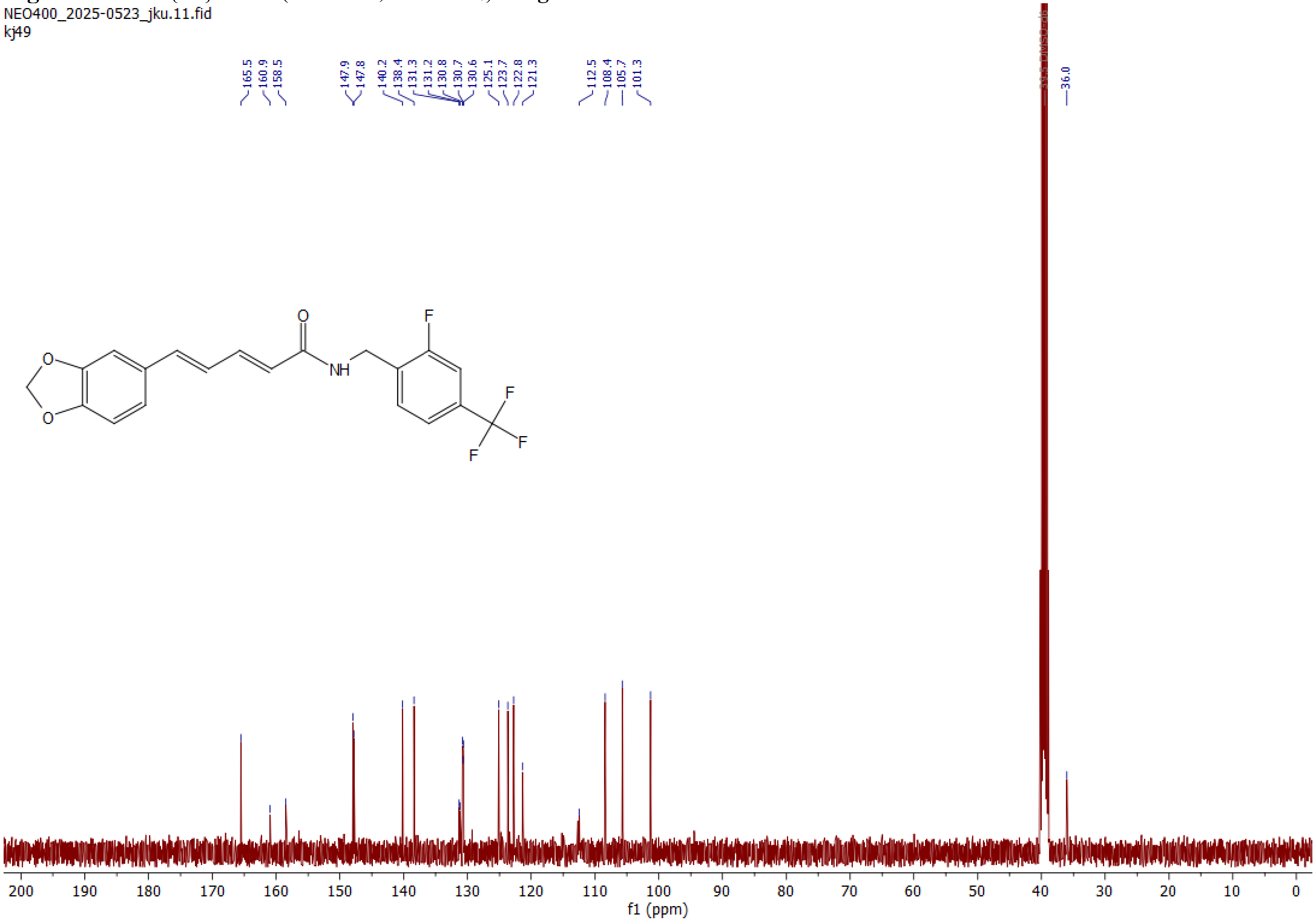

Figure S40: H,H-COSY (400 MHz, DMSO-d_6) of **7g**.

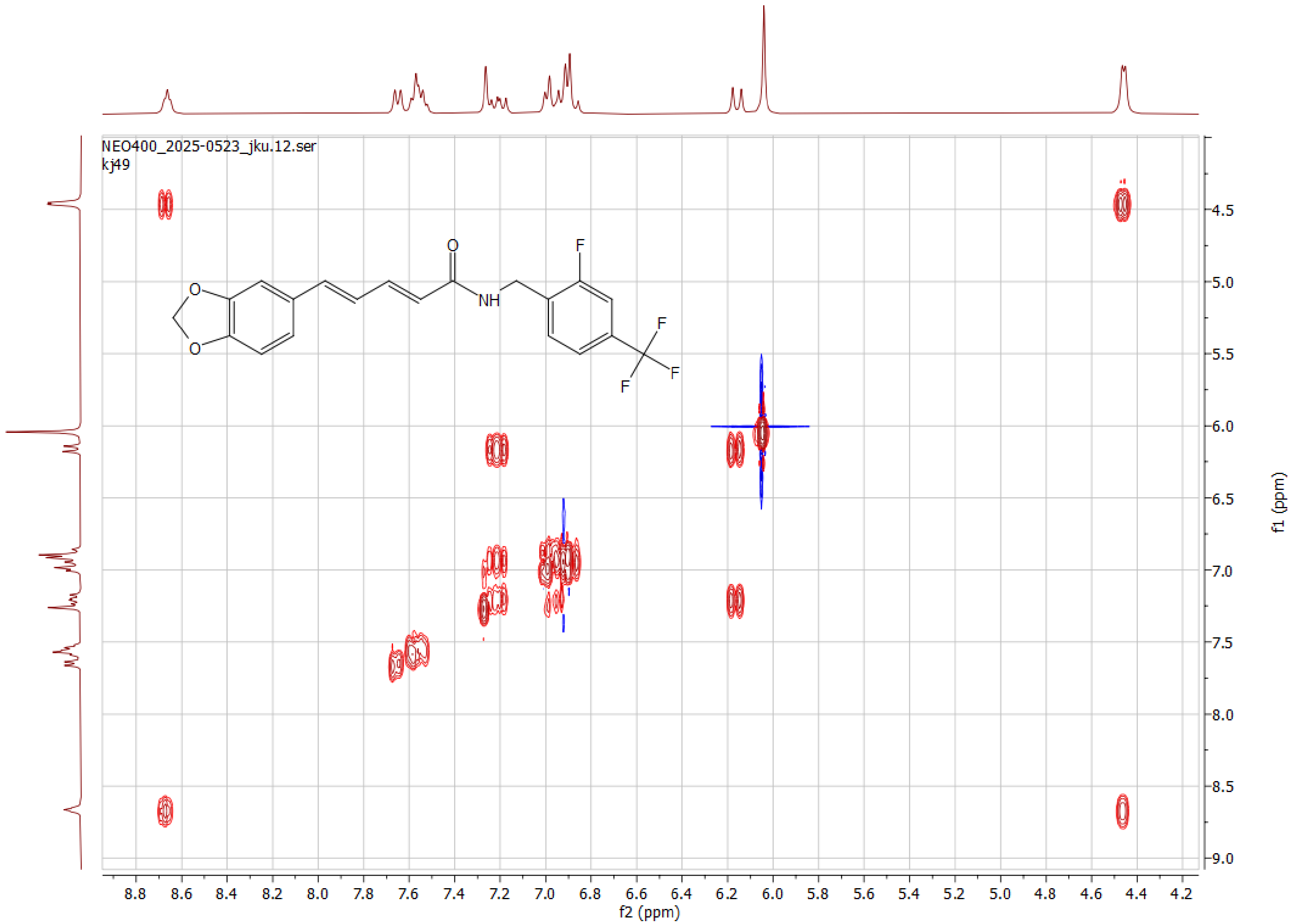

Figure S41: HSQC (400/100 MHz, DMSO-d_6) of **7g**.

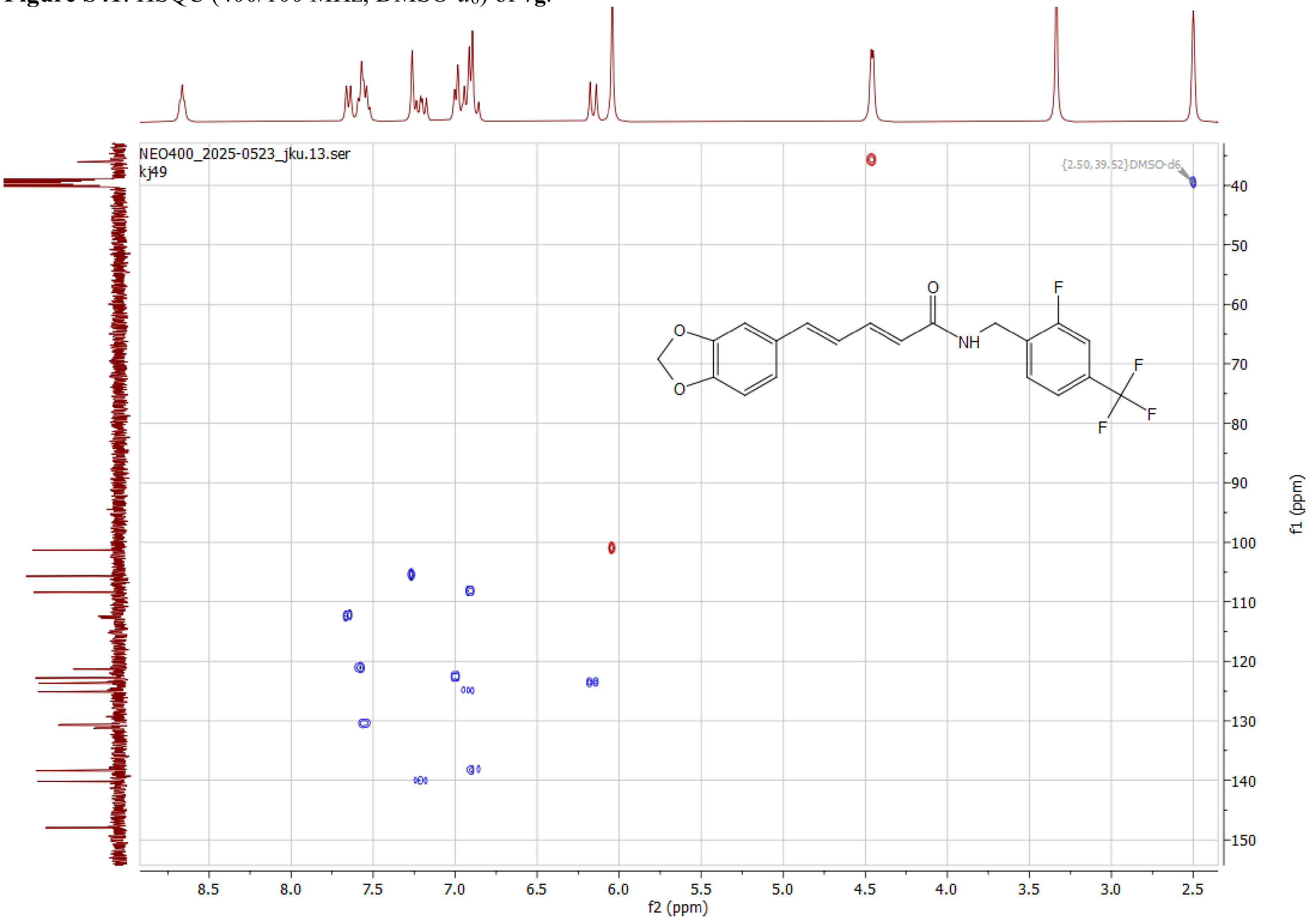

Figure S42: HMBC (400/100 MHz, DMSO-d_6) of **7g**.

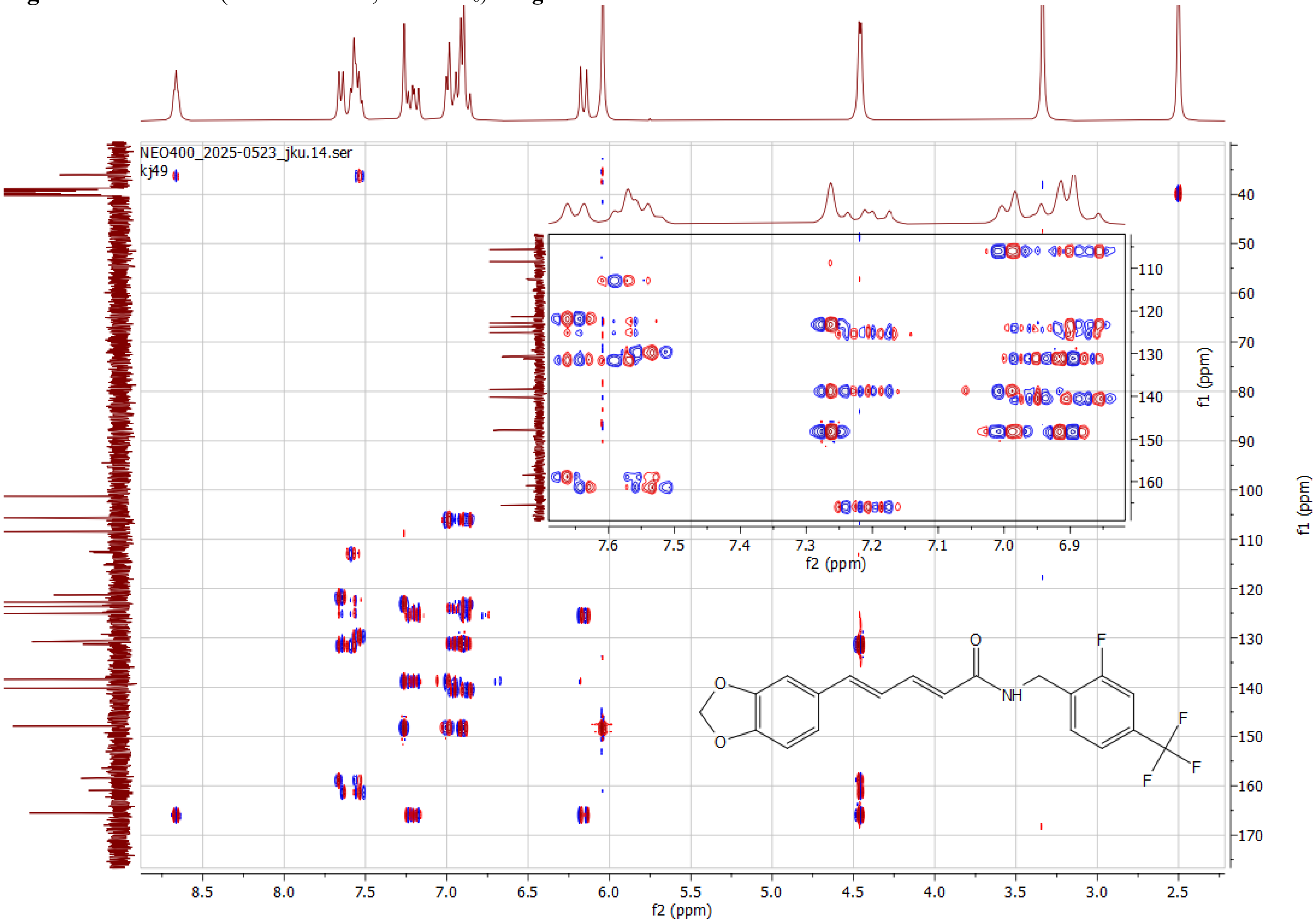

Figure S43: ^{1}H NMR (400 MHz, $CDCl_3$) of **7h**.

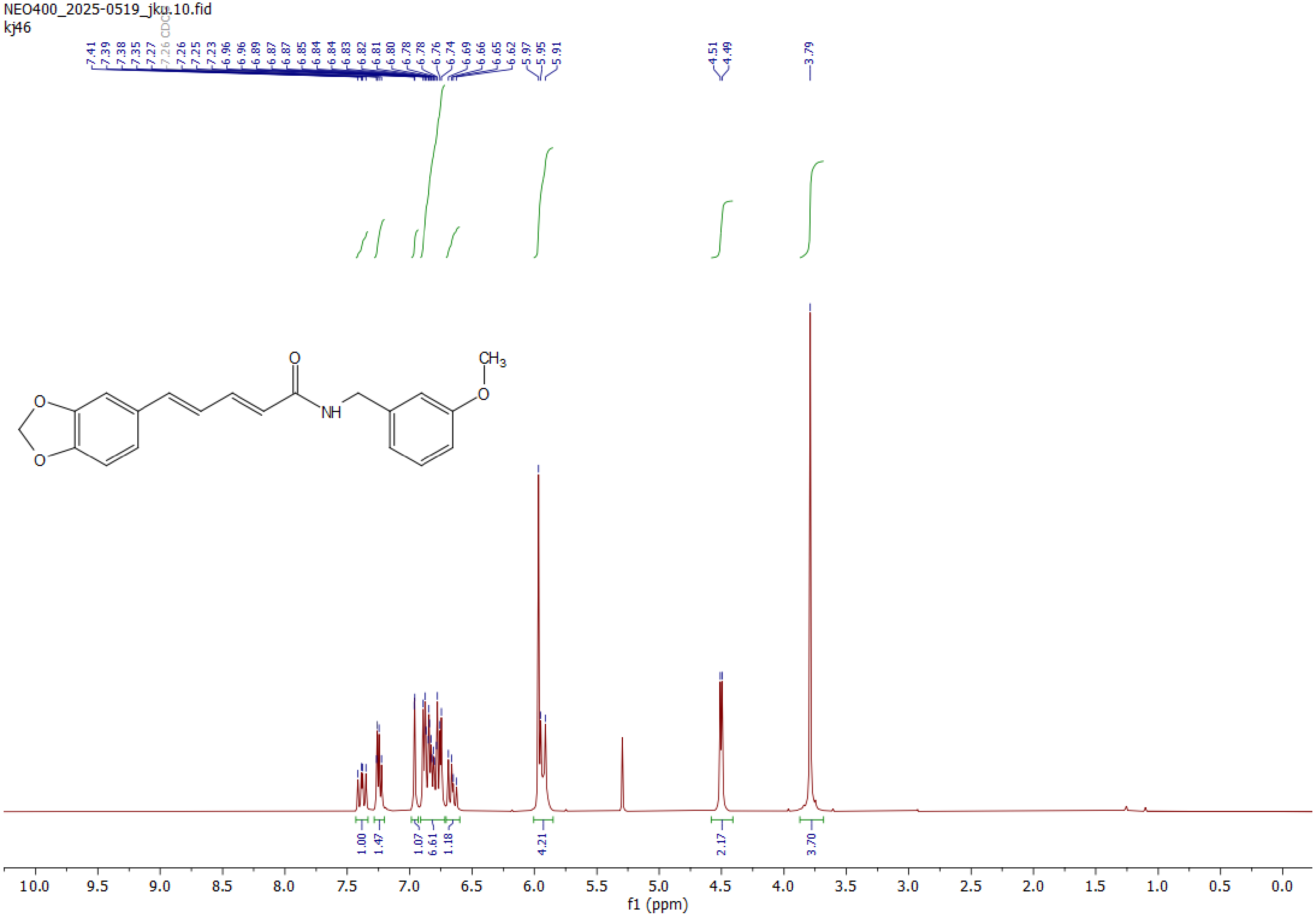

Figure S44: $^{13}C\{^{1}H\}$ NMR (100 MHz, $CDCl_3$) of **7h**.

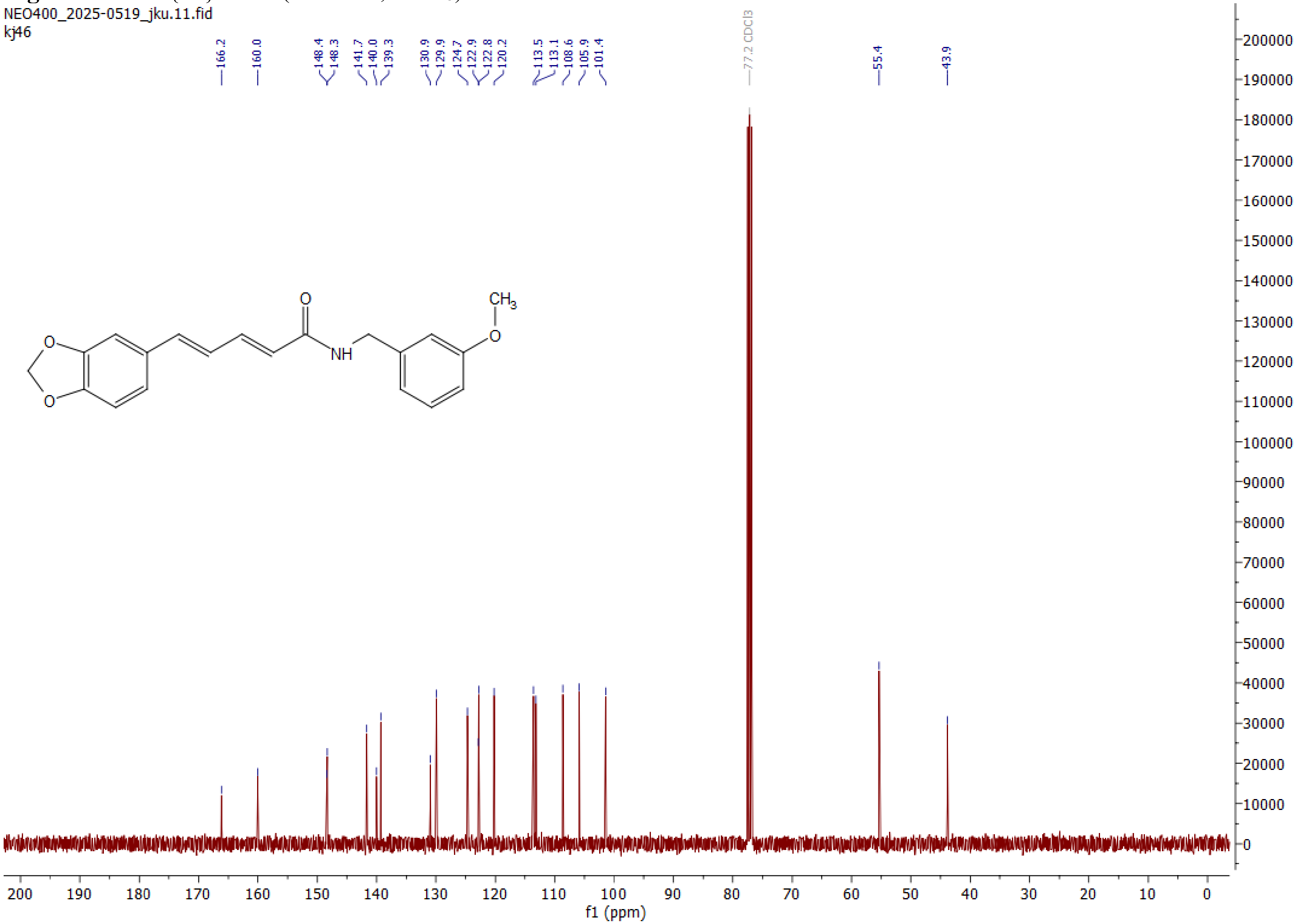

Figure S45: HSQC (400/100 MHz, $CDCl_3$) of **7h**.

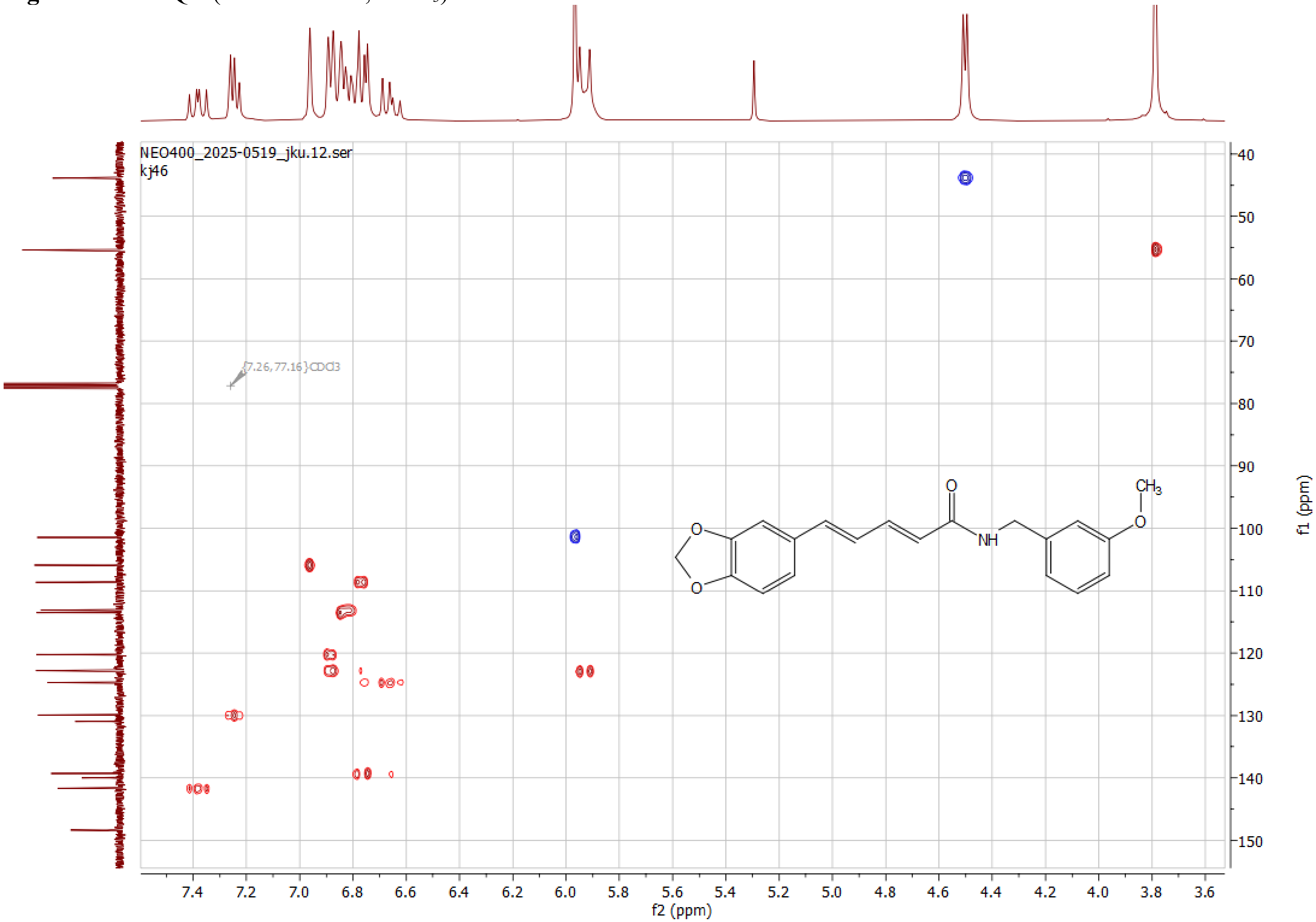

Figure S46: HMBC (400/100 MHz, $CDCl_3$) of **7h**.

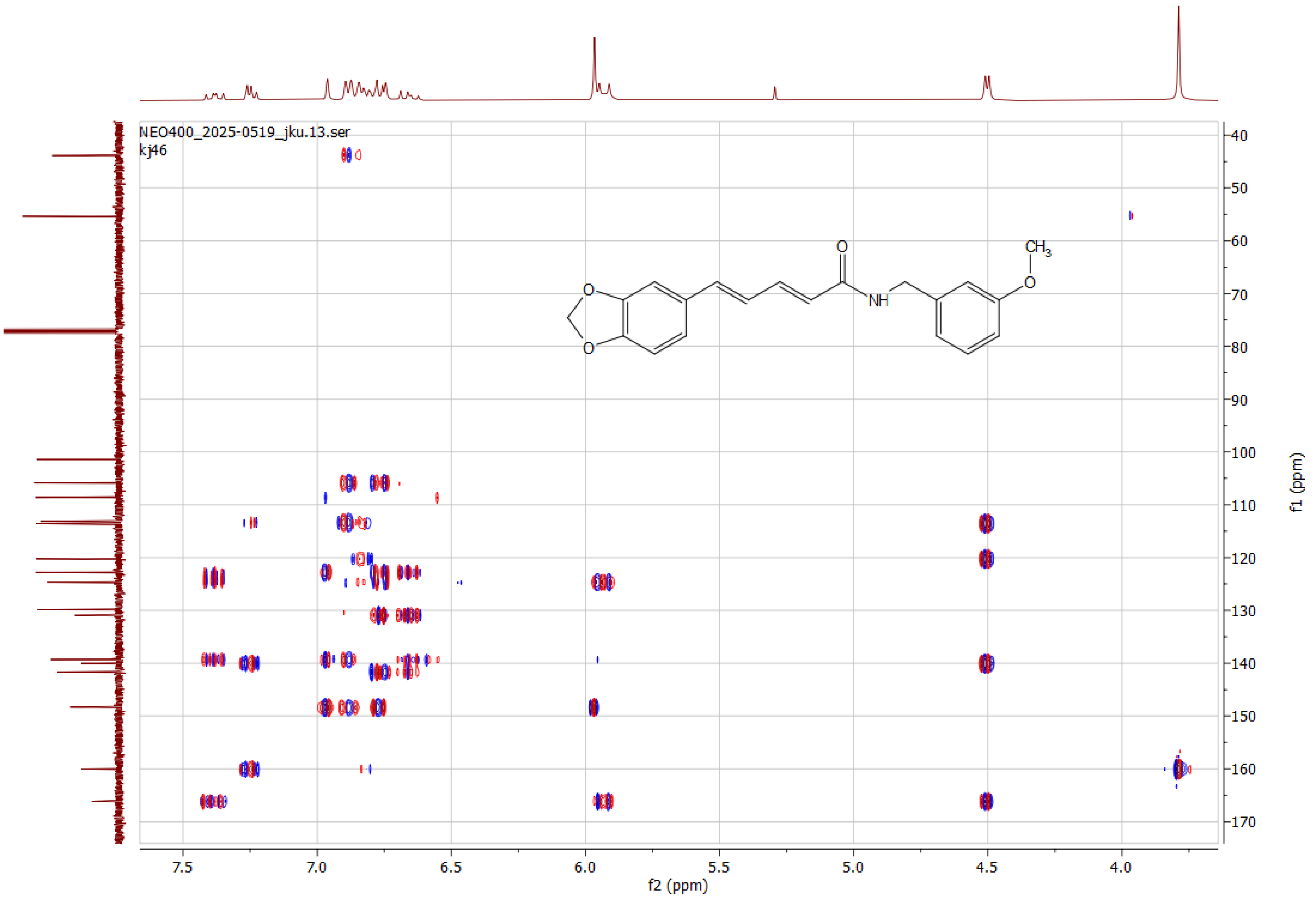

Figure S47: ^{1}H NMR (400 MHz, $CDCl_3$) of **7i**.

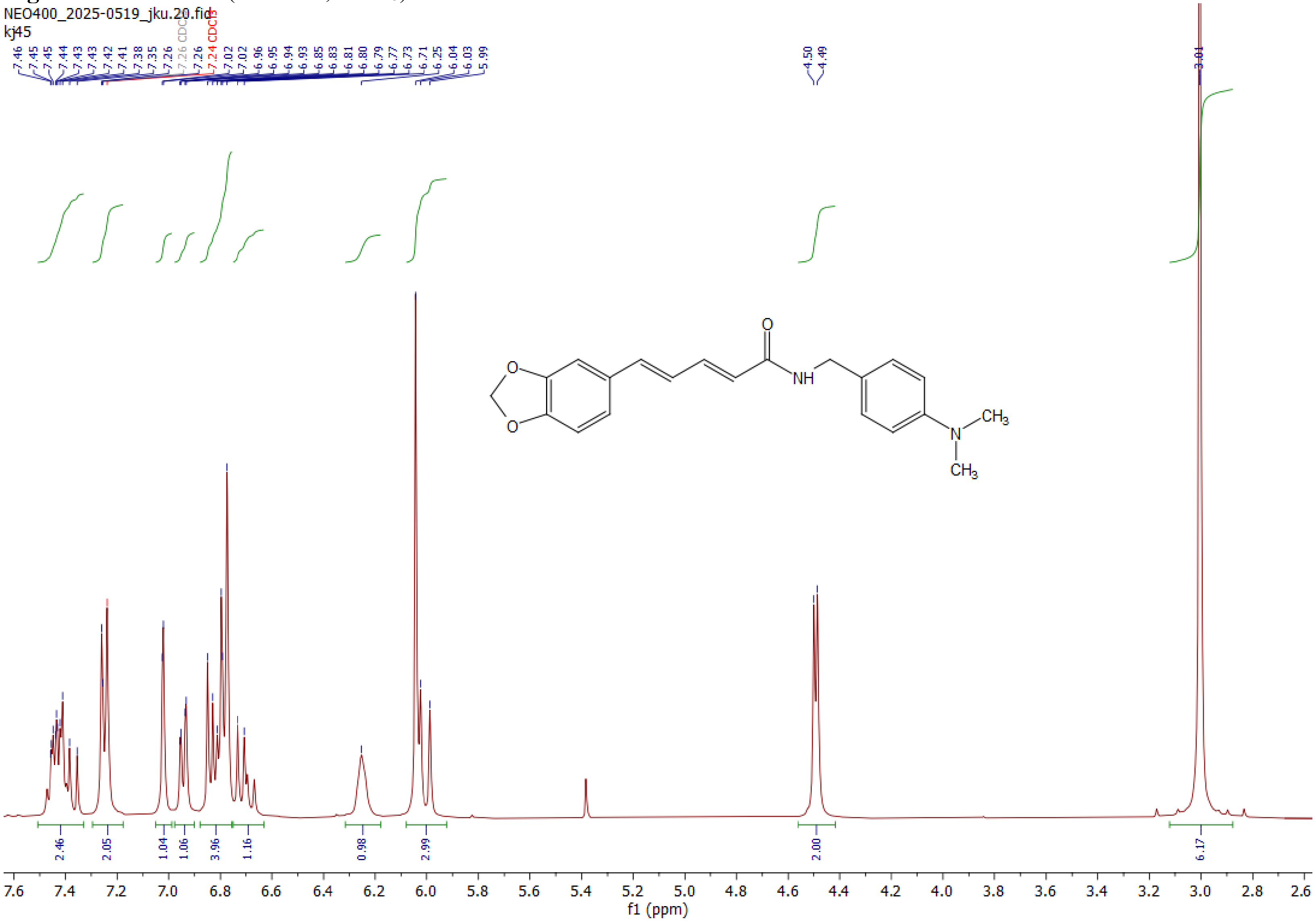

Figure S48: $^{13}C\{^1H\}$ NMR (100 MHz, $CDCl_3$) of **7i**.

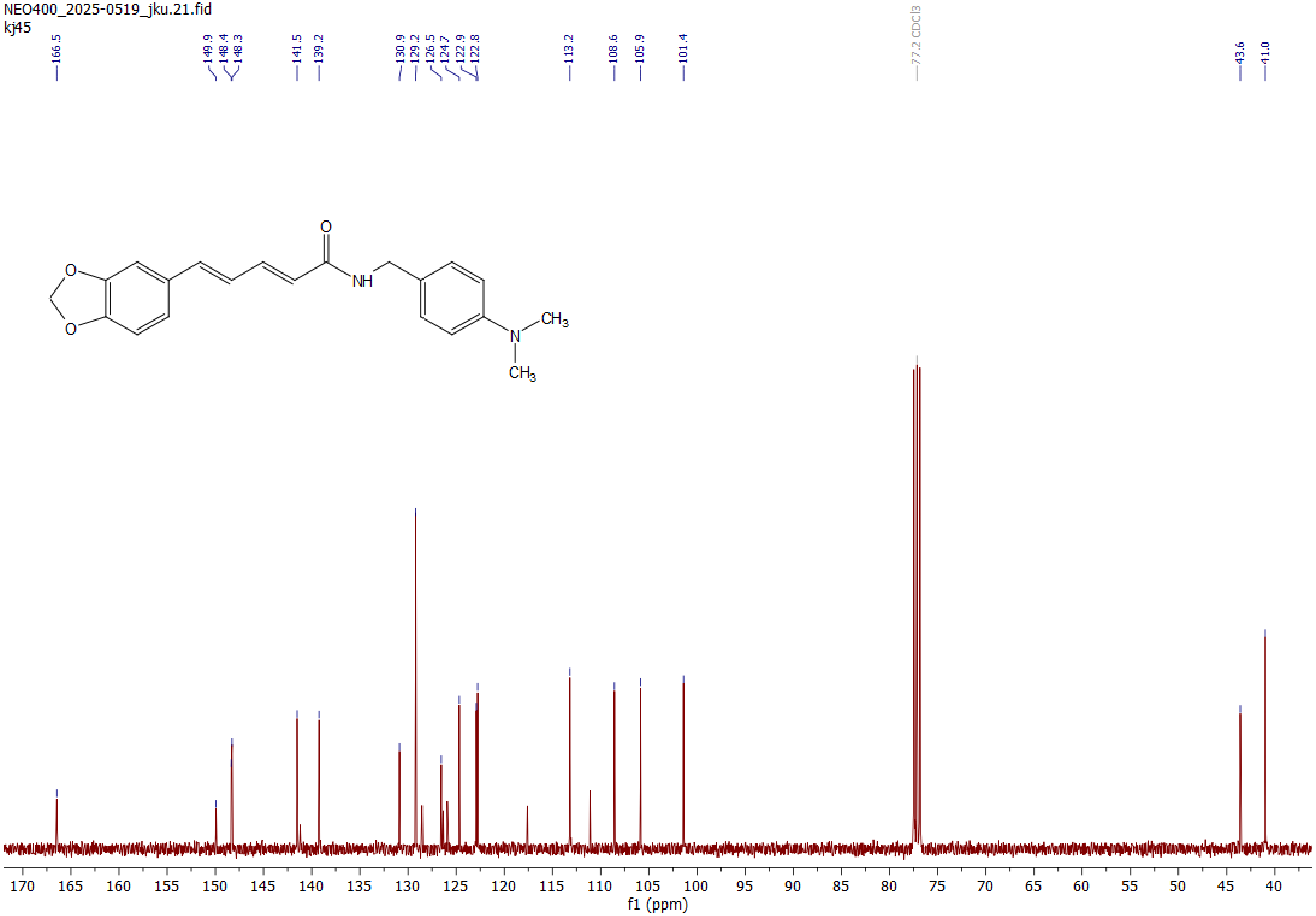

Figure S49: HSQC (400/100 MHz, $CDCl_3$) of **7i**.

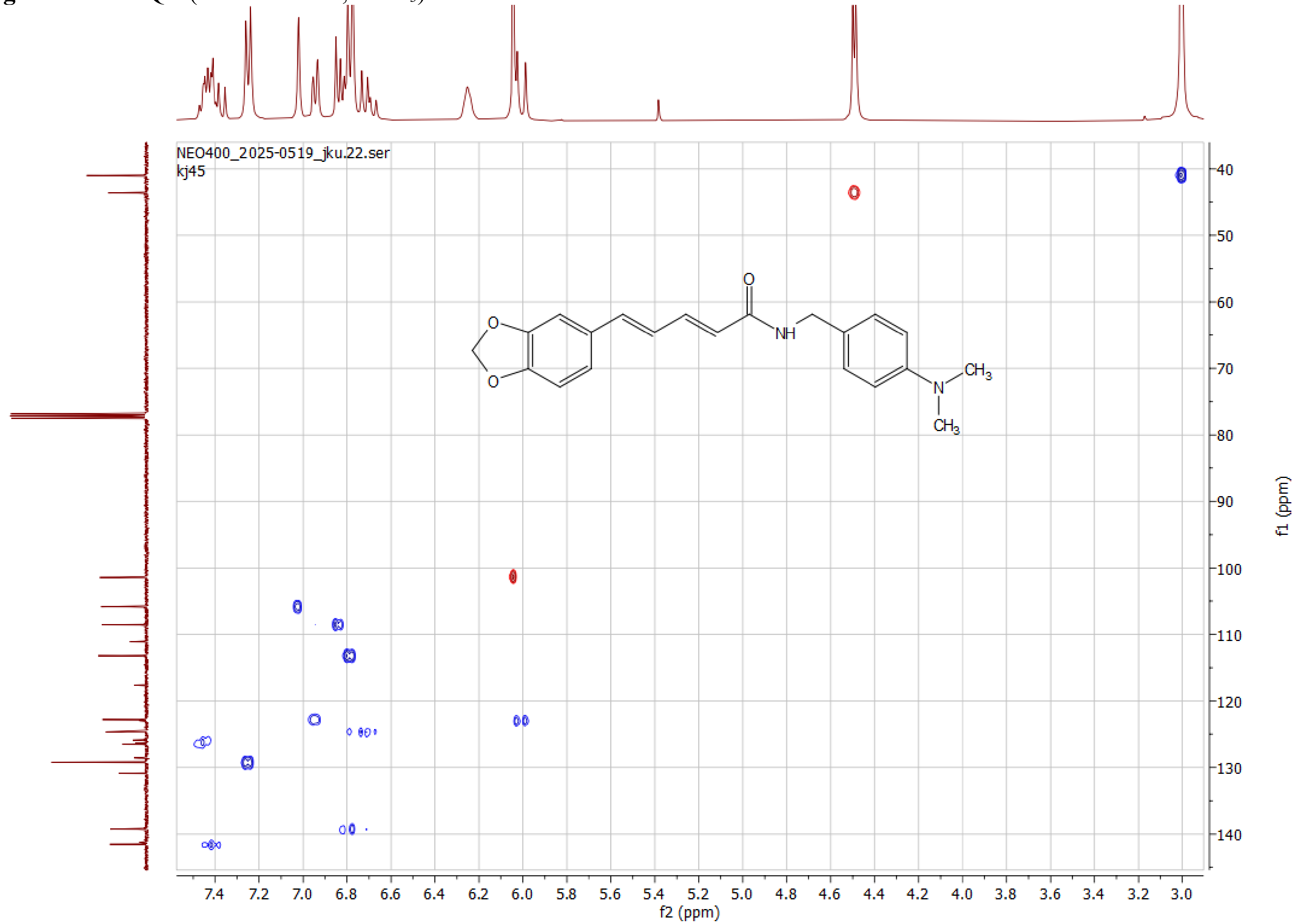

Figure S50: HMBC (400/100 MHz, $CDCl_3$) of **7i**.

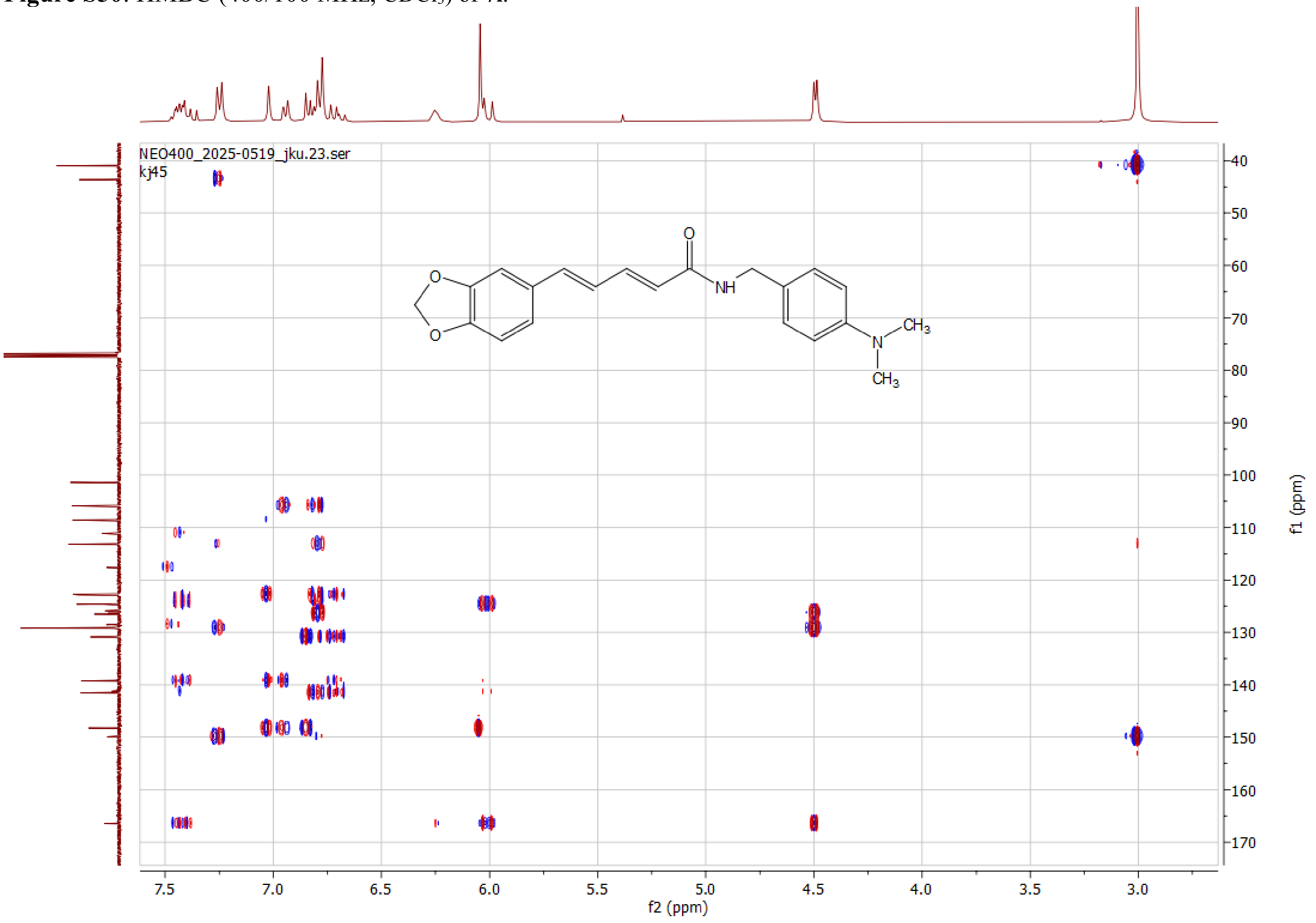

Figure S51: ^{1}H NMR (400 MHz, DMSO-d_6) of **7j**.

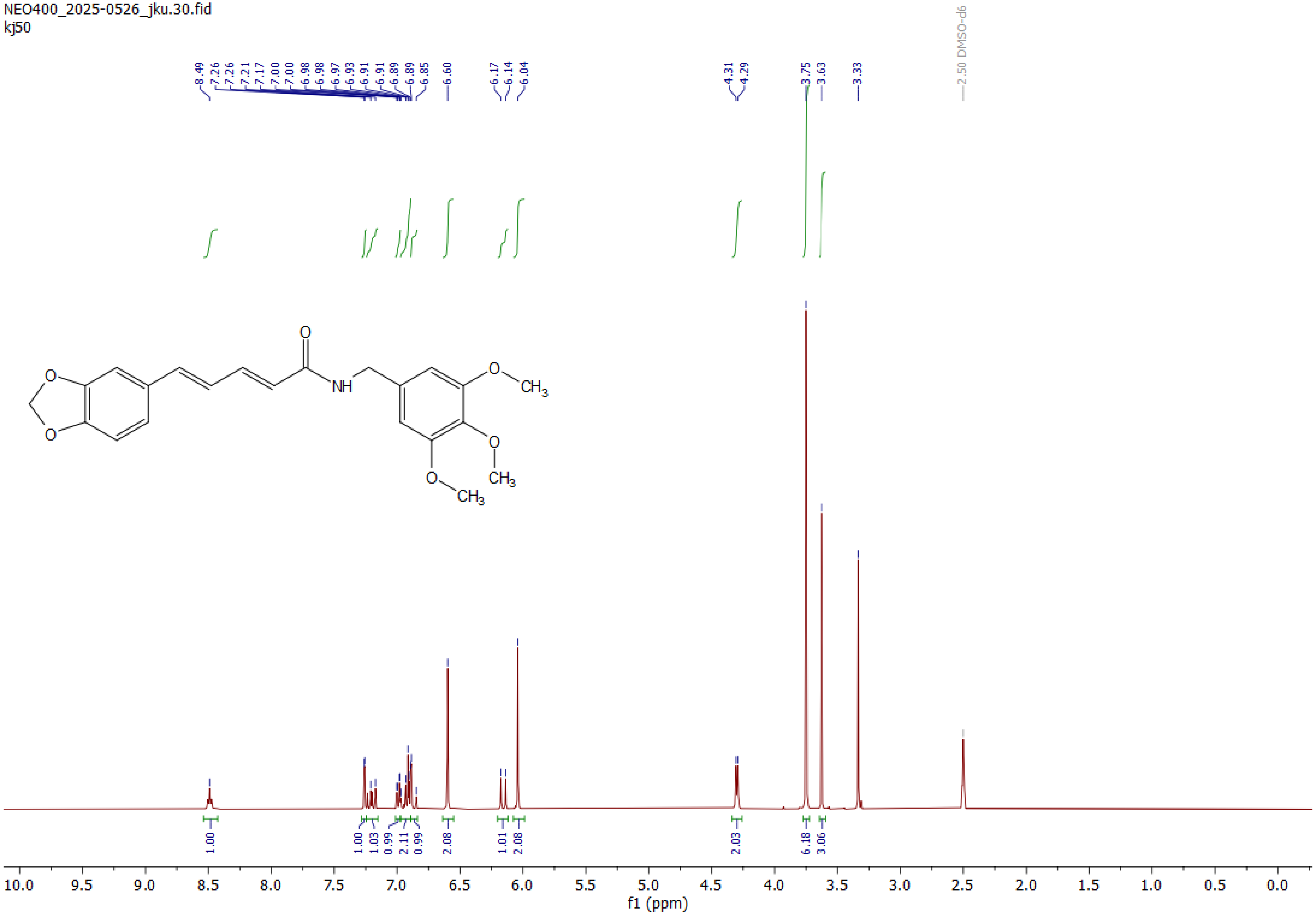

Figure S52: $^{13}C\{^{1}H\}$ NMR (100 MHz, DMSO-d_6) of **7j**.

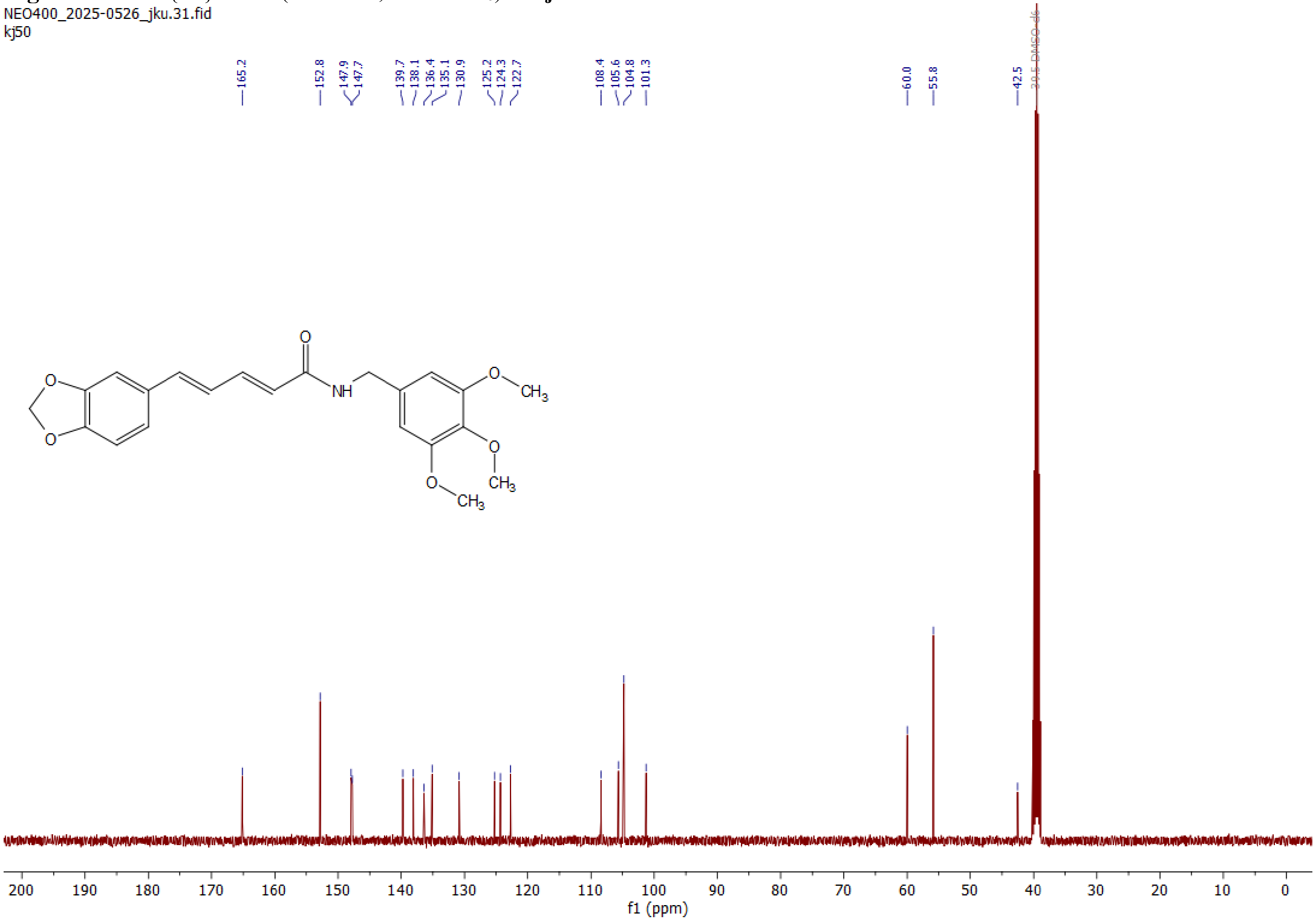

Figure S53: H,H-COSY (400 MHz, DMSO-d_6) of **7j**.

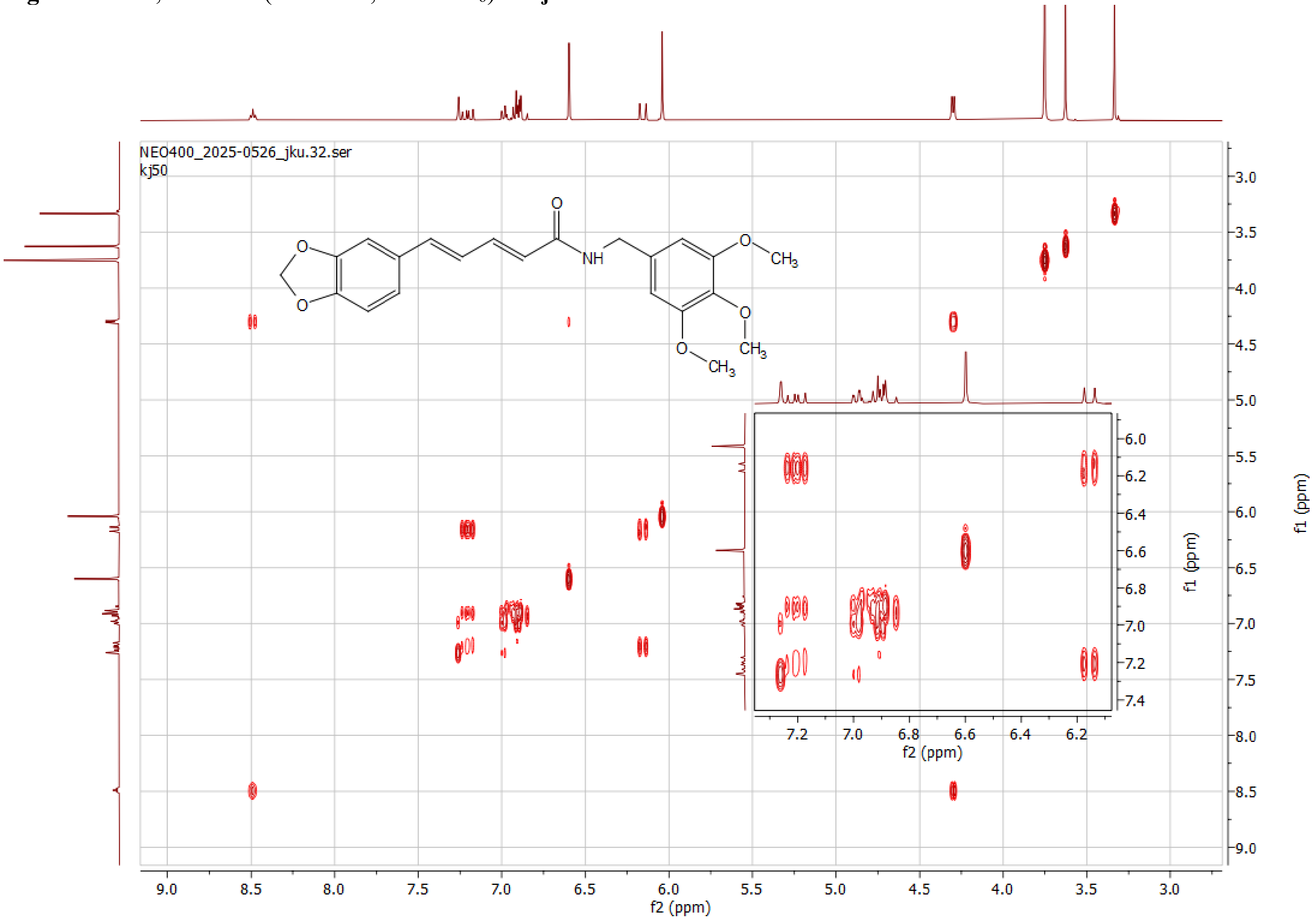

Figure S54: HSQC (400/100 MHz, DMSO-d_6) of **7j**.

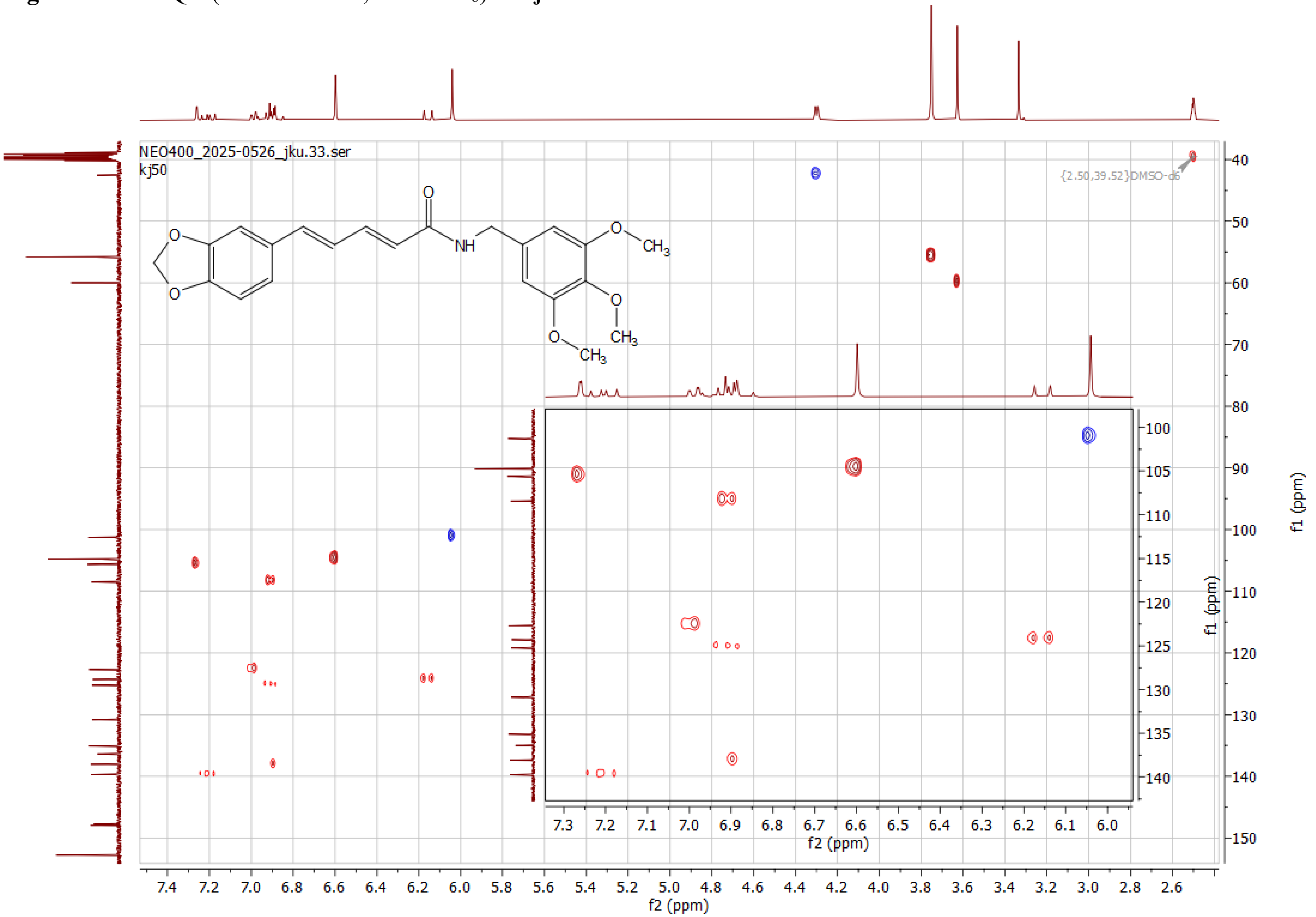

Figure S55: HMBC (400/100 MHz, DMSO-d_6) of **7j**.

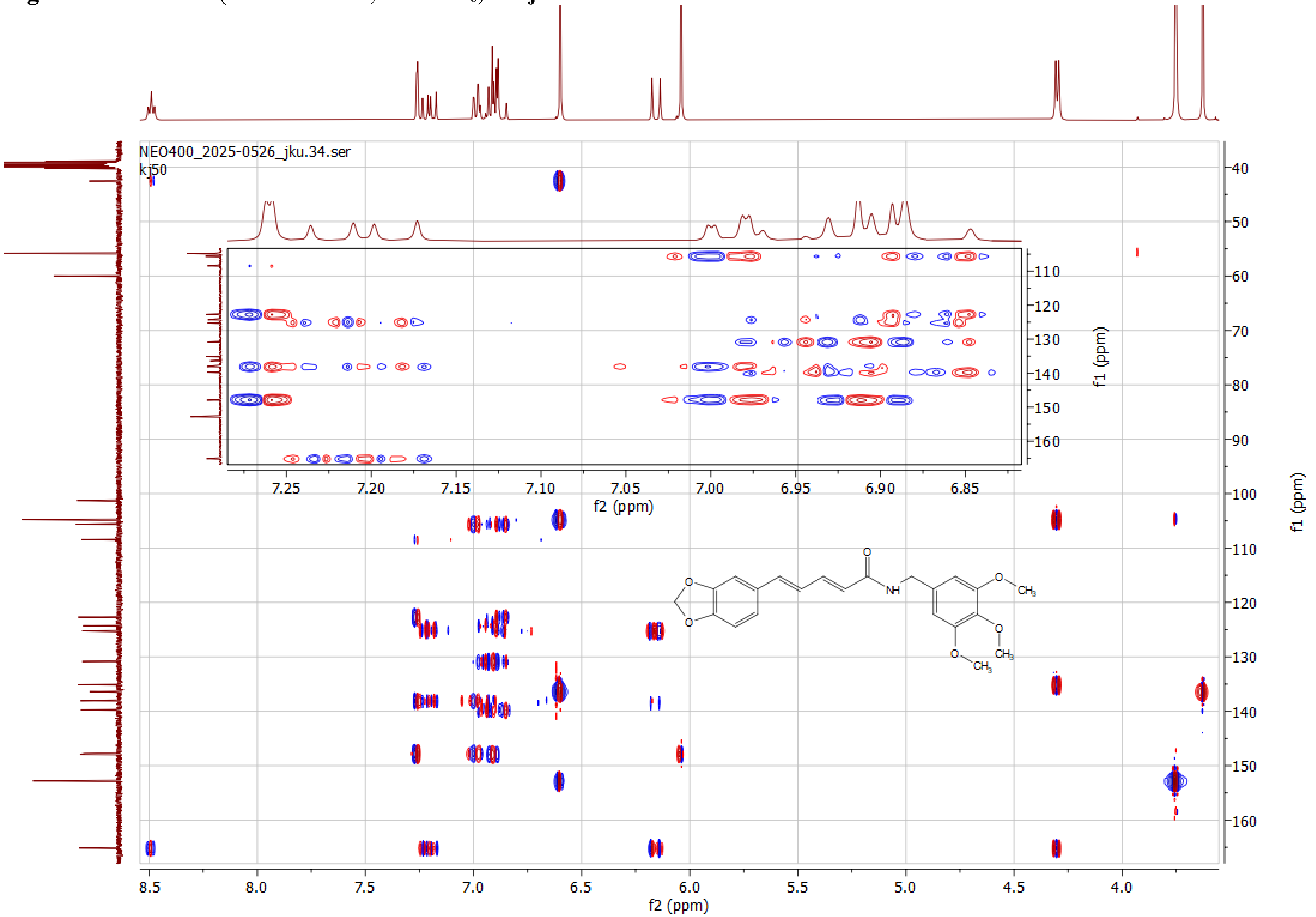

Figure S56: ^{1}H NMR (400 MHz, DMSO-d_6) of **7k**.

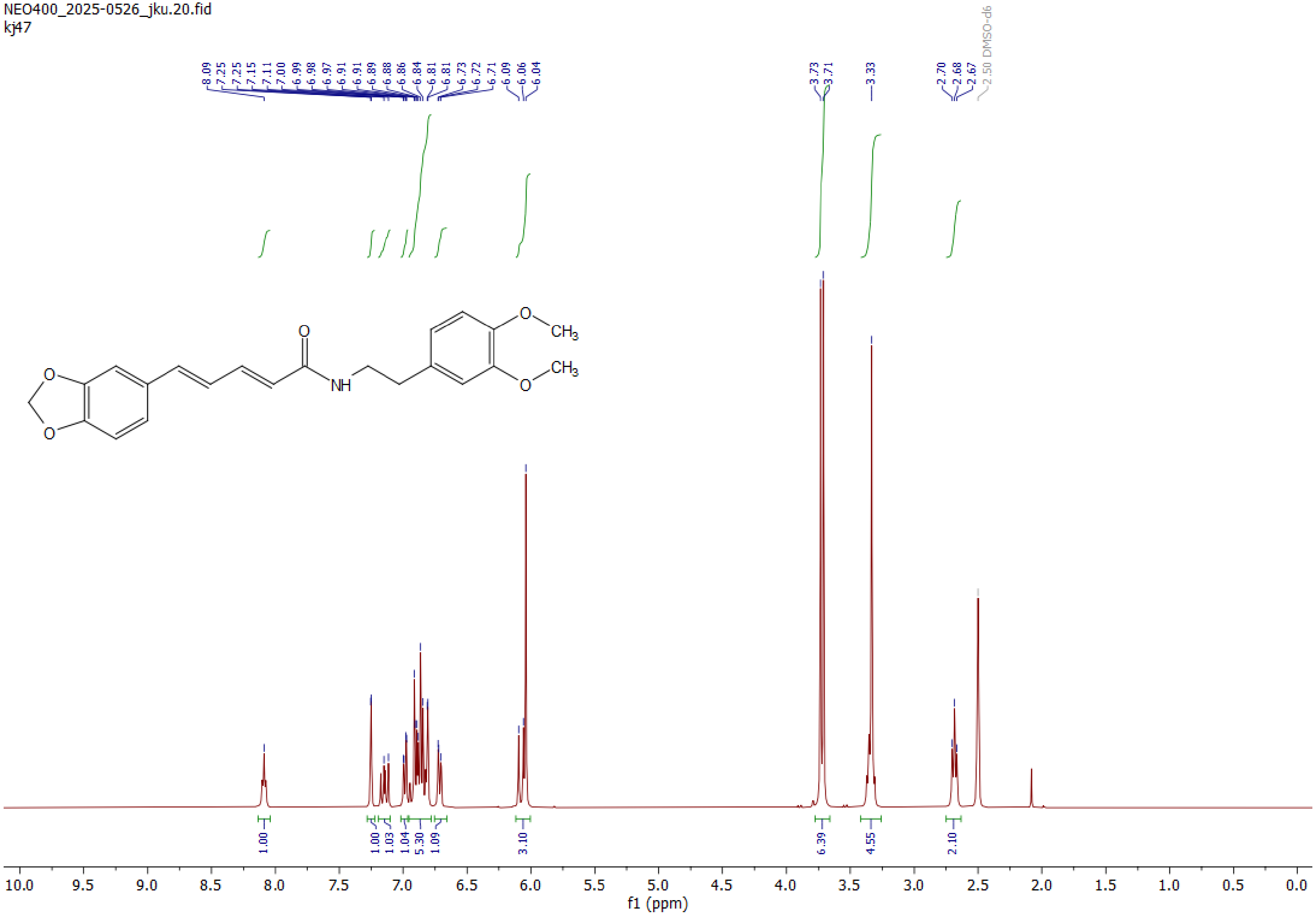

Figure S57: $^{13}C\{^1H\}$ NMR (100 MHz, DMSO-d_6) of **7k**.

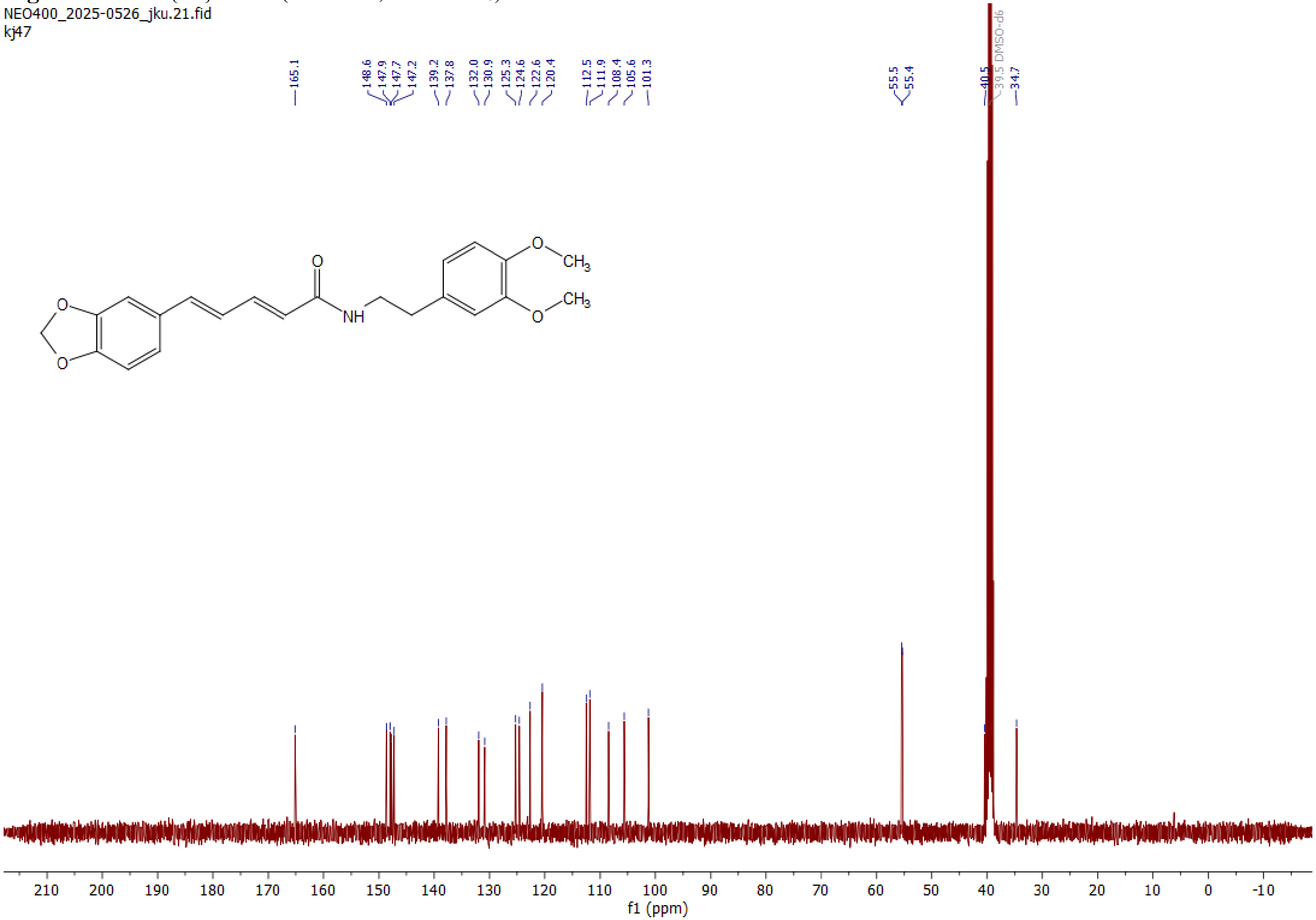

Figure S58: H,H-COSY (400 MHz, DMSO-d_6) of **7k**.

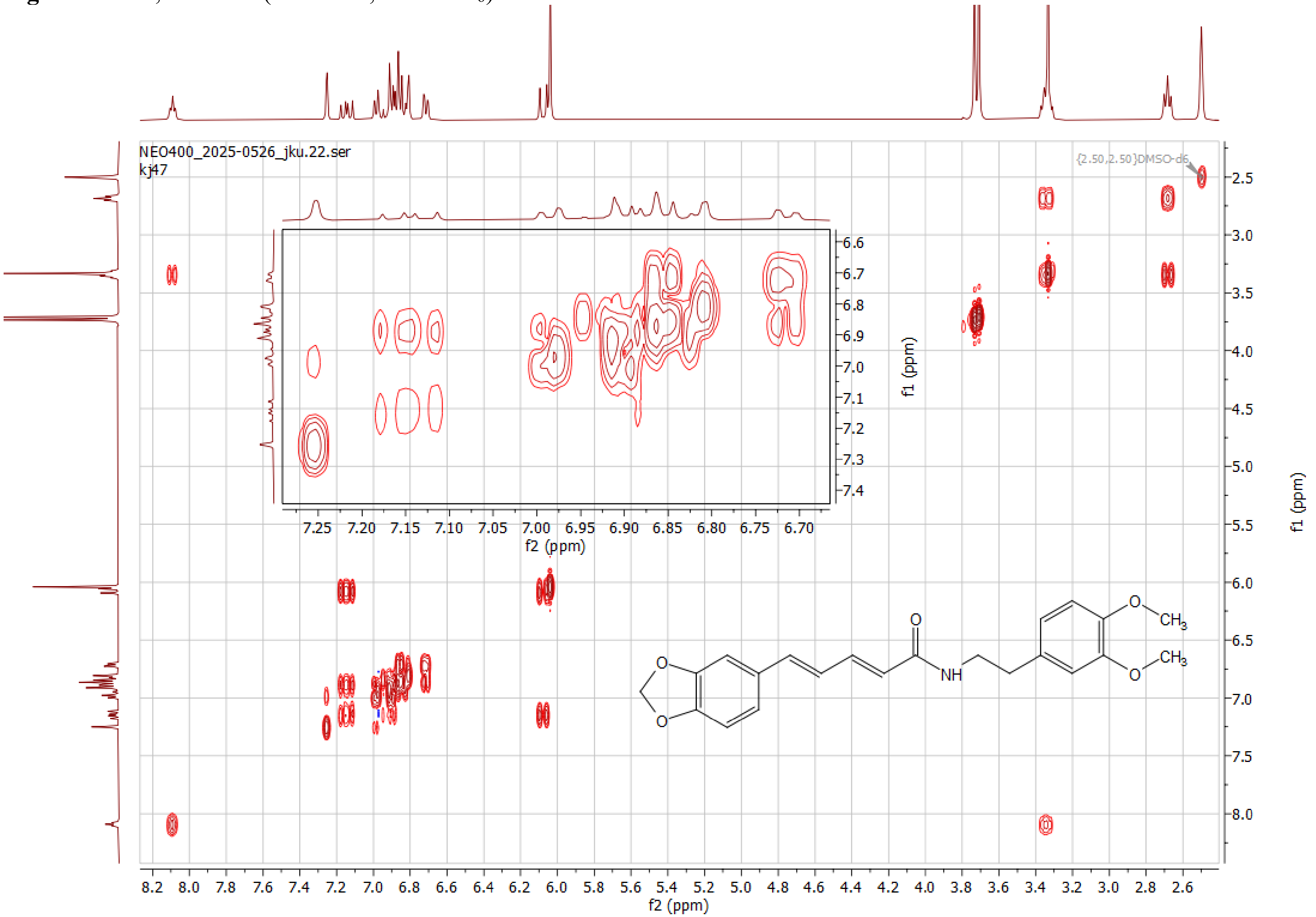

Figure S59: HSQC (400/100 MHz, DMSO-d_6) of **7k**.

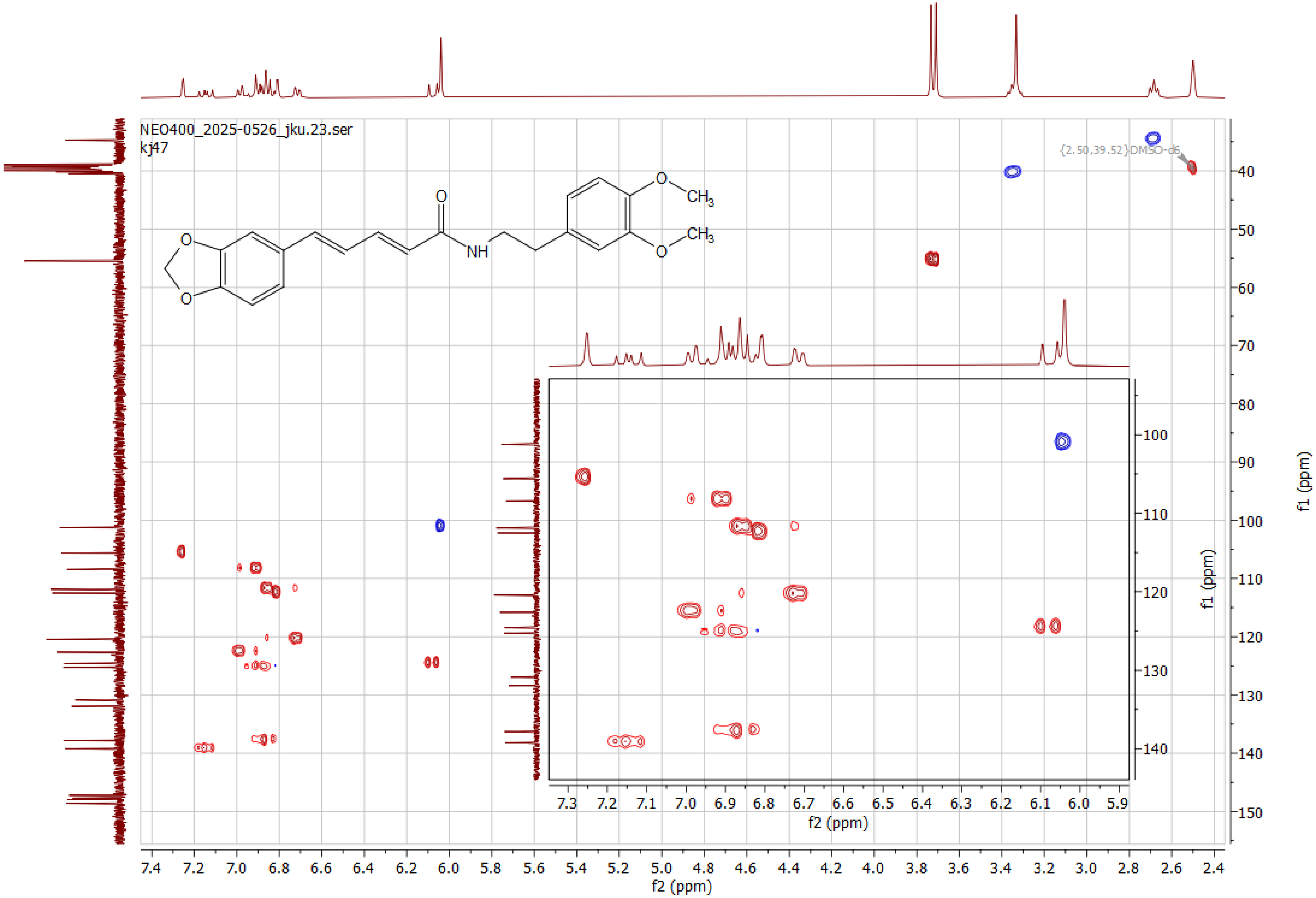

Figure S60: HMBC (400/100 MHz, DMSO-d_6) of **7k**.

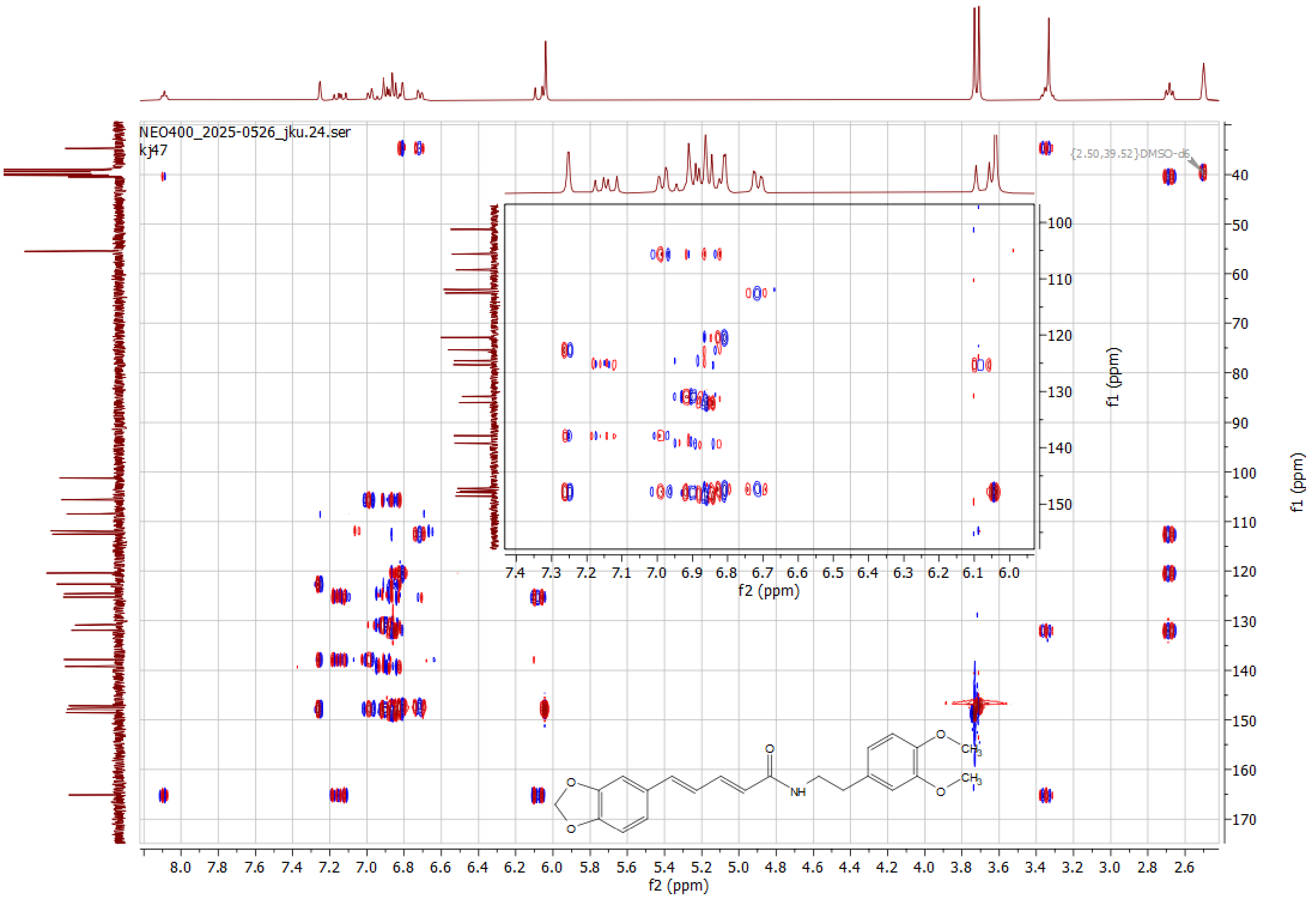

Figure S61: ^{1}H NMR (400 MHz, $CDCl_3$) of **7l**.

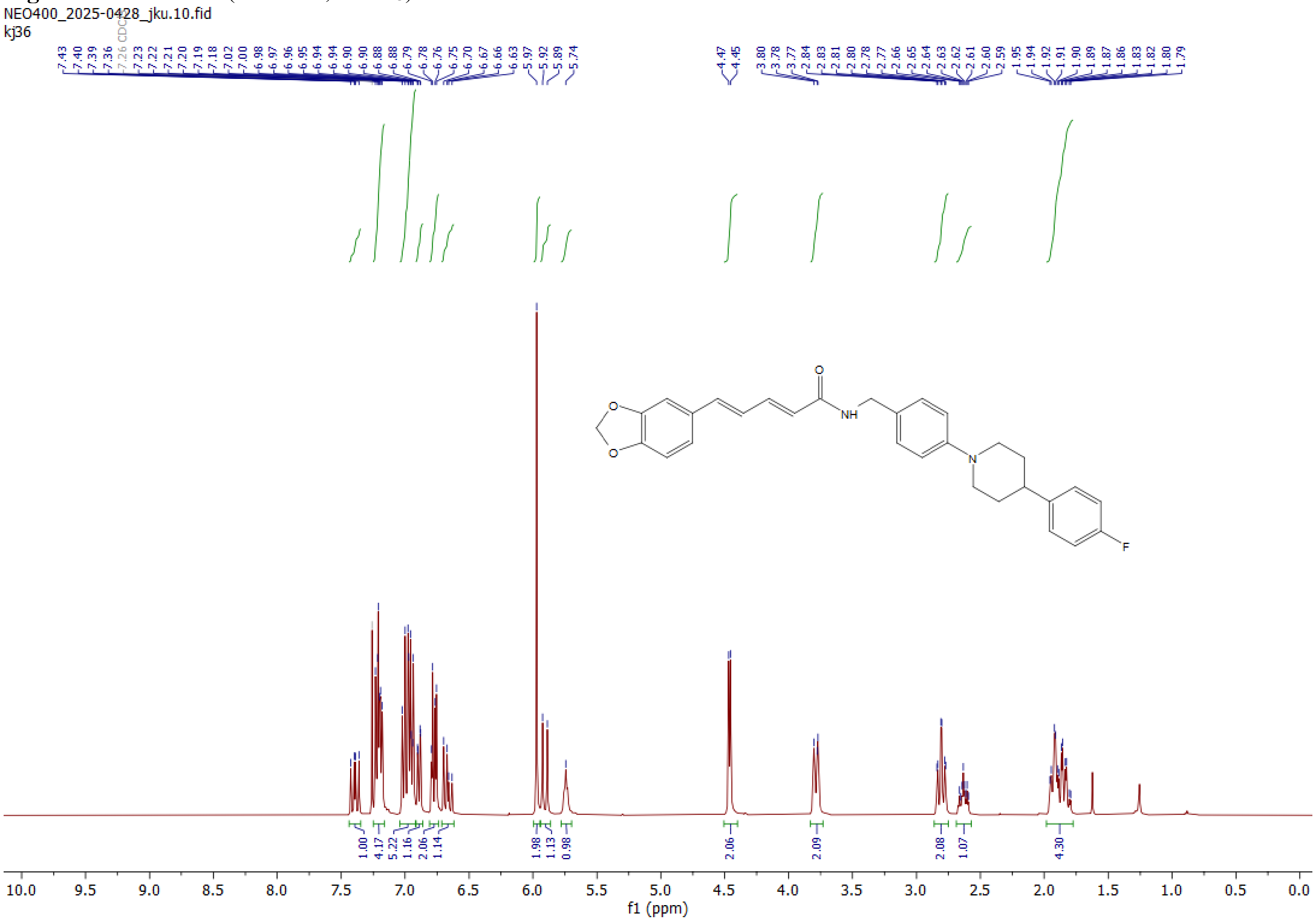

Figure S62: $^{13}C\{^1H\}$ NMR (100 MHz, $CDCl_3$) of **7l**.

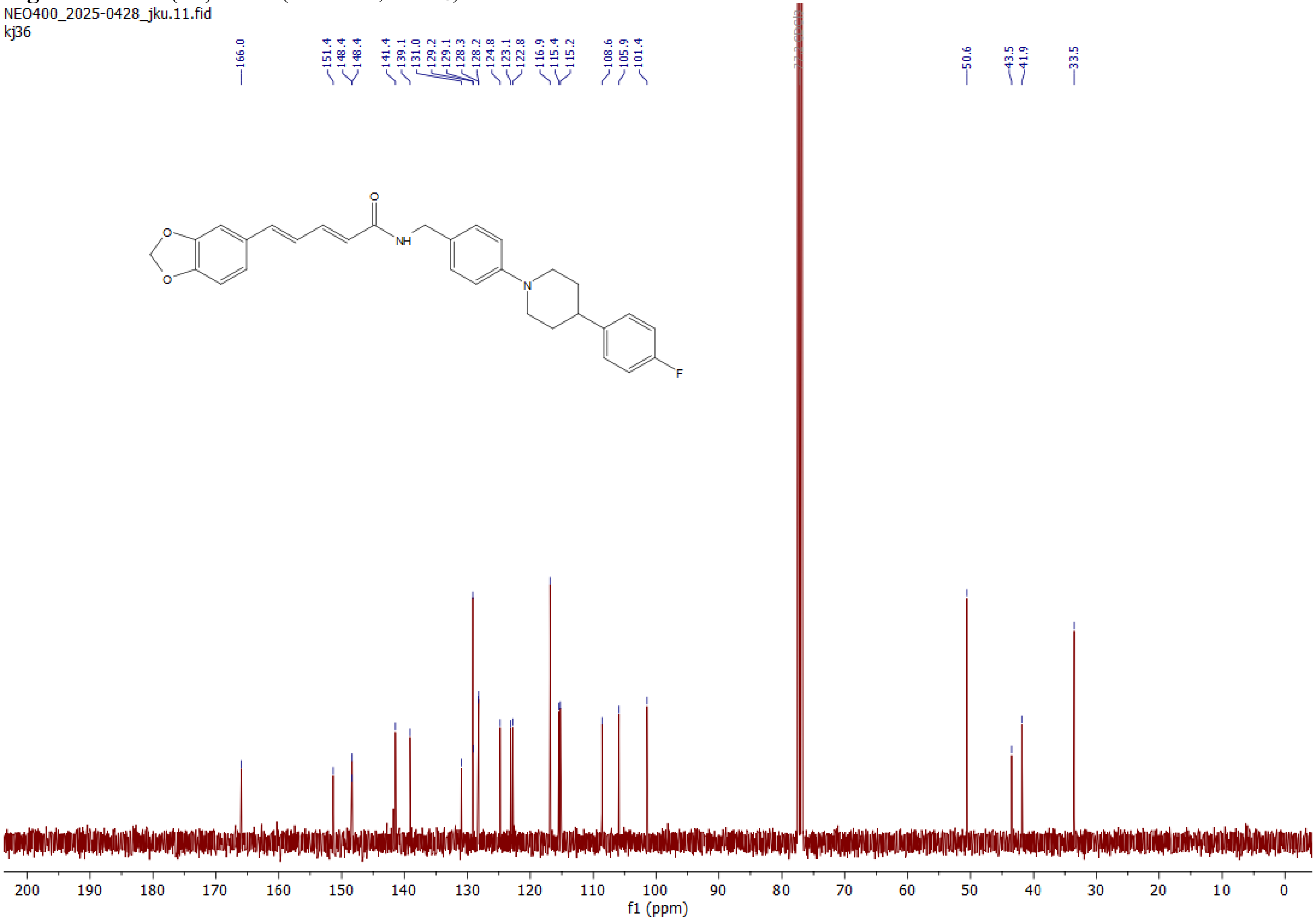

Figure S63: H,H-COSY (400 MHz, $CDCl_3$) of **7l**.

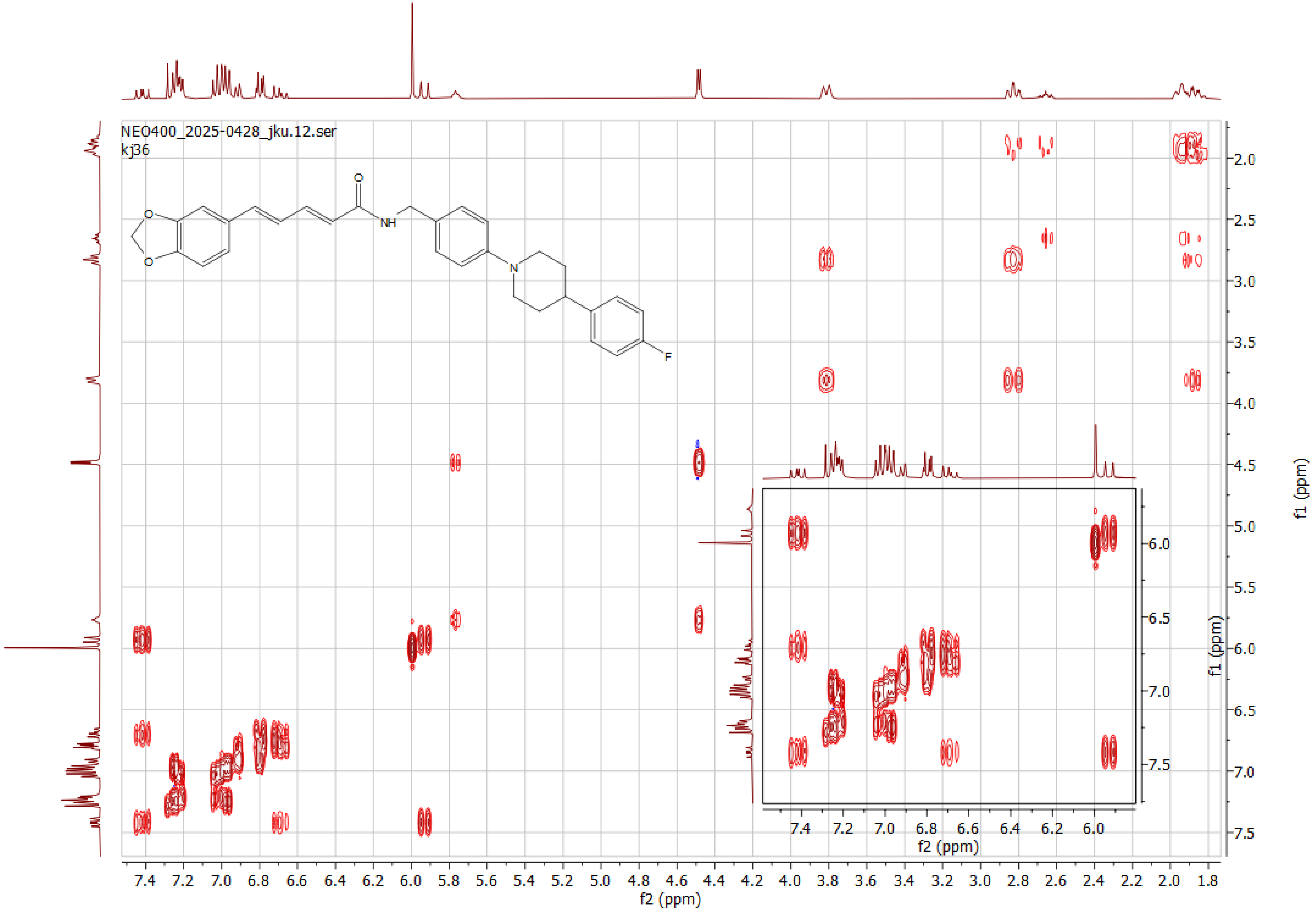

Figure S64: HSQC (400/100 MHz, $CDCl_3$) of **7l**.

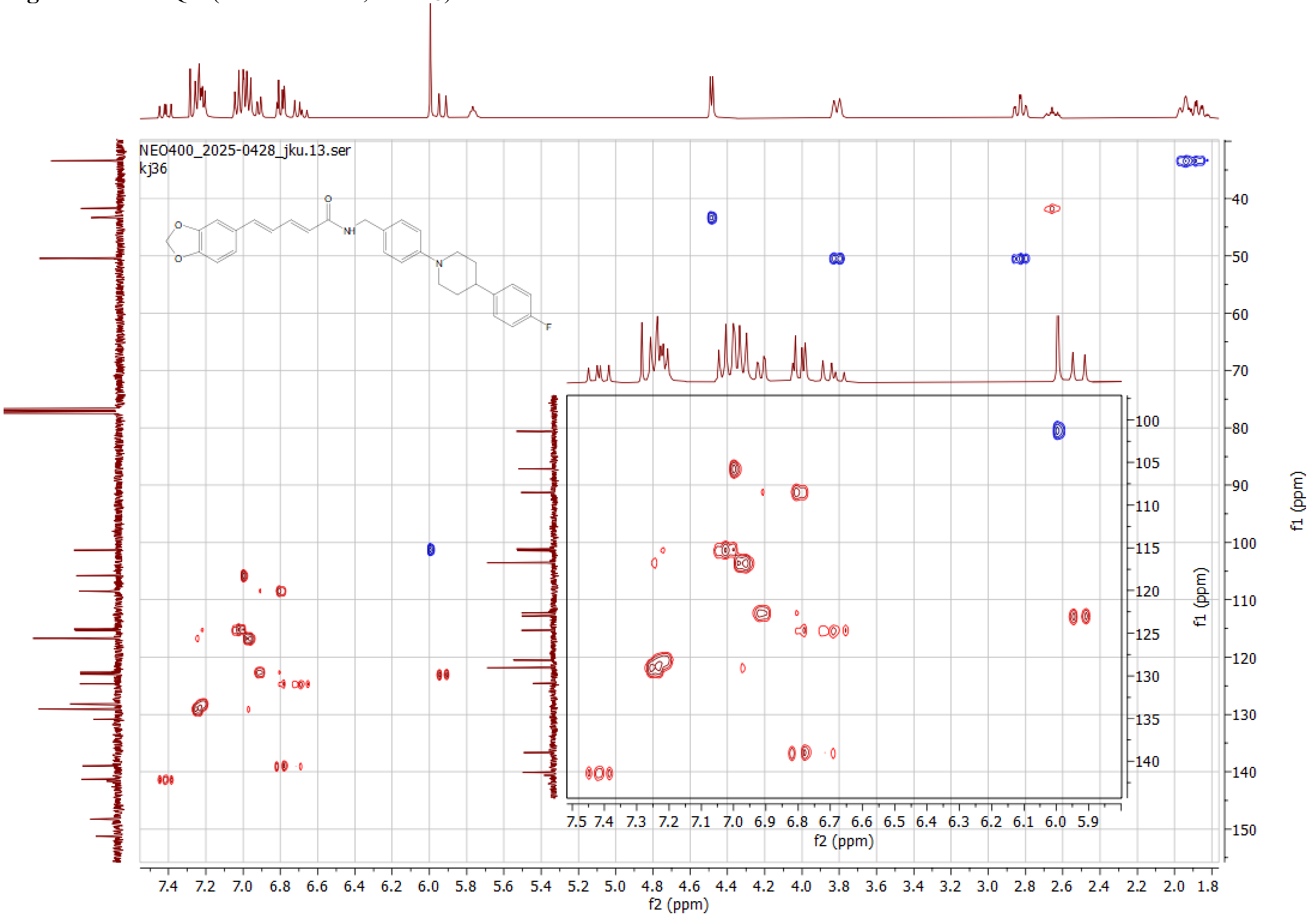

Figure S65: HMBC (400/100 MHz, $CDCl_3$) of **7l**.

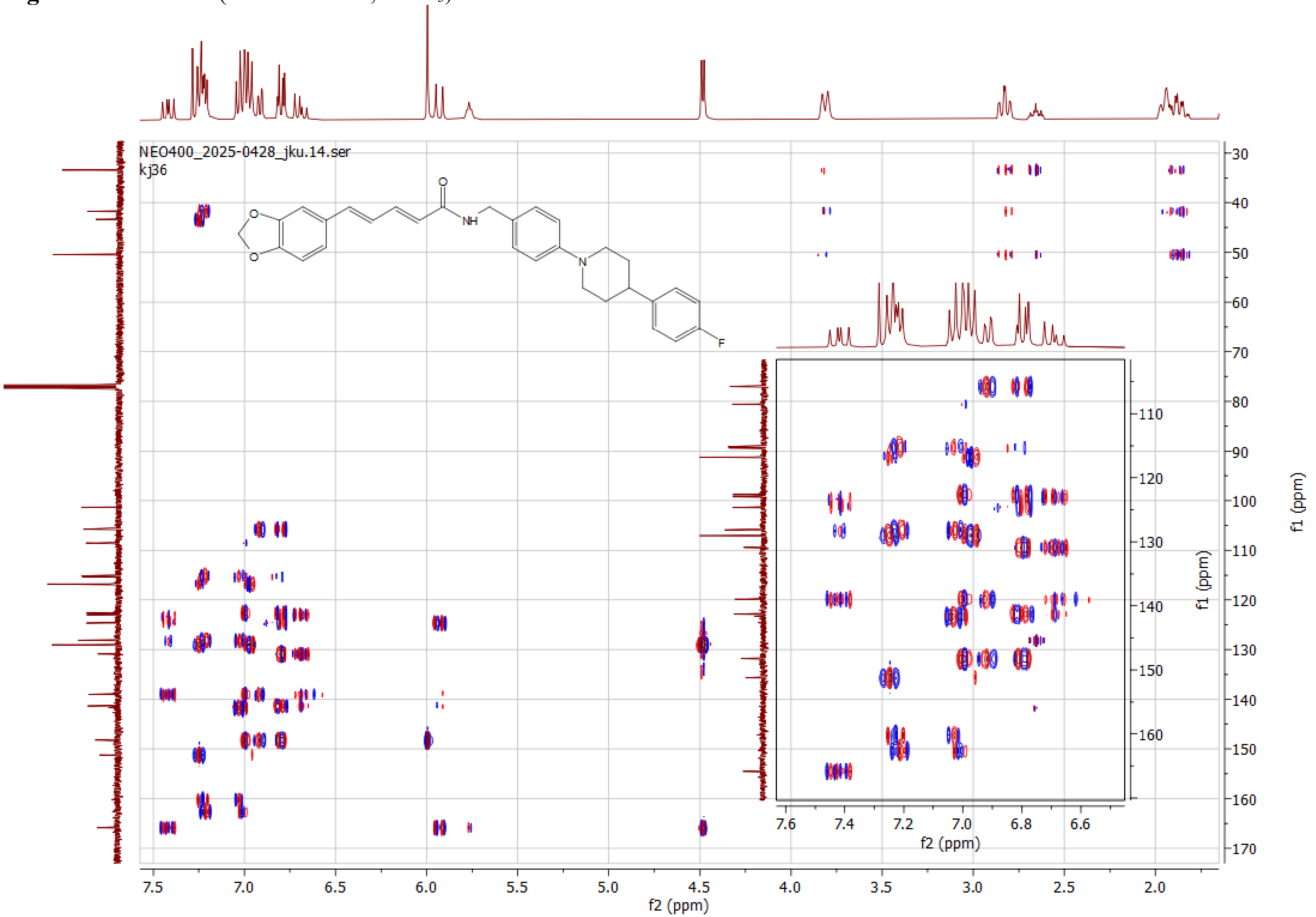

Table S2: Summary of Antimicrobial Activities of Compounds **5** and **7a-l**. Minimum inhibitory concentration (MIC) values in [µg/mL].

**Microorganisms**	**MIC (µg/mL)**													
	**5**	**7a**	**7b**	**7c**	**7d**	**7e**	**7f**	**7g**	**7h**	**7i**	**7j**	**7k**	**7l**	**References**
Schizosaccharomyces pombe DSM-70572	>66.6	66.6	>66.6	>66.6	>66.6	>66.6	>66.6	66.6	>66.6	>66.6	>66.6	>66.6	>66.6	4.2[N]
Wickerhamomyces anomalus DSM-6766	>66.6	>66.6	>66.6	>66.6	>66.6	>66.6	>66.6	>66.6	>66.6	>66.6	>66.6	>66.6	>66.6	4.2[N]
Mucor hiemalis DSM-2656	66.6	33.3	>66.6	>66.6	33.3	>66.6	>66.6	>66.6	>66.6	>66.6	>66.6	>66.6	>66.6	2.1[N]
Candida albicans DSM-1665	>66.6	>66.6	>66.6	>66.6	>66.6	>66.6	>66.6	>66.6	>66.6	>66.6	>66.6	>66.6	>66.6	2.1[N]
Rhodotorula glutinis DSM-10134	>66.6	16.6	>66.6	>66.6	33.3	>66.6	>66.6	>66.6	>66.6	>66.6	>66.6	>66.6	>66.6	1.0[N]
Acinetobacter baumannii DSM-30008	>66.6	>66.6	>66.6	>66.6	>66.6	>66.6	>66.6	>66.6	>66.6	>66.6	>66.6	>66.6	>66.6	0.52[C]
Echerichia coli DSM-1116	>66.6	>66.6	>66.6	>66.6	>66.6	>66.6	>66.6	>66.6	>66.6	>66.6	>66.6	>66.6	>66.6	0.42[G]
Bacillus subtilis DSM-10	>66.6	66.6	>66.6	>66.6	>66.6	>66.6	>66.6	>66.6	>66.6	>66.6	>66.6	>66.6	>66.6	16.6[O]
Mycobacterium smegmatis ATCC 700084	>66.6	66.6	>66.6	>66.6	66.6	>66.6	>66.6	>66.6	>66.6	>66.6	>66.6	>66.6	>66.6	0.1[K]
Staphylococcus aureus DSM-346	>66.6	>66.6	>66.6	>66.6	>66.6	>66.6	>66.6	>66.6	>66.6	>66.6	>66.6	>66.6	>66.6	0.42[G]
Pseudomonas aeruginosa DSM-PA14	>66.6	>66.6	>66.6	>66.6	>66.6	>66.6	>66.6	>66.6	>66.6	>66.6	>66.6	>66.6	>66.6	0.21[G]
Chromobacterium violaceum DSM-30191	66.6	66.6	>66.6	66.6	>66.6	>66.6	>66.6	>66.6	>66.6	>66.6	>66.6	>66.6	>66.6	0.83[G]

N : Nystatin 10 mg/mL ; C : Ciprobay 2.54 mg/mL ; G : Gentamycin 1 mg/mL ; O : Oxytetracyclin 1 mg/mL ; K : Kanamycin 1mg/mL
